# Photocatalytic multicomponent alkene dicarbofunctionalization *via* PCET/nickel dual catalysis

**DOI:** 10.1039/d6sc00382f

**Published:** 2026-02-19

**Authors:** Yeersen Patehebieke, Victoria Jansson, Jakob Öberg, Carl-Johan Wallentin

**Affiliations:** a Department of Chemistry and Molecular Biology, University of Gothenburg Gothenburg SE 41390 Sweden carl.wallentin@chem.gu.se

## Abstract

Herein we report a synergistic photoredox PCET/nickel dual catalytic strategy that enables three-component alkene dicarbofunctionalization (DCF) using unprotected alcohols as alkyl radical precursors. This transformation merges concerted proton-coupled electron transfer (PCET) activation of alcohols with nickel-catalyzed cross-coupling, achieving sequential Giese addition and C(sp^3^)–C(sp^3^), C(sp^3^)–C(sp^2^) bond formation under visible light and redox-neutral conditions. The method proceeds directly from secondary and tertiary alcohols, exhibits broad functional-group tolerance. Notably, employing a chiral Ni catalyst enables the first enantioselective variant of this type of PCET mediated transformation. This work establishes a general platform for deconstructive alkene difunctionalization from simple alcohol feedstocks, expanding the synthetic reach of PCET in dual catalytic radical chemistry.

## Abstract 2

Herein we report a synergistic photoredox PCET/nickel dual catalytic strategy that enables three-component alkene dicarbofunctionalization (DCF) using unprotected alcohols as alkyl radical precursors.

## Graphical abstract

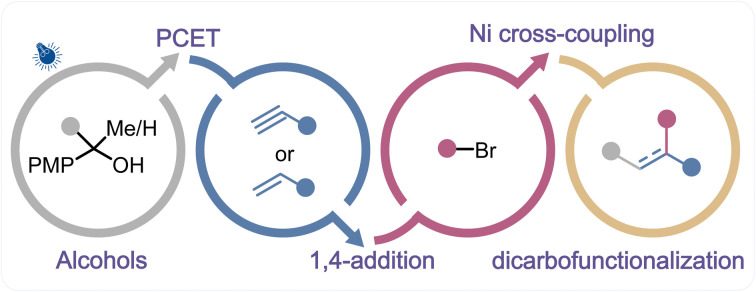

## Introduction

Multicomponent reactions (MCRs) provide a powerful platform for the rapid assembly of molecular complexity from simple precursors in a single operation.^1,2^ Their inherent convergence, efficiency, and atom economy have made them indispensable tools for the synthesis of pharmaceuticals, agrochemicals, and functional materials.^3^ By forming multiple bonds in a single step, MCRs minimize purification steps, reduce waste, and enable modular access to structurally diverse scaffolds. These features align MCRs with the principles of sustainable chemistry^4^ and make them highly attractive for late-stage diversification in medicinal chemistry.^5^ The challenge, however, lies in controlling selectivity when multiple reactive intermediates are generated simultaneously. Recent advances in catalysis, particularly visible-light photoredox catalysis, have opened new avenues to address this challenge by enabling the controlled generation of reactive radical intermediates under exceptionally mild and tunable conditions.^6–8^

**Fig. 1:**
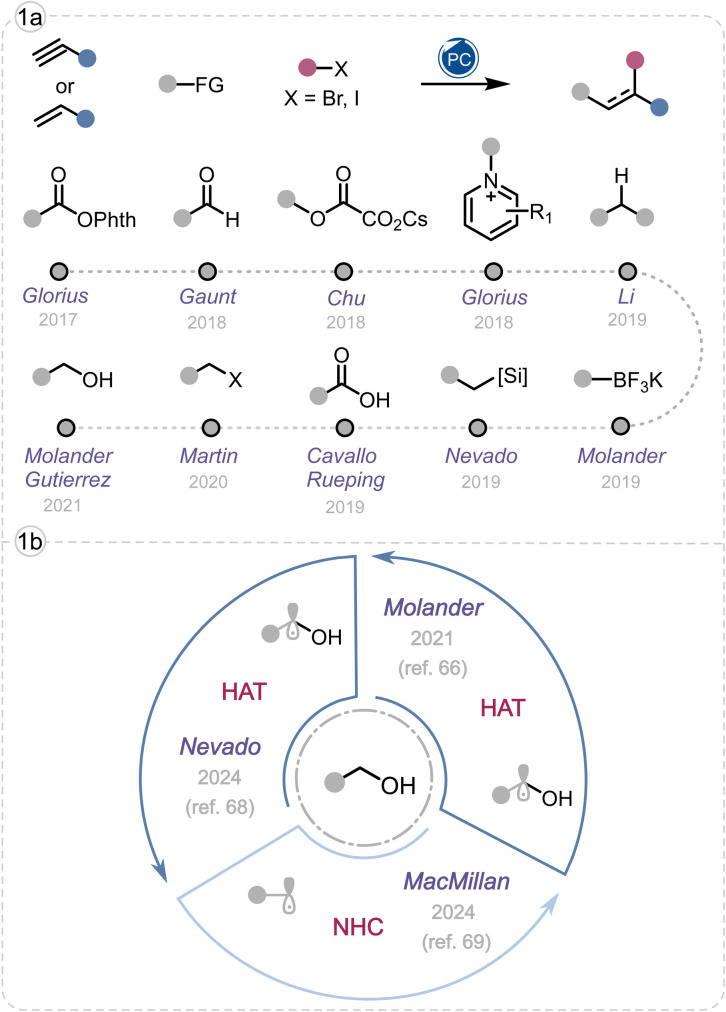
(a) Representative alkyl radical precursors in photocatalytic multicomponent reactions. (b) Photocatalytic Ni-catalyzed multicomponent reactions using alcohols as alkyl radical precursors.

Photoredox catalysis relies on single-electron transfer (SET) processes from photoexcited catalysts to transform simple precursors into radicals with excellent control over reactivity and selectivity.^9^ As a result, photoredox catalysis has greatly expanded the scope of multicomponent transformations, enabling the direct incorporation of diverse alkyl fragments into complex molecular frameworks. Precursors such as carboxylic acids,^10–15^ alkyltrifluoroborates,^16–28^ oxalates,^29–32^*N*-(acyloxy)phthalimides (NHPI esters),^33–44^ alkyl halides,^45–53^ silicates,^54–58^ Katritzky pyridinium salts,^59–61^ aldehydes,^62–65^ alcohols,^66–70^ and even unactivated C(sp^3^)–H bonds^66,68,71–75^ have been converted into carbon-centered radicals and engaged in sequential bond-forming events with high functional-group tolerance (Fig. 1a).

These developments have transformed MCRs into versatile tools for step-economic construction of C–C and C–heteroatom bonds and have set the stage for increasingly sophisticated radical cascades. Among the various strategies for radical generation, proton-coupled electron transfer (PCET) has emerged as a particularly powerful approach for activating strong X–H bonds under mild, redox-neutral conditions.^76,77^ By orchestrating electron transfer and proton transfer in a single concerted event, PCET overcomes the high bond dissociation energies of substrates such as alcohols (BDE ≈ 105 kcal mol^−1^), enabling direct access to alkoxy or carbon-centered radicals without prefunctionalization.^76,78^ Pioneering studies by Knowles,^79–83^ and others^84–91^ established the foundation for PCET activation of alkanols, enabling ring-opening reactions and Giese-type additions that rapidly build molecular complexity. These breakthroughs demonstrated that alcohols can serve as practical radical precursors, circumventing the need for laborious derivatization steps. More recently, PCET also has been integrated into dual catalytic systems, where the radicals generated from alcohols are intercepted by nickel, unlocking cross-coupling pathways that were previously inaccessible.^92–95^

**Fig. 2:**
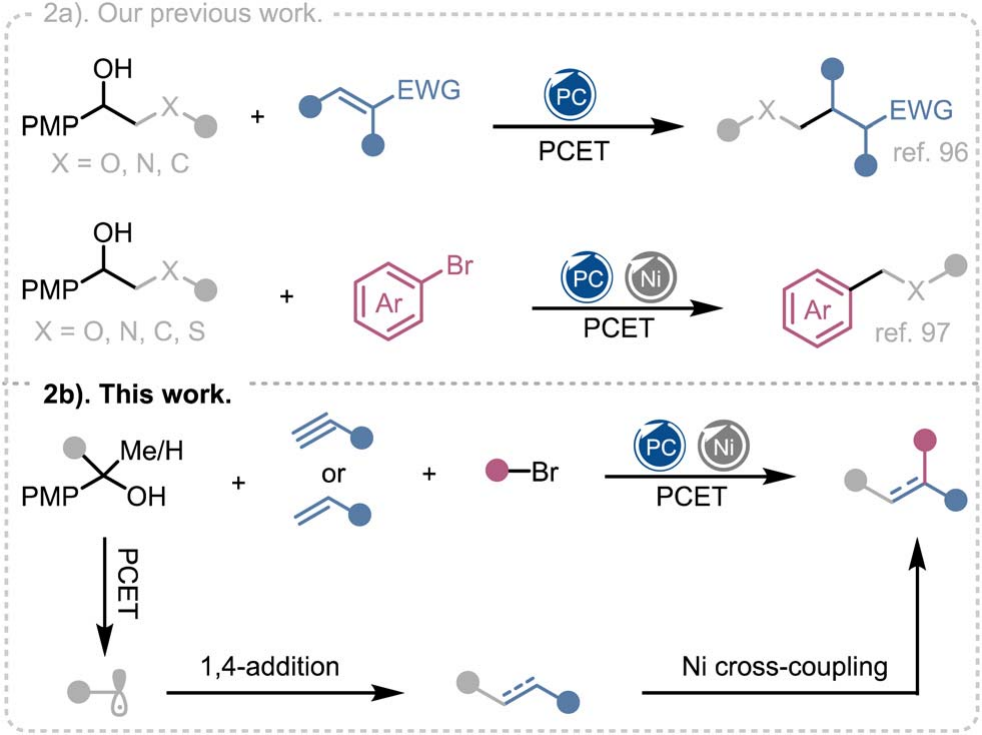
(a) Our previous work on PCET mediated alkyl radical generation from alcohol and its utilization. (b) This work: photoredox PCET/nickel dual catalytic three-component alkene dicarbofunctionalization by alcohols.

Recently, our group developed a PCET based metal-free photocatalytic strategy for the β-scission of secondary and tertiary alcohols, enabling their direct conversion into alkyl radicals under visible light irradiation.^96^ This protocol allowed the efficient alkylation of diverse electron-deficient alkenes and demonstrated late-stage functionalization of pharmaceutically relevant scaffolds under mild, redox-neutral conditions (Fig. 2a). In subsequent work, we merged concerted PCET-mediated radical generation with nickel cross-coupling to achieved a practical deconstructive arylation of aliphatic alcohols (Fig. 2a).^97^ Together, these studies established that free alcohols can serve as versatile radical precursors for both Giese-type additions and Ni-catalyzed cross-couplings, thereby laying the foundation for combining these two manifolds in a single multicomponent transformation.

A few photocatalytic three-component reactions that combine radical 1,4-addition with nickel-catalyzed cross-coupling have been reported as powerful methods for vicinal difunctionalization of alkenes under mild conditions.^98,99^ In these processes carbon-centered radicals are derived from pre-activated species like NHPI esters,^37–39,42,43^ oxalates,^29–31^ or trifluoroborates^16,18–20,24–28^ which require multistep synthesis and limit late-stage applicability. Direct use of free alcohol in such three-component transformations remains rare (Fig. 1b).

In 2021, Molander and Gutierrez presented photoredox/nickel dual catalytic utilization of alcohols (among other feedstocks like ethers and amides) by performing photocatalyzed hydrogen atom transfer (photo-HAT) to activate aliphatic C–H bonds giving alkyl radicals. The subsequent steps involve Giese addition of the alkyl radicals and nickel-catalyzed coupling.^66^ Later in 2024, Nevado realized a similar alkene dicarbofunctionalization *via* an enantioselective photo-HAT/DCF process.^68^ In the same year, the MacMillan group utilized benzoxazolium activated primary alcohols as alkyl radical precursors in photoredox/nickel dual catalytic DCF of alkenes. The reaction proceeds through a triple radical sorting mechanism that merges photoredox and nickel-catalyzed bimolecular homolytic substitution (*S*_H_2__) catalysis to achieve alkene dialkylation.^69^

There has not been any report that utilize PCET in photoredox/nickel dual catalytic DCF of alkenes. Combining PCET-mediated radical generation from unactivated alcohols would allow for the direct use of secondary and tertiary alcohols in DCF type transformations. Consequently, a PCET based approach would deliver a more general and modular three-component strategy towards molecular construction.

Herein, we report a synergistic photoredox PCET/nickel dual catalytic system that accomplishes the three-component dicarbofunctionalization of electron-deficient alkenes using unprotected alcohol as the radical source (Fig. 2b). The method features broad substrate scope, including challenging secondary and tertiary alcohols, and demonstrates excellent functional-group tolerance. We also demonstrated the first enantioselective variant of a PCET initiated DCF process.

## Results and discussion

To evaluate whether our previously developed PCET-mediated alkyl radical generation from alcohols is compatible with three-component alkene dicarbofunctionalization, we selected tertiary alcohol 1, phenyl acrylate 2, and 4-bromoacetophenone 3 as model substrates. The reaction conditions were adapted from our previously reported nickel-catalyzed deconstructive cross-coupling methods.^97^ Using the Fukuzumi catalyst [Mes–Acr–Me]^+^ClO_4_^−^ as the photocatalyst, NiBr_2_·dtbbpy (4,4′-di-*tert*-butyl-2,2′-dipyridyl) as cross-coupling catalyst, 2,4,6-collidine as the base, in a solvent mixture of 1,2-dichloroethane (DCE) + 5% MeCN at 35 °C under 450 nm blue light irradiation for 48 h. To our delight, the desired dicarbofunctionalized product 4 was obtained in a very good yield of 82% (Table 1, entry 1).

**Table 1:** Optimization of reaction conditions^a^

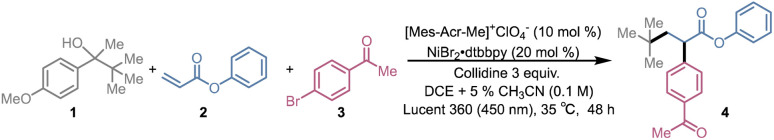		
Entry	Deviation from standard conditions	Yield^b^
1	No deviation	82%
2	DCE	73%
3	MeCN	n.d.
4	DCM	57%
5	DMF	Trace
6	[Mes–Acr–Me]^+^BF_4_^−^	81%
7	6 h	73%
8	12 h	81%
9	12 h, 100% intensity	74%
10	6 h, 75% intensity	74%
11	12 h, 75% intensity	84%
12	12 h, no photocatalyst	n.d.
13	12 h, no nickel catalyst	n.d.
14	12 h, no base	n.d.
15	12 h, no light	n.d.

a Reaction conditions: alcohol (0.3 mmol), alkene (0.2 mmol), aryl bromide (0.1 mmol), 2,4,6-collidine (0.3 mmol), [Mes–Acr–Me]^+^ClO_4_^−^ (001 mmol), NiBr_2_·dtbbpy (0.02 mmol), DCE + 5% MeCN (3 mL), time (48 h), Lucent 360 photo reactor (450 nm, 50% light intensity), temperature (35 °C).

b Yields were determined by ^1^H NMR analysis of the crude reaction mixtures using ethylene carbonate as an internal standard.

Encouraged by this result, we performed additional screening to refine the conditions. Using DCE alone reduced the yield to 73% (entry 2), while MeCN as the sole solvent failed to produce the desired product (entry 3). Interestingly, emplying MeCN as a co-solvent improved the yield, possibly due to an optimal balance of polarity and Brønsted basicity that promotes both precursor complex formation and disaggregation of the successor complex, thereby mitigating non-productive back electron transfer.^100^ Other solvents gave significantly lower or trace yields (entries 4–5). Altering the reaction stoichiometry negatively impacted the outcome (see SI for details). Changing the photocatalyst counterion from ClO_4_^−^ to BF_4_^−^ had little effect (entry 6), and screening other acridinium photocatalysts resulted in decreased yields (see SI for details).

Shortening the reaction time to 6 h slightly reduced the yield to 73% (entry 7), with ^1^H NMR indicating residual starting alcohol. At 12 h, the alcohol was nearly consumed, and the yield improved to 81% (entry 8). Increasing light intensity to 100% accelerated alcohol consumption within 12 h but lowered the product yield to 74% (entry 9), likely due to increased side-product formation and photocatalyst degradation. Reducing light intensity to 75% and maintaining a 12 h reaction time afforded the highest yield of 84% (entry 11). Finally, control experiments confirmed the essential role of all components—photocatalyst, nickel catalyst, base, and light—as omission of any resulted in no product formation (entries 12–15).

**Scheme 1:**
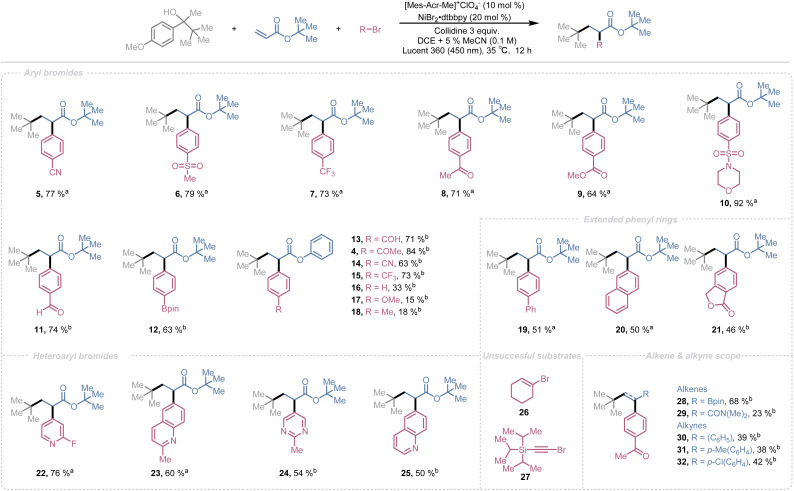
Scope of aryl halides, alkenes and alkynes. Reaction conditions: alcohol (0.3 mmol), alkene (0.2 mmol), aryl bromide (0.1 mmol), 2,4,6-collidine (0.3 mmol), [Mes–Acr–Me]^+^ClO_4_^−^ (0.01 mmol), NiBr_2_·dtbbpy (0.02 mmol), DCE + 5% MeCN (3 mL), time (12 h), Lucent 360 photo reactor (450 nm, 75% light intensity), temperature (35 °C). ^*a*^Isolated yield. ^*b*1^H NMR yield.

With the optimal reaction conditions established, we next explored the substrate scope of this transformation. We began by examining the aryl bromide scope. A wide range of aryl bromides bearing diverse functional groups were well tolerated under optimized conditions (Scheme 1). Substrates containing nitrile 5, sulfone 6 & 10, trifluoromethyl 7, ketone 8, ester 9, aldehyde 11, and boronic ester groups 12 afforded the desired products in good to excellent yields (92–63%). Notably, aryl bromides bearing aldehyde 11 or Bpin 12 groups posed purification challenges, as the target products co-eluted with the ketone byproduct and trace amounts of unreacted alcohol.

To address this, we replaced *tert*-butyl acrylate with phenyl acrylate, anticipating improved separation. Phenyl acrylate delivered good yields (84–63%) with electron-withdrawing substituents on the aryl halide 4, 13–15, however, electron-neutral 16 and electron-donating substituents 17 & 18 significantly diminished the yields. This limitation is consistent with known trends in photoredox/nickel cross-coupling reactions, where efficient oxidative addition to low-valent nickel species requires a polarized C–X bond and an electrophilic aryl carbon. Electron-donating groups reduce electrophilicity, slowing oxidative addition and increasing decomposition pathways.^101^ Unfortunately, switching to phenyl acrylate did not resolve the purification issue, so we reverted to *tert*-butyl acrylate as the alkene partner.

Extended aromatic systems such as biphenyl 19, naphthalene 20, and phthalide derivatives 21 provided decent yields (51–46%). Heteroaryl bromides, including pyridine 22, quinoline 23 & 25, and pyrimidine derivatives 24, also furnished good to moderate yields (76–50%). Attempts to employ alkyl bromides 26 or alkynyl bromides 27 as coupling partners were unsuccessful, with reactions failing under the optimized conditions.

After achieving satisfactory results with aryl halides, we investigated the scope of alkenes and alkynes. Electron-deficient olefins such as vinylboronic acid pinacol ester 28 and *N*,*N*-dimethylacrylamide (DMAA) 29 exhibited reactivity comparable to acrylates, delivering good to synthetically useful yields. Ethynylbenzene derivatives bearing electron-neutral 30, electron-donating 31, and electron-withdrawing substituents 32 also afforded reasonable yields.

**Scheme 2:**
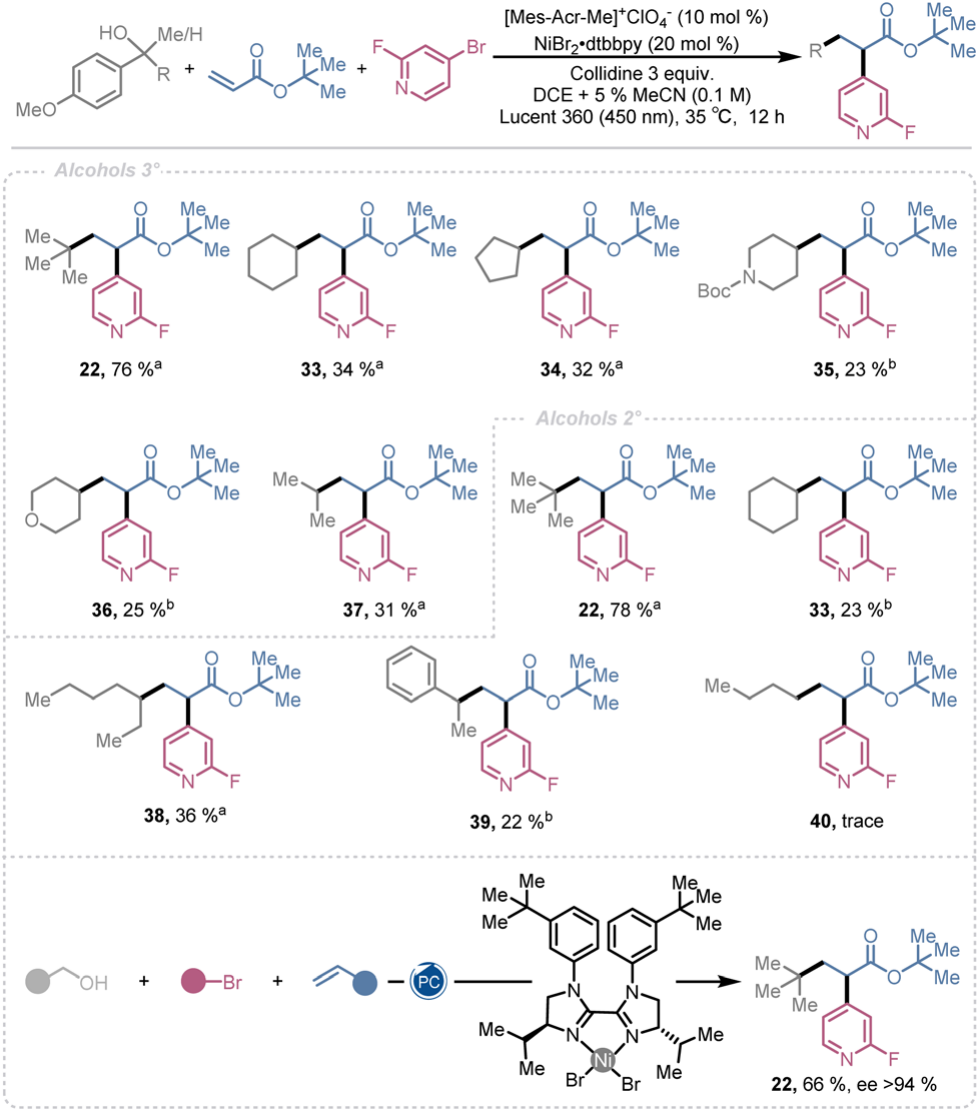
Scope of alcohols. ^*a*^Isolated yield. ^*b*1^H NMR yield.

Finally, we examined the alcohol scope for alkyl radical generation (Scheme 2). Tertiary alcohols bearing alkyl, cycloalkyl, and heterocyclic groups, including *tert*-butyl 22, cyclohexyl 33, cyclopentyl 34, piperidine 35, tetrahydropyran 36, and isopropyl 37, provided dicarbofunctionalized products in yields ranging from 76% to 23%. Secondary alcohols with tertiary 22, secondary 33 & 38, benzylic 39, and primary 40 substituents exhibited similar trends, affording products in 78% to trace yield. The observed reactivity correlates with radical stability, following the order 3° > 2° > benzylic > 1°. More stable and sterically hindered radicals favor Giese-type addition to the olefin, after which the resulting radical engages the nickel catalytic cycle to furnish the difunctionalized product.

In contrast, less stable and less hindered radicals are more prone to nickel-mediated cross-coupling, competing with Giese addition and thereby reducing overall yields. These trends are clearly reflected in quantitative ^1^H NMR analysis of the crude reaction mixtures of 4, 33, and 40 (see SI). For the tertiary radical involved in the synthesis of 4, no cross-coupling product is detected; instead, the Giese-type addition product is formed in a 1.2 : 1.0 ratio relative to the targeted difunctionalized product 4. In contrast, secondary radicals predominantly yield the cross-coupled product, while the Giese-type and desired difunctionalized products are formed in comparable amounts (1.15 : 1.00 for 33). The least stable and least sterically demanding primary radical likewise furnishes the cross-coupled product as the major component, with only trace amounts of the difunctionalized product observed, as exemplified by 40.

Encouraged by the work of Chu and Gutierrez^18^ and other groups^24,47,50,68,102^ we wanted to explore the potential to install the aromatic moiety in an enantioenriched fashion. Consequently, we engaged the synthesis of 22 utilizing a chiral Ni catalyst employing the standard conditions. To our delight 22 was generated in 66% yield with an ee of >94% (Scheme 2). This illustrates that this dicarbofunctuionlization strategy indeed is compatible with enantioselective multicomponent based diversification of electron deficient alkenes. Studies focused on further optimization of the chiral ligand and reaction parameters, as well as expansion of the enantioselective scope are ongoing in our lab.

**Fig. 3:**
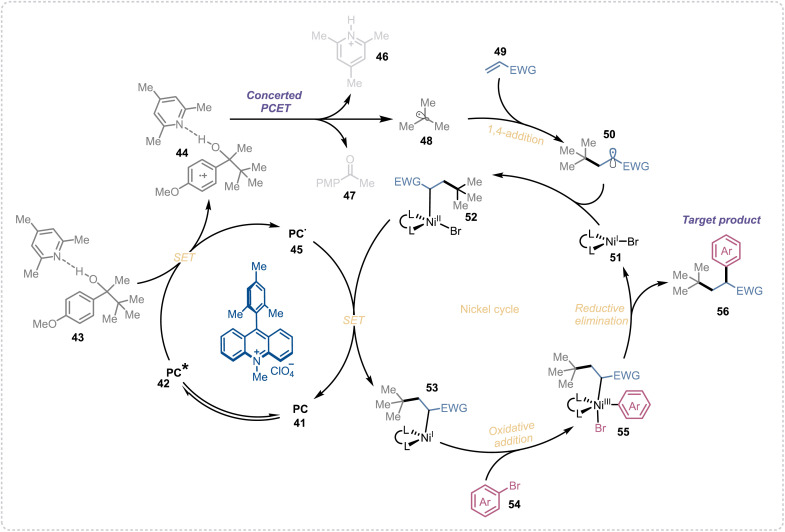
Proposed reaction mechanism.

In our previous investigation of the reaction mechanism, we have concluded the concerted PCET is the main mechanism of alkyl radical generation from these aliphatic alcohols. So based on our previous mechanistic studies^96,97^ and the reported literature on photoredox/nickel dual catalytic cross-coupling reactions,^103–105^ we propose the following plausible mechanism for the alkene dicarbofunctionalization reaction (Fig. 3). Upon visible-light irradiation, the photocatalyst (41) in its excited state (42) oxidizes the base complex substrate (43) to its corresponding oxidized form (44). This oxidized species then undergoes a concerted proton-coupled electron transfer (PCET), generating the key alkyl radical (48) along with a protonated base (46) and a ketone byproduct (47). The alkyl radical subsequently adds to an electron-deficient olefin (49), forming a new radical intermediate (50). This intermediate then combines with a Ni(i)–Br complex (51) to yield a Ni(ii)–alkyl–bromo species (52). Next, a single-electron transfer (SET) from the reduced photocatalyst (45) to the nickel complex (52) regenerates the ground-state photocatalyst and produces a Ni(i)–alkyl species (53). Oxidative addition of the aryl halide (54) to this Ni(i) complex forms a Ni(iii)–alkyl–aryl–bromo intermediate (55), which undergoes reductive elimination to deliver the desired difunctionalized product (56) and a Ni(i)–bromo species (51), thereby completing the nickel catalytic cycle.

## Conclusions

In conclusion, we have developed an efficient PCET mediated photoredox/nickel dual catalytic strategy that achieves three-component alkene dicarbofunctionalization directly from unprotected alcohols. This redox-neutral protocol merges concerted PCET-mediated alkyl radical generation with nickel-catalyzed cross-coupling, enabling the direct use of secondary and tertiary alcohols as alkyl radical precursors under visible light. The method proceeds under mild conditions, displays broad functional-group tolerance, and provides access to diverse vicinal aryl–alkyl disubstituted products. The observed reactivity trend across different alcohol classes correlates with radical stability and underlines the mechanistic synergy between photoredox and nickel catalysis. Product distribution analysis further reveals a radical stability dependent competition between conjugate addition and direct nickel capture, rationalizing diminished efficiency for less stabilized radicals. Moreover, the successful realization of an enantioselective example highlights the potential of this approach for asymmetric multicomponent synthesis. Ongoing efforts are directed toward developing broadly applicable enantioselective variants of this transformation through catalyst and ligand design. This work establishes a general and practical platform for deconstructive difunctionalization of alkenes from readily available alcohols and paves the way toward sustainable, atom-economical radical transformations driven by PCET.

## Author contributions

Y. P.: conceptualization (lead); methodology; investigation; validation; formal analysis; data curation; writing – original draft. V. J.: investigation; validation; formal analysis; data curation. J. Ö.: investigation; validation; formal analysis; data curation. C.-J. W.: conceptualization (lead); supervision; project administration; funding acquisition; writing – review & editing.

## Conflicts of interest

There are no conflicts to declare.

## Supplementary Material

SC-OLF-D6SC00382F-s001

## Data Availability

Supplementary information (SI): all experimental procedures, characterization data, and copies of NMR spectra for new compounds. See DOI: https://doi.org/10.1039/d6sc00382f.
