# Supplementary material for: Photocatalytic multicomponent alkene dicarbofunctionalization via PCET/nickel dual catalysis
Source: Chem Sci. 2026 Feb 19;17(15):7690–8. doi: 10.1039/d6sc00382f (PMC12933641; doi:10.1039/d6sc00382f)

Supplementary Information (SI) for Chemical Science.
This journal is © The Royal Society of Chemistry 2026

Photocatalytic Multicomponent Alkene Dicarbofunctionalization via PCET/Nickel Dual Catalysis

Yeersen Patehebieke,[a] Victoria Jansson,[a] Jakob Öberg,[a] and Carl-Johan Wallentin*[a]

[a] Department of Chemistry and Molecular Biology, University of Gothenburg, Gothenburg SE 41390, Sweden

E-mail: carl.wallentin@chem.gu.se

Table of Contents

General information 3

Photo-redox reaction standard reaction set-up 4

Optimization of reaction conditions 5

General experimental procedure for photoreaction (GP1) 6

General experimental procedure for enantioselective photoreaction (GP2) 6

Preparation and characterization of dicarbofunctionalized products 7

Yield determination of non-isolated products 15

Synthesis of starting materials, ligands and catalysts 16

Reference list 17

NMR spectra of isolated compounds 18

NMR spectra for yield determination of non-isolated products 35

NMR spectra of crude reaction mixtures illustrating mass balance of compounds 4, 33, and 40 45

General information

All reactions were carried out in oven-dried tubes (T_{oven} = 150 °C) by using Schlenk techniques and syringes under nitrogen atmosphere. Commercial grade reagents were purchased from Sigma-Aldrich, Fluorochem and VWR Chemicals and used without further purification, unless otherwise indicated. For thin-layer chromatography (TLC) silica gel coated aluminium plates (60, F_{254}, Merck) were employed and analyzed with UV light at 254 or 365 nm and by means of staining with anisaldehyde or Vanillin stains. The purification of products was performed on silica gel 60 M (40 – 63 μm, VWR Chemicals) by using the flash technique under a pressure of 2 bar or with Büchi Pure C-810 Flash (using Büchi FlashPure EcoFlex cartridges of either 50 μm or 20 μm).

1,2-dichloroethane (DCE) and acetonitrile (ACN) used for photoredox reactions were filtered through basic Al_2O_3 and degassed prior to use. Other common dry solvents were obtained from a solvent purification system or purchased from Sigma-Aldrich and used as supplied without further purification, unless otherwise indicated.

GC-MS (EI) was performed on Agilent Technologies 7820A gas chromatograph with an Agilent 5977E MSD detector. Separations were performed on Agilent 19091S-433 J&W HP-5ms GC Column, 30 m, 0.25 mm, 0.25 μm, 7-inch cage.

HRMS data were recorded with a QExactive HF Orbitrap mass spectrometer interfaced with Dionex Ultimate 3000 liquid chromatography system (Thermo Fisher Scientific). The instrument operated in full MS mode only, where the ion mass spectra were acquired at a resolution of 120 000, maximum injection time 200 ms for 3e6 ions. The Orbitrap was calibrated with Pierce LTQ ESI Positive Ion Calibration Solution prior to the analysis, resulting in mass accuracy better than 5 ppm. Electrospray ionization were performed at 4 kV and 320 degrees Celsius using a metal emitter in the ion source. The sample (10 microliter) was injected onto a reversed-phase XBridge BEH C18 column (3.5 μm, 2.1x50 mm, Waters Corporation). The analysis was performed using a linear gradient from 10% solvent B (80% acetonitrile in water and 0.1% formic acid) to 100% solvent B over 2.5 min followed by 100% during 17.5 min with flow of 0.300 ml/min and solvent A being 0.1% formic acid in water.

^{1}H-NMR, ^{13}C-NMR and ^{19}F-NMR spectra were recorded on a Varian NMR 400 or Bruker Avance III HD 600 MHz spectrometer. Chemical shifts (δ) are reported in ppm relative to the residual solvent peak, and solvent signal was used as the reference for ^{1}H NMR ($CDCl_3$, 7.26 ppm) and ^{13}C NMR ($CDCl_3$, 77.16 ppm). Splitting patterns are indicated as (s) singlet, (d) doublet, (dd) doublet of doublets, (t) triplet, (tt) triplet of triplets, (dt) doublet of triplets, (td) triplet of doublets, (q) quartet, (quint) quintet, (sext) sextet, (hept) heptet, (m) multiplet. Coupling constants (*J*) are reported in Hertz (Hz).

Photo-redox reaction standard reaction set-up

Photo-redox reactions were performed in a Biotage® Microwave reactions Vials using Lucent360™ Advanced Photoreactor. 450 nm Light with 75% intensity. The reactions temperature maintained at 35 °C by Grant TC120 series of digital heating circulating bath connected to Lucent 360 photo reactor.

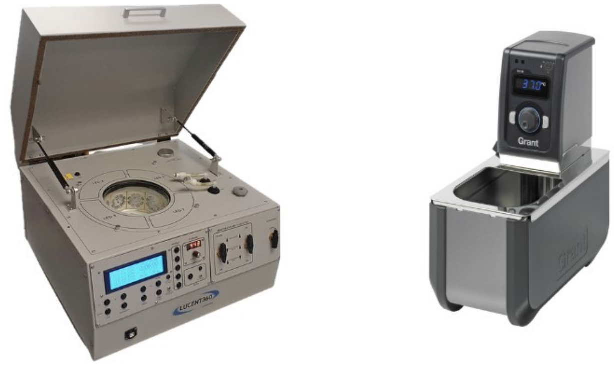

Figure S1. Photo reaction set-up

Optimization of reaction conditions

Table S1. Optimization of reaction conditions[a]

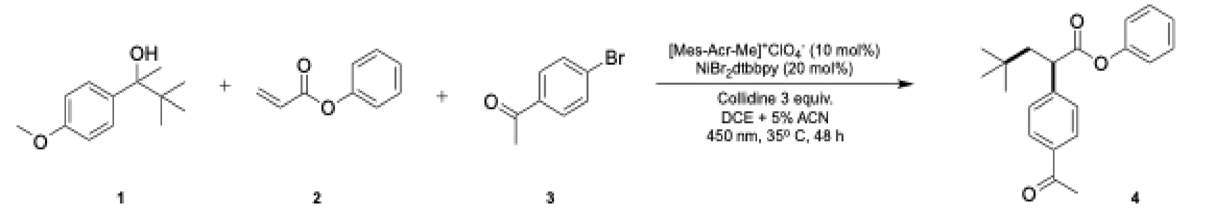

Entry	Deviation from standard conditions	Yield[b] (%)
1	No deviation	82
2	ACN	n.d.
3	DCM	57
4	DCE	73
5	DMF	trace
6	Stoichiometry[c]	57
7	Stoichiometry[d]	39
8	Stoichiometry[e]	49
9	$[Mes\text{-}Acr\text{-}Me]^+BF_4^-$	81
10	$[Mes\text{-}Acr\text{-}Ph]^+BF_4^-$	63
11	$[Mes\text{-}Acr\text{-}tBu]^+BF_4^-$	13
12	100% light intensity	77
13	6 h, 100% light intensity	76
14	12 h, 100% light intensity	76
15	24 h, 100% light intensity	76
16	6 h	75
17	12 h	82
18	6 h, 75% light intensity	78
19	***12 h, 75% light intensity***	***84***
20	No nickel catalyst	n.d.
21	No photocatalyst	n.d.
22	No base	n.d.
23	No light	n.d.

[a] Reaction conditions: alcohol (0.3 mmol), alkene (0.2 mmol), aryl bromide (0.1 mmol), 2,4,6-collidine (0.3 mmol), $[Mes\text{-}Acr\text{-}Me]^+ClO_4^-$ (0.01 mmol), $NiBr_2$dtbbpy (0.02 mmol), DCE+5% ACN (3 mL), time (48 h), temperature (35 °C), photo reactor (Lucent 360, 450 nm, 50% light intensity). [b] Yields determined by ^{1}H NMR analysis of crude with ethylene carbonate as internal standard. [c] alcohol (0.15 mmol), alkene (0.15 mmol), aryl bromide (0.1 mmol) [d] alcohol (0.1 mmol), alkene (0.1 mmol), aryl bromide (0.1 mmol) [e] alcohol (0.2 mmol), alkene (0.15 mmol), aryl bromide (0.1 mmol)

General experimental procedure for photoreaction (GP1)

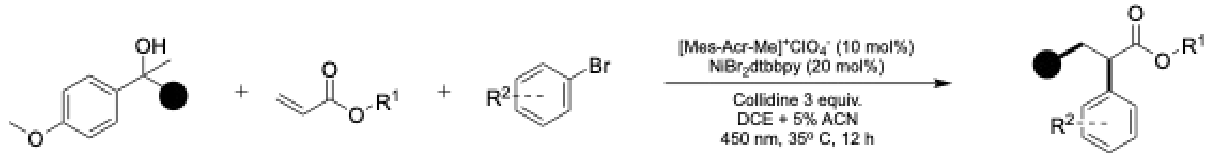

Anhydrous 1,2-Dichloroethane (DCE) and acetonitrile (ACN) were mixed in a ratio of 20:1 and deoxygenated by sparging with N_2 for at least 30 min. Fukuzumi catalyst (4.2 mg, 0.01mmol, 10 mol%), alcohol (0.3 mmol, 3.0 equiv.), $NiBr_2$•dtbbpy (9.7 mg, 0.02 mmol, 20 mol%), (if solid) the aryl halide (0.1 mmol, 1.0 equiv.) and a stir bar were added to an oven dried Biotage MW vial (2.0–5.0 mL). The microwave vial was then capped tightly, and vacuum purged 3 times with N_2 gas. After filling with N_2 the third time: solvent (3 mL), (if liquid) aryl halide (0.1 mmol, 1.0 equiv.), acrylate (0.2 mmol, 2.0 equiv.), and collidine (0.3 mmol, 3.0 equiv.) were added by syringe. The reaction head space was then sparged for 5 min. The capped end of the vial was wrapped with parafilm and placed in Lucent 360 photoreactor set up (as illustrated in Figure S1) then stirred under direct blue LED irradiation at 450 nm for 12 hours. The reaction temperature was maintained at 35 ºC using a Grant TC120 series of digital heating circulating bath connected to the Lucent 360 photoreactor. The crude was filtered through a pad of silica using DCM, concentrated under reduced pressure and purified by flash column chromatography using Petroleum spirit and 1% EtOAc as eluent mixture to afford the product. Compounds on TLC plates were visualized with UV (254 nm), p-anisaldehyde or vanillin stains.

General experimental procedure for enantioselective photoreaction (GP2)

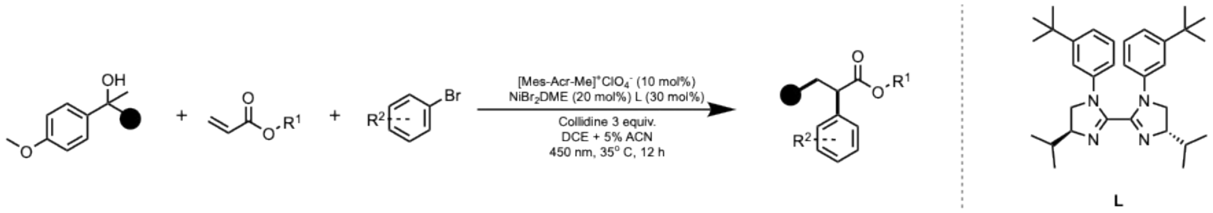

Anhydrous 1,2-Dichloroethane (DCE) and acetonitrile (ACN) were mixed in a ratio of 20:1 and deoxygenated by sparging with N_2 for at least 30 min. $NiBr_2$•DME (6.17 mg, 0.02 mmol, 20 mol%), ligand **L** (14.6 mg, 0.03 mmol, 30 mol%), and a stir bar were added to an oven dried Biotage MW vial (2.0–5.0 mL). The MW vial was then capped tightly, and vacuum purged 3 times with N_2 gas. After filling with N_2 the third time: solvent (1 mL) was added and the mixture was stirred for 30 min. Fukuzumi catalyst (4.2 mg, 0.01mmol, 10 mol%), alcohol (0.3 mmol, 3.0 equiv.), aryl halide (0.1 mmol, 1.0 equiv.), acrylate (0.2 mmol, 2.0 equiv.), and collidine (0.3 mmol, 3.0 equiv.) were added and the MW vial was recapped and the mixture was then deoxygenated by sparging with N_2 for 30 min. The capped end of the vial was wrapped with parafilm and placed in Lucent 360 photoreactor set up (as illustrated in Figure S1) then stirred under direct blue LED irradiation at 450 nm for 12 hours. The reaction temperature was maintained at 35 ºC with a Grant TC120 series of digital heating circulating bath connected to the Lucent 360 photo reactor. The crude was filtered through a pad of silica using DCM, concentrated under reduced pressure and purified by flash column chromatography using Petroleum spirit and 1% EtOAc as eluent mixture to afford the product.

Preparation and characterization of dicarbofunctionalized products

***tert*-Butyl 2-(4-cyanophenyl)-4,4-dimethylpentanoate (5)**

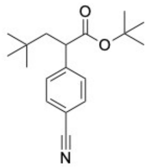

Compound (**5**) was synthesized following GP1 using alcohol 1 (62.5 mg, 0.3 mmol), 4-bromobenzonitrile (18.2 mg, 0.1 mmol), tert-butyl acrylate (29.3 µL, 0.2 mmol), $NiBr_2$•dtbbpy (9.7 mg, 0.02 mmol), [Mes-Acr-Me]$^+ClO_4^-$ (4.2 mg 0.01 mmol), and 2,4,6-collidine (40 µL, 0.30 mmol). After 12 h, the reaction produced the desired compound as a white solid (22.8 mg, **77%** yield) after flash chromatography (petroleum spirit/EtOAc 99:1). Spectroscopic data agrees with the literature.[1]

^{1}H NMR (600 MHz, $CDCl_3$) δ 7.59 (d, *J* = 8.4 Hz, 2H), 7.41 (d, *J* = 8.4 Hz, 2H), 3.57 (dd, *J* = 9.0, 3.7 Hz, 1H), 2.26 (dd, *J* = 14.0, 9.0 Hz, 1H), 1.47 (dd, *J* = 14.0, 3.8 Hz, 1H), 1.37 (s, 9H), 0.90 (s, 9H).

^{13}C NMR (151 MHz, $CDCl_3$) δ 172.7, 147.0, 132.3, 128.6, 118.8, 110.7, 81.2, 49.5, 47.0, 31.1, 29.5, 27.8.

HRMS (ESI) m/z: exact mass calculated for $C_{18}H_{26}NO_2$ [$(M+H)^+$]: 288.1958, Found: 288.19531

***tert*-Butyl 4,4-dimethyl-2-(4-(methylsulfonyl)phenyl)pentanoate (6)**

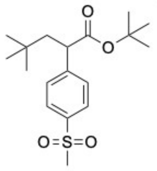

Compound (**6**) was synthesized following GP1 using alcohol 1 (62.5 mg, 0.3 mmol), 1-bromo-4-(methylsulfonyl)benzene (23.5 mg 0.1 mmol), tert-butyl acrylate (29.3 µL, 0.2 mmol), $NiBr_2$•dtbbpy (9.7 mg, 0.02 mmol), [Mes-Acr-Me]$^+ClO_4^-$ (4.2 mg 0.01 mmol), and 2,4,6-collidine (40 µL, 0.30 mmol). After 12 h, the reaction produced the desired compound as a white solid (27.2 mg, **79%** yield) after flash chromatography (petroleum spirit/EtOAc 99:1). Spectroscopic data agrees with the literature.[2]

^{1}H NMR (600 MHz, $CDCl_3$) δ 7.87 (d, *J* = 8.4 Hz, 2H), 7.51 (d, *J* = 8.3 Hz, 2H), 3.62 (dd, *J* = 9.1, 3.6 Hz, 1H), 3.05 (s, 3H), 2.28 (dd, *J* = 14.0, 9.1 Hz, 1H), 1.48 (dd, *J* = 14.0, 3.6 Hz, 1H), 1.38 (s, 9H), 0.91 (s, 9H).

^{13}C NMR (151 MHz, $CDCl_3$) δ 172.9, 148.1, 139.1, 128.9, 127.8, 81.4, 49.5, 47.4, 44.7, 31.3, 29.6, 28.0.

HRMS (ESI) m/z: exact mass calculated for $C_{13}H_{19}O_2S$ [$(M-C_5H_{10}O_2)^-$]: 239.1111, Found: 239.11098

***tert*-Butyl 4,4-dimethyl-2-(4-(trifluoromethyl)phenyl)pentanoate (7)**

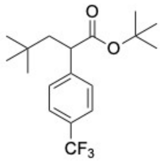

Compound (**7**) was synthesized following GP1 using alcohol 1 (62.5 mg, 0.3 mmol), 1-bromo-4-(trifluoromethyl)benzene (22.5 mg, 0.1 mmol), tert-butyl acrylate (29.3 μL, 0.2 mmol), $NiBr_2$•dtbbpy (9.7 mg, 0.02 mmol), $[Mes\text{-}Acr\text{-}Me]^+ClO_4^-$ (4.2 mg 0.01 mmol), and 2,4,6-collidine (40 μL, 0.30 mmol). After 12 h, the reaction produced the desired compound as a white solid (24.1 mg, **73%** yield) after flash chromatography (petroleum spirit/EtOAc 99:1). Spectroscopic data agrees with the literature.[2]

^{1}H NMR (600 MHz, $CDCl_3$) δ 7.55 (d, *J* = 7.7 Hz, 2H), 7.42 (d, *J* = 8.0 Hz, 2H), 3.59 (dd, *J* = 9.1, 3.6 Hz, 1H), 2.28 (dd, *J* = 14.0, 9.2 Hz, 1H), 1.48 (dd, *J* = 14.0, 3.6 Hz, 1H), 1.37 (s, 9H), 0.91 (s, 9H).

^{13}C NMR (151 MHz, $CDCl_3$) δ 173.3, 145.8, 129.2 (q, *J* = 32.7 Hz), 128.2, 125.6 (d, *J* = 4.4 Hz), 124.4 (q, *J* = 271.8 Hz), 81.0, 49.4, 47.3, 31.3, 29.6, 28.0.

^{19}F NMR (564 MHz, $CDCl_3$) δ -62.33 (s)

HRMS (ESI) m/z: exact mass calculated for $C_{14}H_{16}F_3O_2$ $[(M\text{-}C(CH_3)_3)^-]$: 273.1108, Found: 273.11099

***tert*-Butyl 2-(4-acetylphenyl)-4,4-dimethylpentanoate (8)**

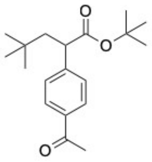

Compound (**8**) was synthesized following GP1 using alcohol 1 (62.5 mg, 0.3 mmol), 1-(4-bromophenyl)ethan-1-one (19.9 mg, 0.1 mmol), tert-butyl acrylate (29.3 μL, 0.2 mmol), $NiBr_2$•dtbbpy (9.7 mg, 0.02 mmol), $[Mes\text{-}Acr\text{-}Me]^+ClO_4^-$ (4.2 mg 0.01 mmol), and 2,4,6-collidine (40 μL, 0.30 mmol). After 12 h, the reaction produced the desired compound as a white solid (22.2 mg, **71%** yield) after flash chromatography (petroleum spirit/EtOAc 99:1). Spectroscopic data agrees with the literature.[2]

^{1}H NMR (600 MHz, $CDCl_3$) δ 7.89 (d, *J* = 8.4 Hz, 2H), 7.40 (d, *J* = 8.3 Hz, 2H), 3.59 (dd, *J* = 9.0, 3.7 Hz, 1H), 2.59 (s, 3H), 2.28 (dd, *J* = 14.0, 9.0 Hz, 1H), 1.50 (dd, *J* = 14.0, 3.8 Hz, 1H), 1.37 (s, 9H), 0.91 (s, 9H).

^{13}C NMR (151 MHz, $CDCl_3$) δ 198.1, 173.5, 147.4, 136.1, 129.0, 128.3, 81.2, 49.8, 47.4, 31.5, 29.8, 28.2, 26.9.

HRMS (ESI) m/z: exact mass calculated for $C_{19}H_{29}O_3$ $[(M+H)^+]$: 305.2111, Found: 305.2106

Methyl 4-(1-(*tert*-butoxy)-4,4-dimethyl-1-oxopentan-2-yl)benzoate (9)

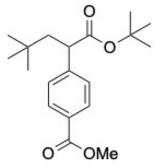

Compound (**9**) was synthesized following GP1 using alcohol 1 (62.5 mg, 0.3 mmol), methyl 4-bromobenzoate (21.5 mg, 0.1 mmol), tert-butyl acrylate (29.3 µL, 0.2 mmol), $NiBr_2$•dtbbpy (9.7 mg, 0.02 mmol), $[Mes\text{-}Acr\text{-}Me]^+ClO_4^-$ (4.2 mg 0.01 mmol), and 2,4,6-collidine (40 µL, 0.30 mmol). After 12 h, the reaction produced the desired compound as a white solid (20.8 mg, **64%** yield) after flash chromatography (petroleum spirit/EtOAc 99:1). Spectroscopic data agrees with the literature.[2]

^{1}**H NMR** (600 MHz, $CDCl_3$) δ 7.96 (d, *J* = 8.4 Hz, 2H), 7.37 (d, *J* = 8.3 Hz, 2H), 3.90 (s, 3H), 3.58 (dd, *J* = 9.0, 3.8 Hz, 1H), 2.27 (dd, *J* = 14.0, 9.0 Hz, 1H), 1.50 (dd, *J* = 14.0, 3.8 Hz, 1H), 1.36 (s, 9H), 0.90 (s, 9H).

^{13}**C NMR** (151 MHz, $CDCl_3$) δ 173.3, 167.1, 147.0, 130.0, 128.8, 127.9, 80.9, 52.2, 49.6, 47.1, 31.2, 29.6, 27.9.

HRMS (ESI) m/z: exact mass calculated for $C_{14}H_{19}O_2$ [$(M\text{-}C_5H_{10}O_2)^-$]: 219.1391, Found: 219.13867

***tert*-Butyl 4,4-dimethyl-2-(4-(morpholinosulfonyl)phenyl)pentanoate (10)**

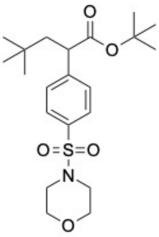

Compound (**10**) was synthesized following GP1 using alcohol 1 (62.5 mg, 0.3 mmol), 4-((4-bromophenyl)sulfonyl)morpholine (30.6 mg, 0.1 mmol), tert-butyl acrylate (29.3 µL, 0.2 mmol), $NiBr_2$•dtbbpy (9.7 mg, 0.02 mmol), $[Mes\text{-}Acr\text{-}Me]^+ClO_4^-$ (4.2 mg 0.01 mmol), and 2,4,6-collidine (40 µL, 0.30 mmol). After 12 h, the reaction produced the desired compound as a white solid (38.5 mg, **92%** yield) after flash chromatography (petroleum spirit/EtOAc 99:1).

^{1}**H NMR** (600 MHz, $CDCl_3$) δ 7.67 (d, *J* = 8.4 Hz, 2H), 7.48 (d, *J* = 8.4 Hz, 2H), 3.76 – 3.71 (m, 4H), 3.61 (dd, *J* = 9.1, 3.6 Hz, 1H), 3.03 – 2.92 (m, 4H), 2.27 (dd, *J* = 14.0, 9.1 Hz, 1H), 1.48 (dd, *J* = 14.0, 3.7 Hz, 1H), 1.37 (s, 9H), 0.91 (s, 9H).

^{13}**C NMR** (151 MHz, $CDCl_3$) δ 173.1, 147.6, 133.8, 128.8, 128.5, 81.5, 66.4, 49.7, 47.5, 46.3, 31.5, 29.8, 28.2.

HRMS (ESI) m/z: exact mass calculated for $C_{21}H_{34}NO_5S$ [$(M+H)^+$]: 412.2152, Found: 412.21436

***tert*-Butyl 2-([1,1'-biphenyl]-4-yl)-4,4-dimethylpentanoate (19)**

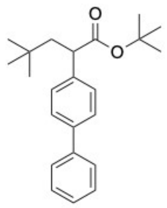

Compound (**19**) was synthesized following GP1 using alcohol 1 (62.5 mg, 0.3 mmol), 4-bromo-1,1'-biphenyl (23.3 mg, 0.1 mmol), tert-butyl acrylate (29.3 μL, 0.2 mmol), $NiBr_2$•dtbbpy (9.7 mg, 0.02 mmol), $[Mes\text{-}Acr\text{-}Me]^+ClO_4^-$ (4.2 mg 0.01 mmol), and 2,4,6-collidine (40 μL, 0.30 mmol). After 12 h, the reaction produced the desired compound as a white solid (17.6 mg, **51%** yield) after flash chromatography (petroleum spirit/EtOAc 99:1). Spectroscopic data agrees with the literature.[3]

^{1}H NMR (600 MHz, $CDCl_3$) δ 7.59 (d, *J* = 7.0 Hz, 2H), 7.53 (d, *J* = 8.3 Hz, 2H), 7.43 (t, *J* = 7.7 Hz, 2H), 7.37 (d, *J* = 8.2 Hz, 2H), 7.33 (t, *J* = 7.4 Hz, 1H), 3.58 (dd, *J* = 9.4, 3.4 Hz, 1H), 2.31 (dd, *J* = 14.0, 9.4 Hz, 1H), 1.52 (dd, *J* = 13.8, 3.4 Hz, 1H), 1.40 (s, 9H), 0.94 (s, 9H).

^{13}C NMR (151 MHz, $CDCl_3$) δ 173.9, 140.9, 140.8, 139.5, 128.7, 128.1, 127.2, 127.0, 80.4, 49.0, 47.3, 31.1, 29.5, 27.9.

HRMS (ESI) m/z: exact mass calculated for $C_{18}H_{21}$ $[(M\text{-}C_5H_{10}O_2)^-]$: 237.1649, Found: 237.16469

***tert*-Butyl 4,4-dimethyl-2-(naphthalen-2-yl)pentanoate (20)**

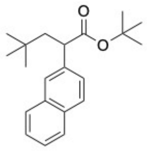

Compound (**20**) was synthesized following GP1 using alcohol 1 (62.5 mg, 0.3 mmol), 2-bromonaphthalene (20.7 mg, 0.1 mmol), tert-butyl acrylate (29.3 μL, 0.2 mmol), $NiBr_2$•dtbbpy (9.7 mg, 0.02 mmol), $[Mes\text{-}Acr\text{-}Me]^+ClO_4^-$ (4.2 mg 0.01 mmol), and 2,4,6-collidine (40 μL, 0.30 mmol). After 12 h, the reaction produced the desired compound as a white solid (16.1 mg, **50%** yield) after flash chromatography (petroleum spirit/EtOAc 99:1). Spectroscopic data agrees with the literature.[4]

^{1}H NMR (600 MHz, $CDCl_3$) δ 7.83 – 7.76 (m, 3H), 7.73 (s, 1H), 7.45 (dddd, *J* = 14.5, 8.4, 6.7, 1.6 Hz, 3H), 3.70 (dd, *J* = 9.2, 3.5 Hz, 1H), 2.36 (dd, *J* = 14.0, 9.2 Hz, 1H), 1.60 (dd, *J* = 14.0, 3.6 Hz, 1H), 1.37 (s, 9H), 0.94 (s, 9H).

^{13}C NMR (151 MHz, $CDCl_3$) δ 174.0, 139.3, 133.7, 132.6, 128.2, 127.9, 127.7, 126.3, 126.3, 126.1, 125.7, 80.6, 49.6, 47.4, 31.3, 29.7, 28.0.

HRMS (ESI) m/z: exact mass calculated for $C_{16}H_{19}$ $[(M\text{-}C_5H_{10}O_2)^-]$: 211.1492, Found: 211.14839

***tert*-Butyl 2-(2-fluoropyridin-4-yl)-4,4-dimethylpentanoate (22)**

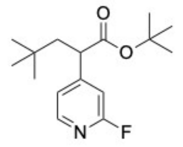

Compound (**22**) was synthesized following GP1 using alcohol 1 (62.5 mg, 0.3 mmol), 4-bromo-2-fluoropyridine (17.6 mg, 0.1 mmol), tert-butyl acrylate (29.3 µL, 0.2 mmol), $NiBr_2$•dtbbpy (9.7 mg, 0.02 mmol), [Mes-Acr-Me]$^+ClO_4^-$ (4.2 mg 0.01 mmol), and 2,4,6-collidine (40 µL, 0.30 mmol). After 12 h, the reaction produced the desired compound as a white solid (21.8 mg, **76%** yield) after flash chromatography (petroleum spirit/EtOAc 99:1). Spectroscopic data agrees with the literature.[2]

Enantioenriched product was synthesized following GP2 using alcohol 1 (62.5 mg, 0.3 mmol), 4-bromo-2-fluoropyridine (17.6 mg, 0.1 mmol), tert-butyl acrylate (29.3 µL, 0.2 mmol), $NiBr_2$•DME (6.17 mg, 0.02 mmol), L (14.6 mg, 0.03 mmol,)[Mes-Acr-Me]$^+ClO_4^-$ (4.2 mg 0.01 mmol), and 2,4,6-collidine (40 µL, 0.30 mmol). After 12 h, (**22**) was provided in **66%** yield and in **94%** ee.

^{1}H NMR (600 MHz, $CDCl_3$) δ 8.13 (d, J = 5.2 Hz, 1H), 7.12 (dt, J = 5.3, 1.7 Hz, 1H), 6.87 (s, 1H), 3.55 (dd, J = 9.1, 3.6 Hz, 1H), 2.25 (dd, J = 14.0, 9.1 Hz, 1H), 1.46 (dd, J = 14.0, 3.6 Hz, 1H), 1.39 (s, 9H), 0.91 (s, 9H).

^{13}C NMR (151 MHz, $CDCl_3$) δ 171.9, 164.0 (d, J = 238.7 Hz), 156.2 (d, J = 8.0 Hz), 147.7 (d, J = 15.2 Hz), 120.7 (d, J = 4.0 Hz), 108.5 (d, J = 37.7 Hz), 81.5, 48.9, 46.7, 31.1, 29.4, 27.8.

^{19}F NMR (564 MHz, $CDCl_3$) δ -68.08 (s)

HRMS (ESI) m/z: exact mass calculated for $C_{16}H_{25}FNO_2$ [$(M+H)^+$]: 282.1864, Found: 282.18585

Chiral HPLC: Chiralpak IC, 40 °C, 20 mM NH_3 in ^{i}PrOH, 3.5 mL/min, 254 nm, t_R (major)= 1.63 min, t_R (minor)= 2.40 min.

22 racemic

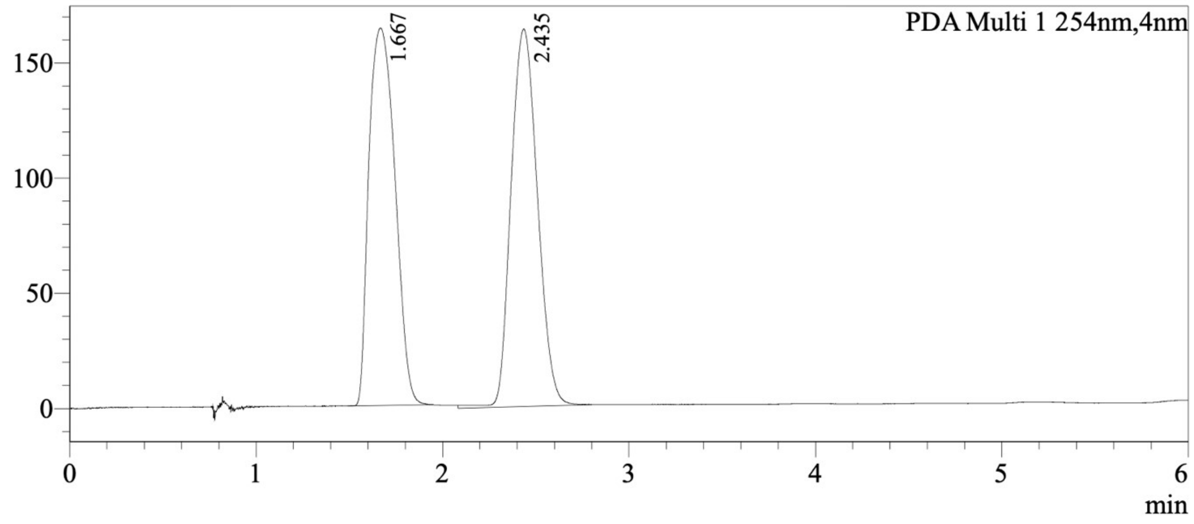

Peak#	Ret. Time	Height	Area	Area%
1	1.667	163575	1596055	49.645

2	2.435	163621	1618886	50.355

22 enantioenriched, 94% ee

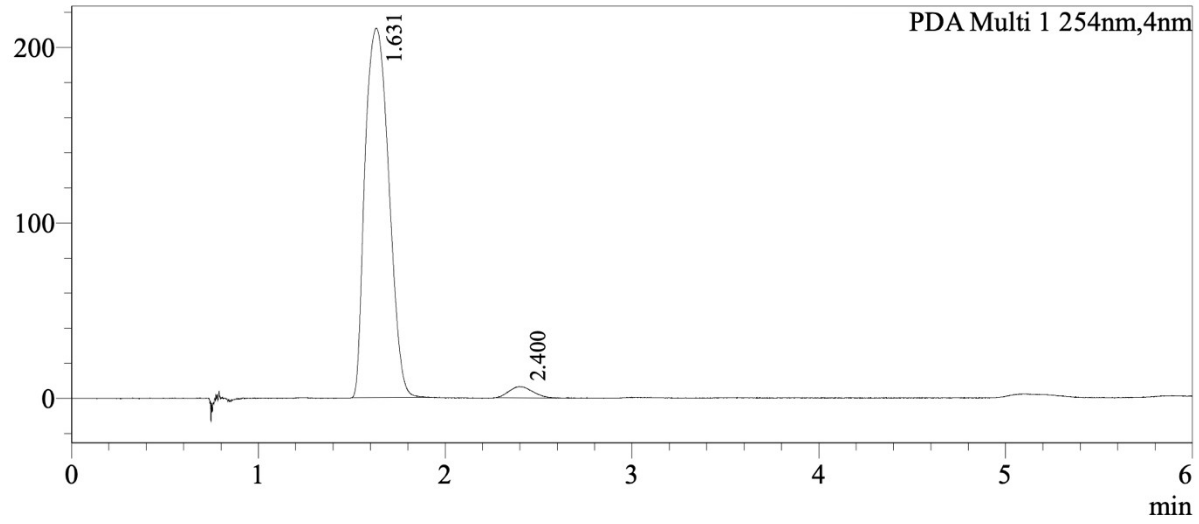

Peak#	Ret. Time	Height	Area	Area%
1	1.631	210509	1865495	97.150
2	2.400	6224	54720	2.850

***tert*-Butyl 4,4-dimethyl-2-(2-methylquinolin-6-yl)pentanoate (23)**

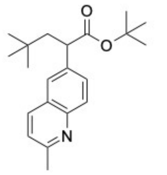

Compound (**23**) was synthesized following GP1 using alcohol 1 (62.5 mg, 0.3 mmol), 6-bromo-2-methylquinoline (22.2 mg, 0.1 mmol), tert-butyl acrylate (29.3 μL, 0.2 mmol), $NiBr_2$•dtbbpy (9.7 mg, 0.02 mmol), $[Mes\text{-}Acr\text{-}Me]^+ClO_4^-$ (4.2 mg 0.01 mmol), and 2,4,6-collidine (40 μL, 0.30 mmol After 12 h, the reaction produced the desired compound as a white solid (19.7 mg, **60%** yield) after flash chromatography (petroleum spirit/EtOAc 99:1).

^{1}H NMR (600 MHz, $CDCl_3$) δ 8.00 (d, J = 7.6 Hz, 1H), 7.95 (d, J = 8.5 Hz, 1H), 7.69 – 7.62 (m, 2H), 7.27 (d, J = 7.3 Hz, 1H), 3.70 (dd, J = 9.1, 3.7 Hz, 1H), 2.73 (s, 3H), 2.35 (dd, J = 14.0, 9.1 Hz, 1H), 1.61 – 1.60 (m, 1H), 1.37 (s, 9H), 0.93 (s, 9H).

^{13}C NMR (151 MHz, $CDCl_3$) δ 174.0, 159.1, 147.4, 139.4, 136.4, 130.2, 129.1, 126.8, 126.0, 122.5, 81.0, 49.5, 47.5, 31.5, 29.9, 28.2, 25.7.

HRMS (ESI) m/z: exact mass calculated for $C_{21}H_{30}NO_2$ [$(M+H)^+$]: 328.2271, Found: 328.22623

***tert*-Butyl 3-cyclohexyl-2-(2-fluoropyridin-4-yl)propanoate (33)**

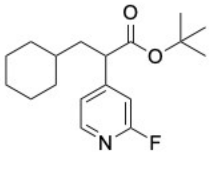

Compound (**33**) was synthesized following GP1 using 1-cyclohexyl-1-(4-methoxyphenyl)ethan-1-ol (70.3 mg, 0.3 mmol), 4-bromo-2-fluoropyridine (17.6 mg, 0.1 mmol), tert-butyl acrylate (29.3 μL, 0.2 mmol), $NiBr_2$•dtbbpy (9.7 mg, 0.02 mmol), [Mes-Acr-Me]$^+ClO_4^-$ (4.2 mg 0.01 mmol), and 2,4,6-collidine (40 μL, 0.30 mmol). After 12 h, the reaction produced the desired compound as a white solid (10.8 mg, 35% yield) after flash chromatography (petroleum spirit/EtOAc 99:1). Spectroscopic data agrees with the literature.[5]

^{1}H NMR (600 MHz, $CDCl_3$) δ 8.14 (d, *J* = 5.2 Hz, 1H), 7.12 (dt, *J* = 5.2, 1.7 Hz, 1H), 6.88 (s, 1H), 3.63 – 3.58 (m, 1H), 1.96 – 1.90 (m, 1H), 1.74 – 1.67 (m, 4H), 1.60 – 1.54 (m, 2H), 1.40 (s, 9H), 1.18 – 1.12 (m, 4H), 0.95 – 0.87 (m, 2 H)

^{13}C NMR (151 MHz, $CDCl_3$) δ 171.7, 164.2 (d, *J* = 238.7 Hz), 155.0 (d, *J* = 7.7 Hz), 147.7 (d, *J* = 15.2 Hz), 121.1 (d, *J* = 4.0 Hz), 109.0 (d, *J* = 37.6 Hz), 81.7, 49.6, 40.8, 35.6, 33.3, 33.2, 28.3, 28.0, 26.5, 26.2.

^{19}F NMR (564 MHz, $CDCl_3$) δ -68.18 (s)

HRMS (ESI) m/z: exact mass calculated for $C_{18}H_{27}FNO_2$ [$(M+H)^+$]: 308.2020, Found: 308.20108

***tert*-Butyl 3-cyclopentyl-2-(2-fluoropyridin-4-yl)propanoate (34)**

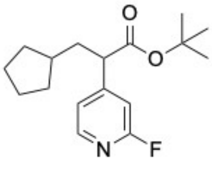

Compound (**34**) was synthesized following GP1 using 1-cyclopentyl-1-(4-methoxyphenyl)ethan-1-ol (66.1 mg, 0.3 mmol), 4-bromo-2-fluoropyridine (17.6 mg, 0.1 mmol), tert-butyl acrylate (29.3 μL, 0.2 mmol), $NiBr_2$•dtbbpy (9.7 mg, 0.02 mmol), [Mes-Acr-Me]$^+ClO_4^-$ (4.2 mg 0.01 mmol), and 2,4,6-collidine (40 μL, 0.30 mmol). After 12 h, the reaction produced the desired compound as a white solid (9.47 mg, **32%** yield) after flash chromatography (petroleum spirit/EtOAc 99:1).

^{1}H NMR (600 MHz, $CDCl_3$) δ 8.14 (d, *J* = 5.2 Hz, 1H), 7.13 (dt, *J* = 5.3, 1.7 Hz, 1H), 6.89 (s, 1H), 3.52 (t, *J* = 7.7 Hz, 1H), 2.07 – 2.01 (m, 1H), 1.77 – 1.71 (m, 3H), 1.66 – 1.60 (m, 3H), 1.51 – 1.47 (m, 2H), 1.41 (s, 9H), 1.15 – 1.07 (m, 2H).

^{13}C NMR (151 MHz, $CDCl_3$) δ 171.7, 164.2 (d, *J* = 238.6 Hz), 154.8 (d, *J* = 7.8 Hz), 147.7 (d, *J* = 15.2 Hz), 121.1 (d, *J* = 3.9 Hz), 109.0 (d, *J* = 37.6 Hz), 81.7, 51.6, 39.6, 38.0, 32.8, 32.5, 28.0, 25.2, 25.2.

^{19}F NMR (564 MHz, $CDCl_3$) δ -68.21 (s)

HRMS (ESI) m/z: exact mass calculated for $C_{17}H_{25}FNO_2$ [$(M+H)^+$]: 294.1864, Found: 294.18555

tert-Butyl 2-(2-fluoropyridin-4-yl)-4-methylpentanoate (37)

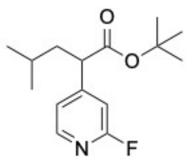

Compound (**37**) was synthesized following GP1 using 1-(4-methoxyphenyl)-2-methylpropan-1-ol (54.1 mg, 0.3 mmol), 4-bromo-2-fluoropyridine (17.6 mg, 0.1 mmol), tert-butyl acrylate (29.3 μL, 0.2 mmol), $NiBr_2$•dtbbpy (9.7 mg, 0.02 mmol), [Mes-Acr-Me]$^+ClO_4^-$ (4.2 mg 0.01 mmol), and 2,4,6-collidine (40 μL, 0.30 mmol). After 12 h, the reaction produced the desired compound as a white solid (8.27 mg, **31%** yield) after flash chromatography (petroleum spirit/EtOAc 99:1).

^{1}H NMR (600 MHz, $CDCl_3$) δ 8.14 (d, *J* = 5.2 Hz, 1H), 7.13 (d, *J* = 5.2 Hz, 1H), 6.88 (s, 1H), 3.59 – 3.54 (m, 1H), 1.93 (ddd, *J* = 13.5, 8.4, 7.1 Hz, 1H), 1.59 (t, *J* = 7.0 Hz, 1H), 1.50 – 1.46 (m, 1H), 1.41 (s, 9H), 0.92 (dd, *J* = 6.6, 4.1 Hz, 6H).

^{13}C NMR (151 MHz, $CDCl_3$) δ 171.6, 164.2 (d, *J* = 238.6 Hz), 154.9 (d, *J* = 8.0 Hz), 147.7 (d, *J* = 15.2 Hz), 121.1 (d, *J* = 4.1 Hz), 109.0 (d, *J* = 37.6 Hz), 81.8, 50.3, 42.2, 28.0, 26.1, 22.5, 22.5.

^{19}F NMR (564 MHz, $CDCl_3$) δ -68.17 (s)

HRMS (ESI) m/z: exact mass calculated for $C_{15}H_{23}FNO_2$ [$(M+H)^+$]: 268.1707, Found: 268.17062

tert-Butyl 4-ethyl-2-(2-fluoropyridin-4-yl)heptanoate (38)

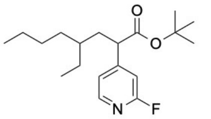

Compound (**38**) was synthesized following GP1 using 2-ethyl-1-(4-methoxyphenyl)pentan-1-ol (66.7 mg, 0.3 mmol), 4-bromo-2-fluoropyridine (17.6 mg, 0.1 mmol), tert-butyl acrylate (29.3 μL, 0.2 mmol), $NiBr_2$•dtbbpy (9.7 mg, 0.02 mmol), [Mes-Acr-Me]$^+ClO_4^-$ (4.2 mg 0.01 mmol), and 2,4,6-collidine (40 μL, 0.30 mmol). After 12 h, the reaction produced the desired compound as a white solid (11.7 mg, **36%** yield) after flash chromatography (petroleum spirit/EtOAc 99:1).

^{1}H NMR (600 MHz, $CDCl_3$) δ 8.15 (d, *J* = 5.2 Hz, 1H), 7.13 (d, *J* = 5.2 Hz, 1H), 6.89 (s, 1H), 3.57 (dd, *J* = 8.6, 6.9 Hz, 1H), 2.03 – 1.95 (m, 1H), 1.62 – 1.58 (m, 1H), 1.41 (s, 9H), 1.34 – 1.17 (m, 9H), 0.91 – 0.81 (m, 6H).

^{13}C NMR (151 MHz, $CDCl_3$) δ 171.7, 164.2 (d, *J* = 239.1 Hz), 155.0, 147.7 (d, *J* = 15.1 Hz), 121.1, 109.0 (d, *J* = 37.8 Hz), 81.7, 50.1, 37.4, 36.8 (isomer a and b), 32.7 (isomer a), 32.6 (isomer b), 28.7 (isomer a and b), 28.0, 25.7 (isomer a), 25.6 (isomer b), 23.2 (isomer a), 23.1 (isomer b), 14.2, 10.6.

^{19}F NMR (564 MHz, $CDCl_3$) δ -68.18 (s)

HRMS (ESI) m/z: exact mass calculated for $C_{19}H_{31}FNO_2$ [$(M+H)^+$]: 324.2333, Found: 324.23334

Yield determination of non-isolated products

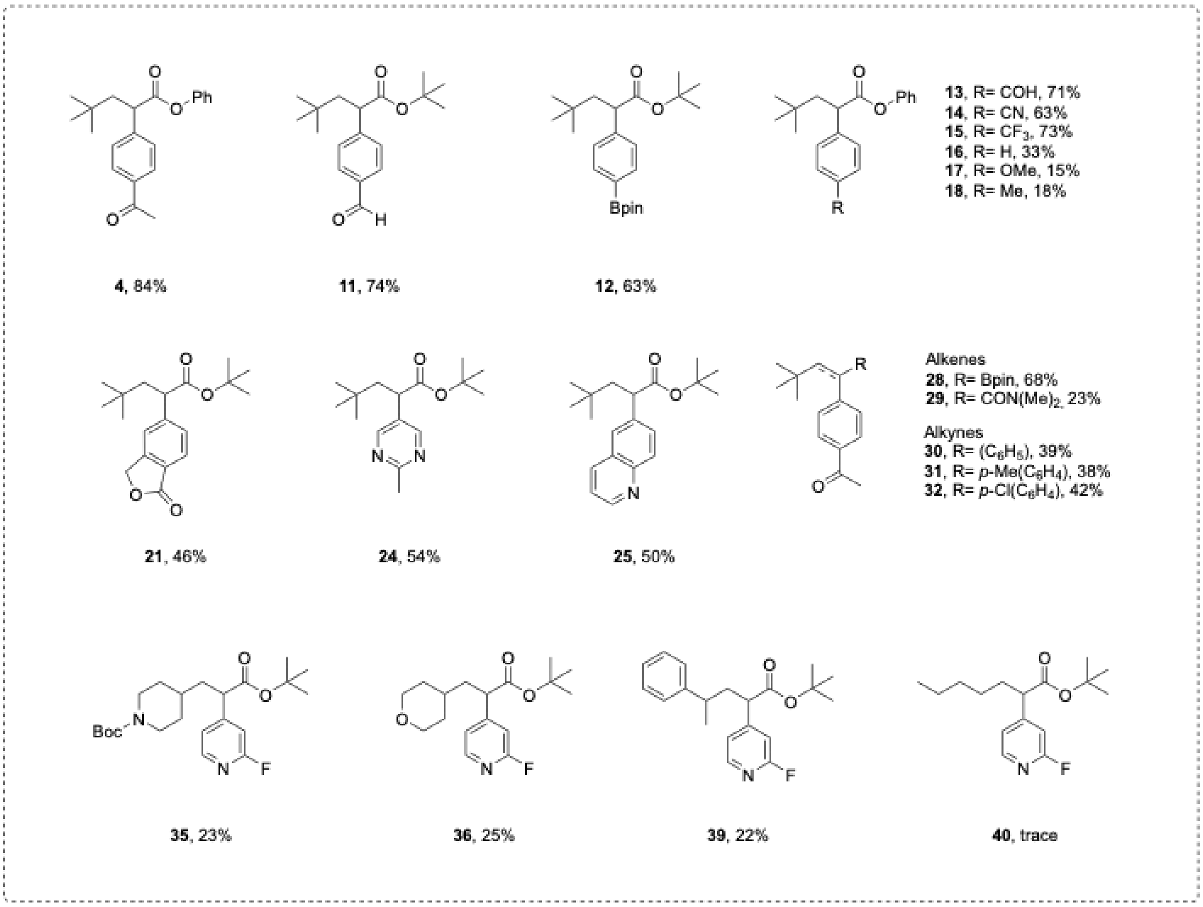

Compounds 4, 11–18, 21, 24–25, 28–32, 35–36, 39–40 were synthesized following GP1. The corresponding yields were determined using ethylene carbonate as internal standard. The identification of the targeted products was based on GC-MS and crude NMR.

Synthesis of starting materials, ligands and catalysts

Tertiary and secondary alcohols were prepared in accordance with previously reported procedures.[6, 7]

$NiBr_2$•dtbbpy was prepared in accordance with previously reported procedure.[7, 8]

Chiral ligand was prepared in accordance with previously reported procedures.[9]

Reference list

(1) Wang, Y.-Z.; Sun, B.; Zhu, X.-Y.; Gu, Y.-C.; Ma, C.; Mei, T.-S. Enantioselective Reductive Cross-Couplings of Olefins by Merging Electrochemistry with Nickel Catalysis. *J Am Chem Soc* **2023**, *145* (44), 23910-23917. DOI: 10.1021/jacs.3c10109.

(2) Guo, L.; Yuan, M.; Zhang, Y.; Wang, F.; Zhu, S.; Gutierrez, O.; Chu, L. General Method for Enantioselective Three-Component Carboarylation of Alkenes Enabled by Visible-Light Dual Photoredox/Nickel Catalysis. *J Am Chem Soc* **2020**, *142* (48), 20390-20399. DOI: 10.1021/jacs.0c08823.

(3) Xu, L.; Zhang, F.; Wang, Y.-E.; Bai, C.; Xiong, D.; Mao, J. Cobalt-Catalyzed Three-Component Alkyl Arylation of Acrylates with Alkyl Iodides and Aryl Grignard Reagents. *Organic Letters* **2024**, *26* (43), 9288-9293. DOI: 10.1021/acs.orglett.4c03453.

(4) Qian, P.; Guan, H.; Wang, Y.-E.; Lu, Q.; Zhang, F.; Xiong, D.; Walsh, P. J.; Mao, J. Catalytic enantioselective reductive domino alkyl arylation of acrylates via nickel/photoredox catalysis. *Nat Commun* **2021**, *12* (1), 6613. DOI: 10.1038/s41467-021-26794-8.

(5) Zou, L.; Zheng, X.; Yi, X.; Lu, Q. Asymmetric paired oxidative and reductive catalysis enables enantioselective alkylarylation of olefins with C(sp3)−H bonds. *Nat Commun* **2024**, *15* (1), 7826. DOI: 10.1038/s41467-024-52248-y.

(6) Patehebieke, Y.; Charaf, R.; Bryce-Rogers, H. P.; Ye, K.; Ahlquist, M.; Hammarström, L.; Wallentin, C.-J. β-Scission of Secondary Alcohols via Photosensitization: Synthetic Utilization and Mechanistic Insights. *Acs Catal* **2024**, *14* (1), 585-593. DOI: 10.1021/acscatal.3c05150.

(7) Patehebieke, Y.; Charaf, R.; Pal, K. B.; Baamonde, B. M.; Brnovic, A.; Hammarström, L.; Wallentin, C.-J. PCET-mediated deconstructive cross-coupling of aliphatic alcohols. *Chem Sci* **2025**, *16* (18), 7720-7729, 10.1039/D5SC00737B. DOI: 10.1039/D5SC00737B.

(8) Gao, Y.; Yang, C.; Bai, S.; Liu, X.; Wu, Q.; Wang, J.; Jiang, C.; Qi, X. Visible-Light-Induced Nickel-Catalyzed Cross-Coupling with Alkylzirconocenes from Unactivated Alkenes. *Chem-Us* **2020**, *6* (3), 675-688. DOI: https://doi.org/10.1016/j.chempr.2019.12.010.

(9) Cheng, X.; Li, T.; Liu, Y.; Lu, Z. Stereo- and Enantioselective Benzylic C–H Alkenylation via Photoredox/Nickel Dual Catalysis. *Acs Catal* **2021**, *11* (17), 11059-11065. DOI: 10.1021/acscatal.1c02851.

NMR spectra of isolated products

tert-Butyl 2-(4-cyanophenyl)-4,4-dimethylpentanoate (5)

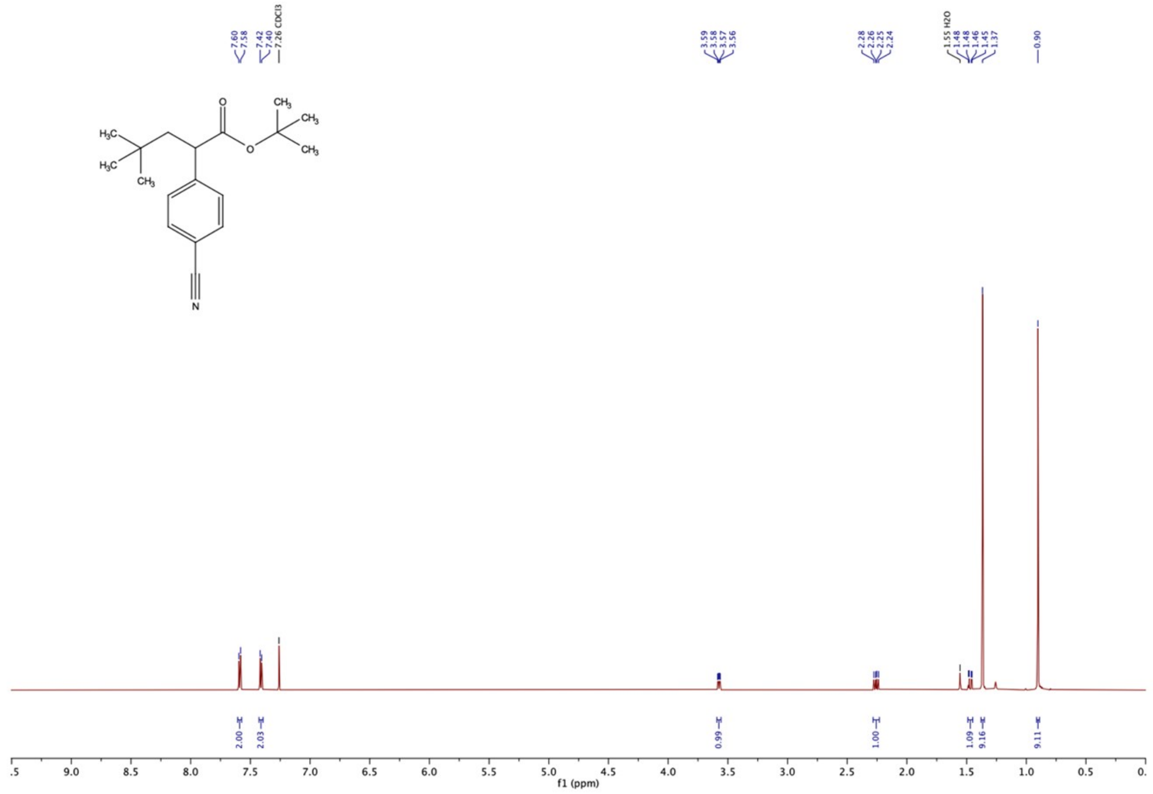

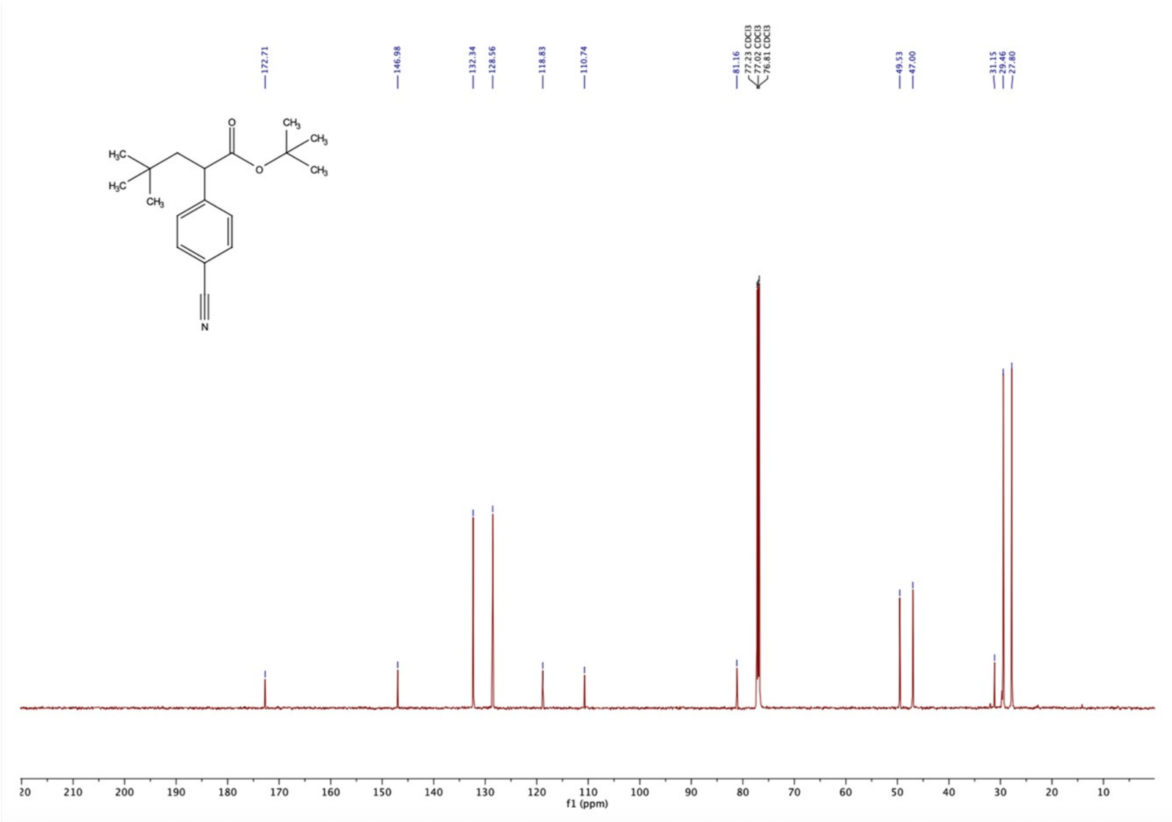

tert-Butyl 4,4-dimethyl-2-(4-(methylsulfonyl)phenyl)pentanoate (6)

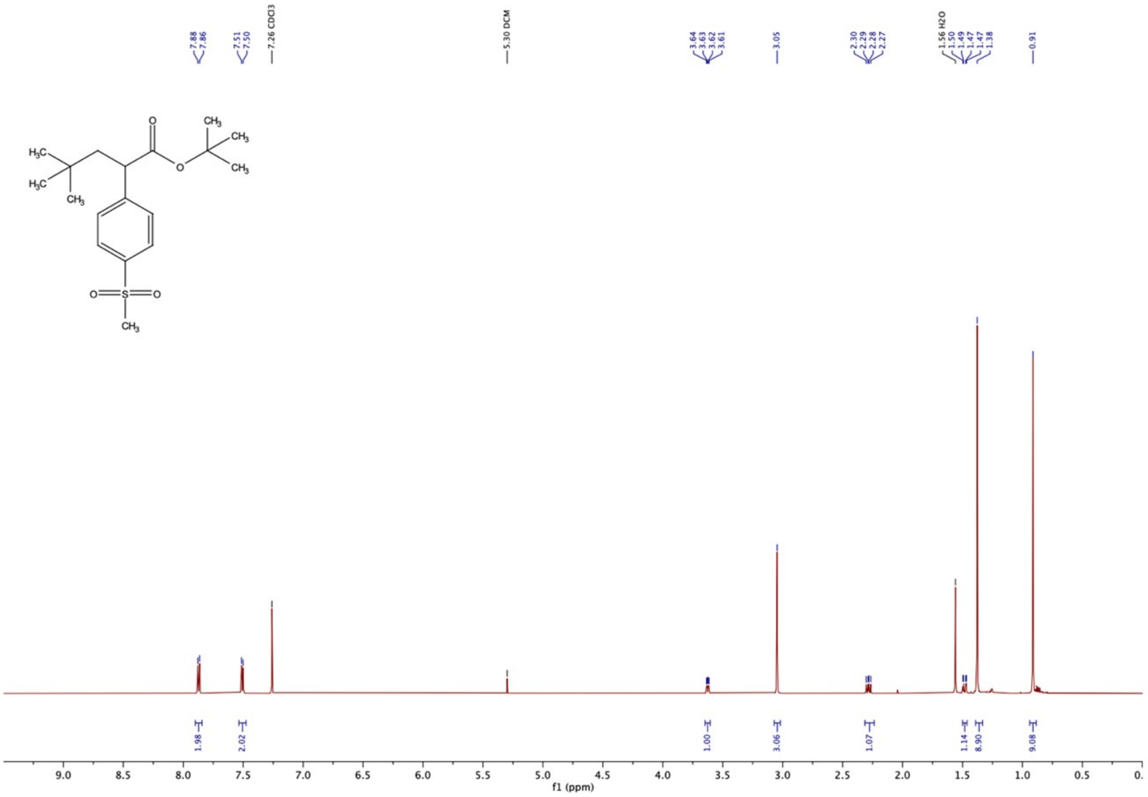

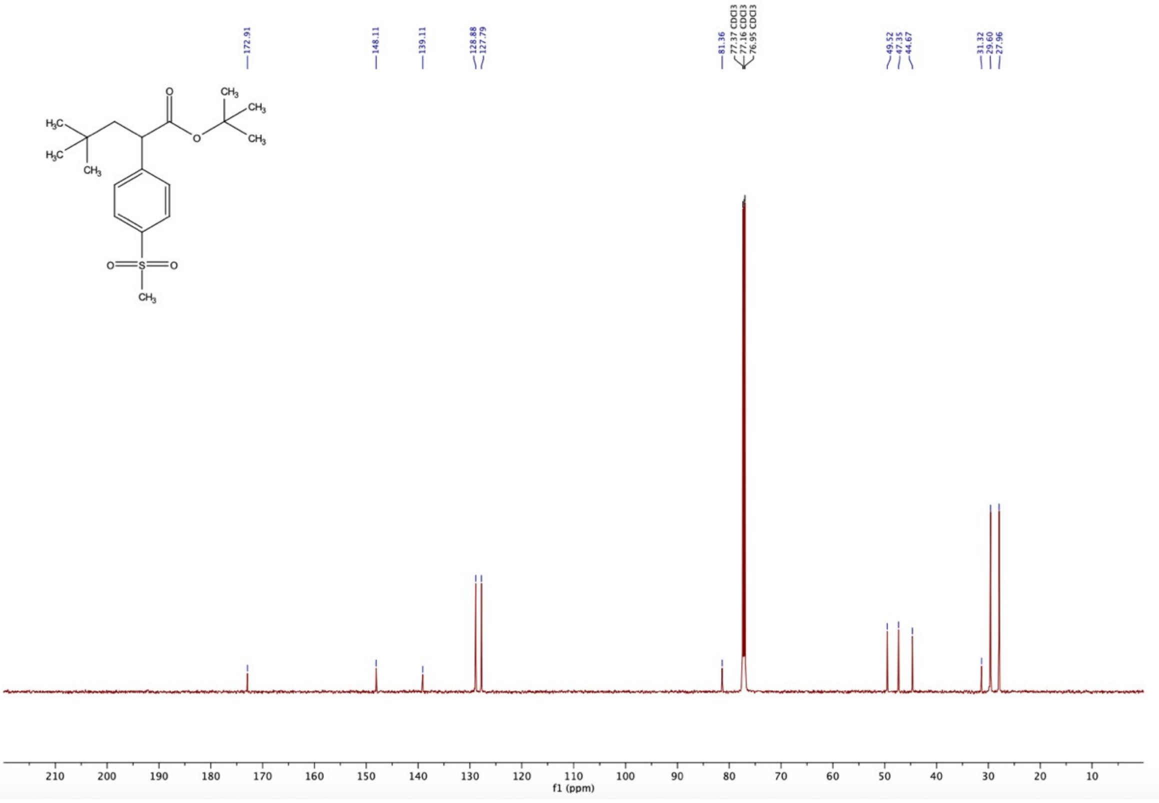

tert-Butyl 4,4-dimethyl-2-(4-(trifluoromethyl)phenyl)pentanoate (7)

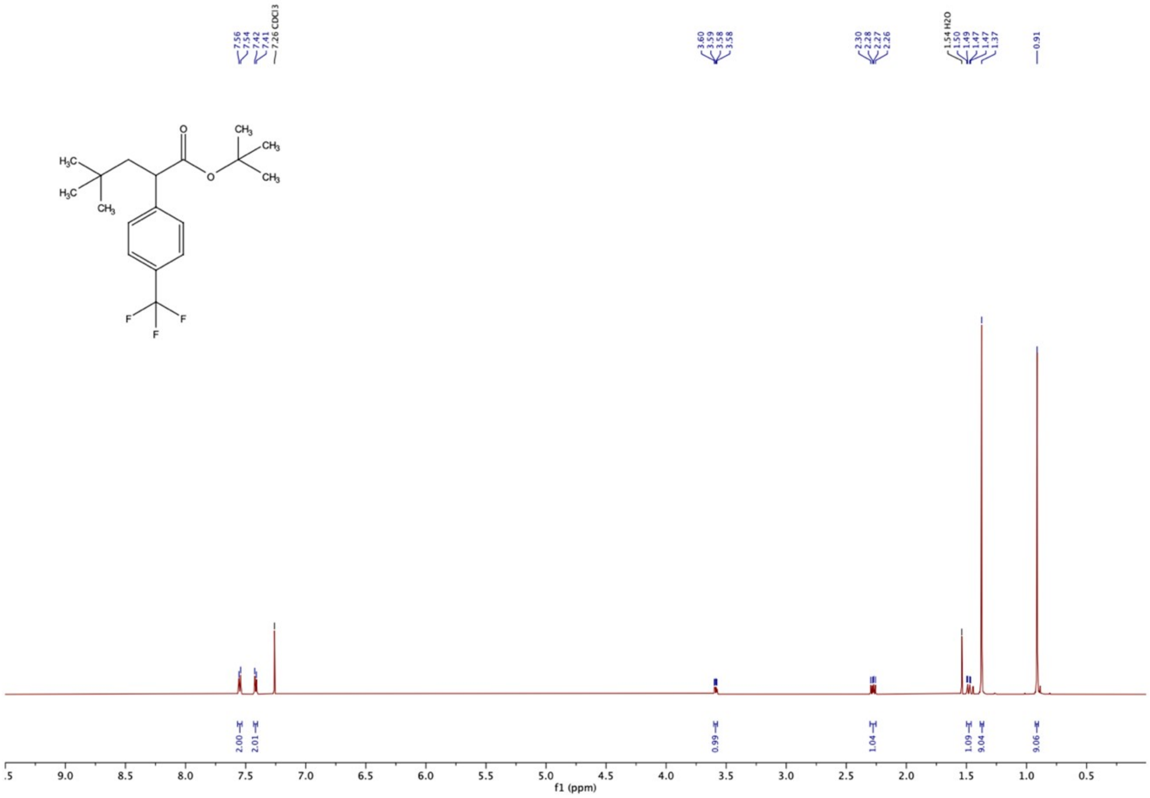

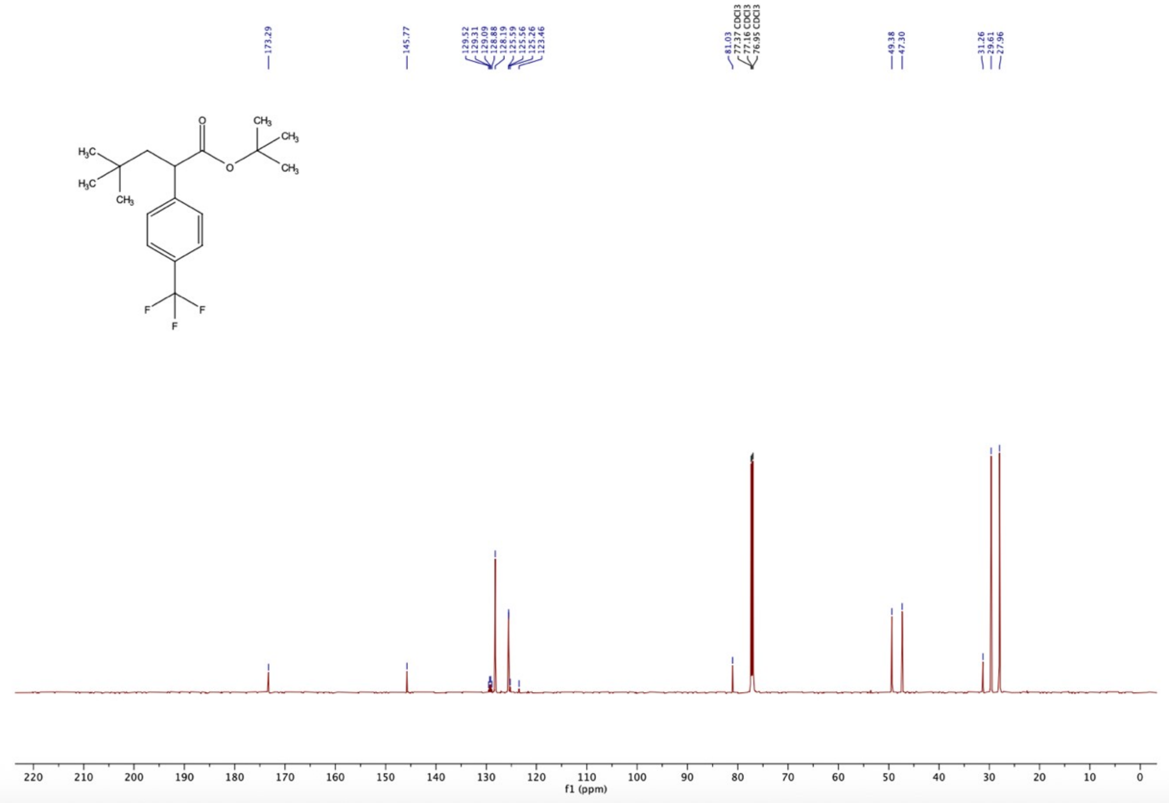

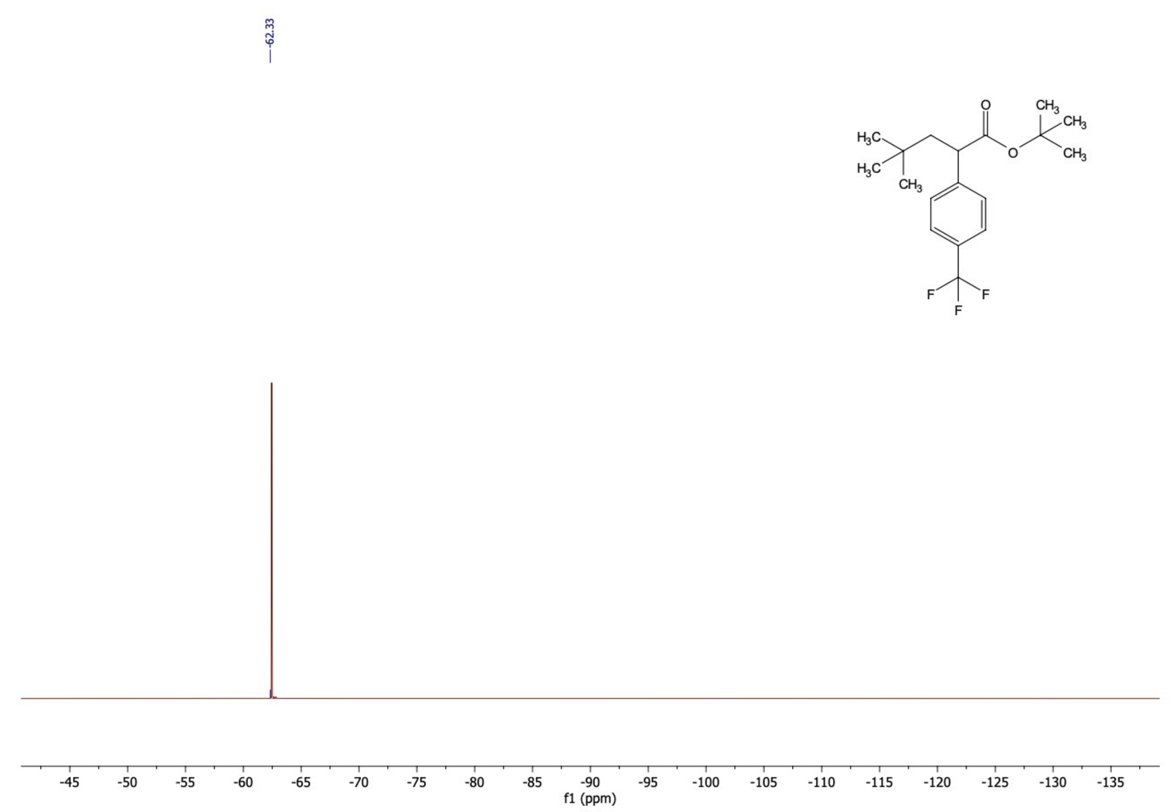

***tert*-Butyl 2-(4-acetylphenyl)-4,4-dimethylpentanoate (8)**

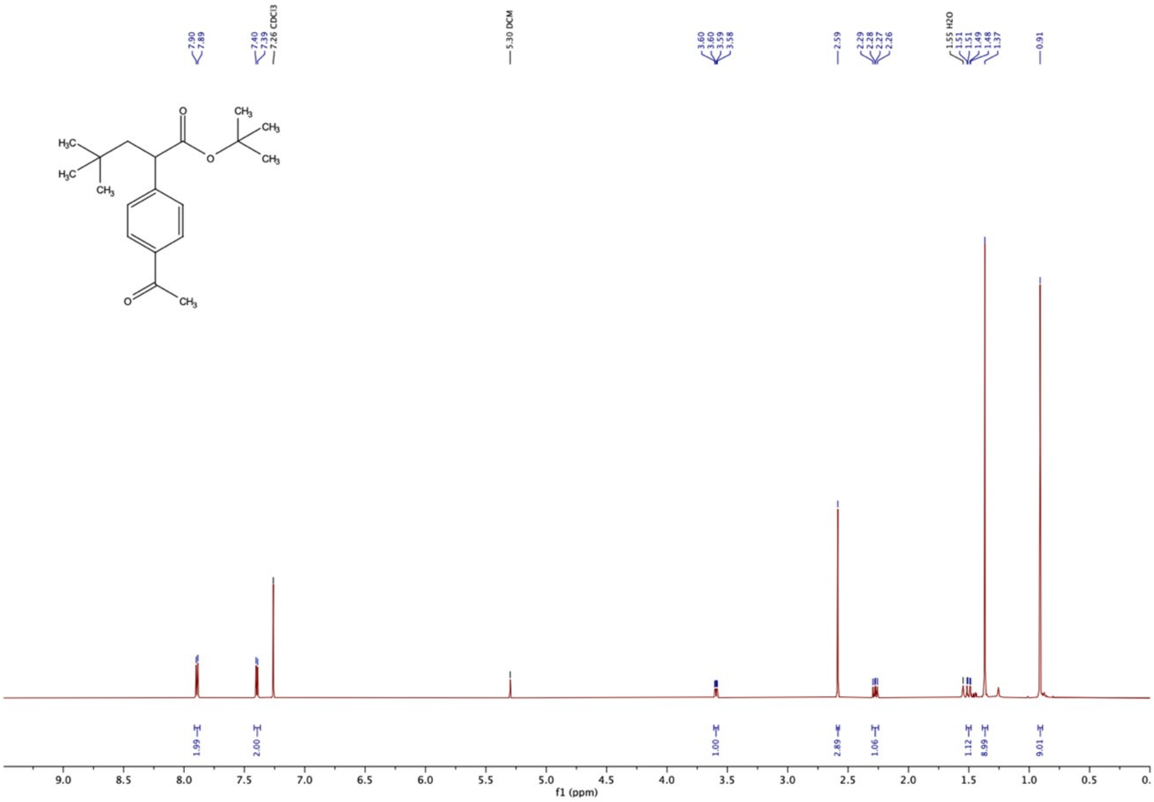

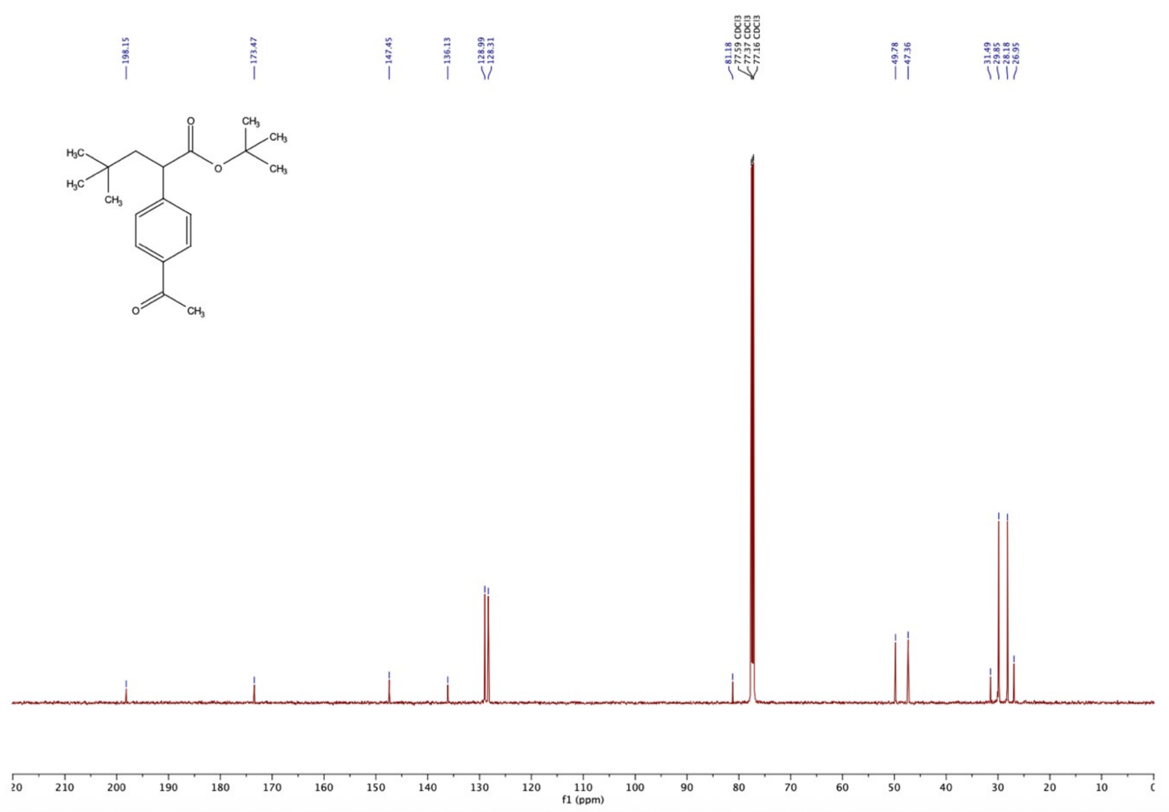

Methyl 4-(1-(*tert*-butoxy)-4,4-dimethyl-1-oxopentan-2-yl)benzoate (9)

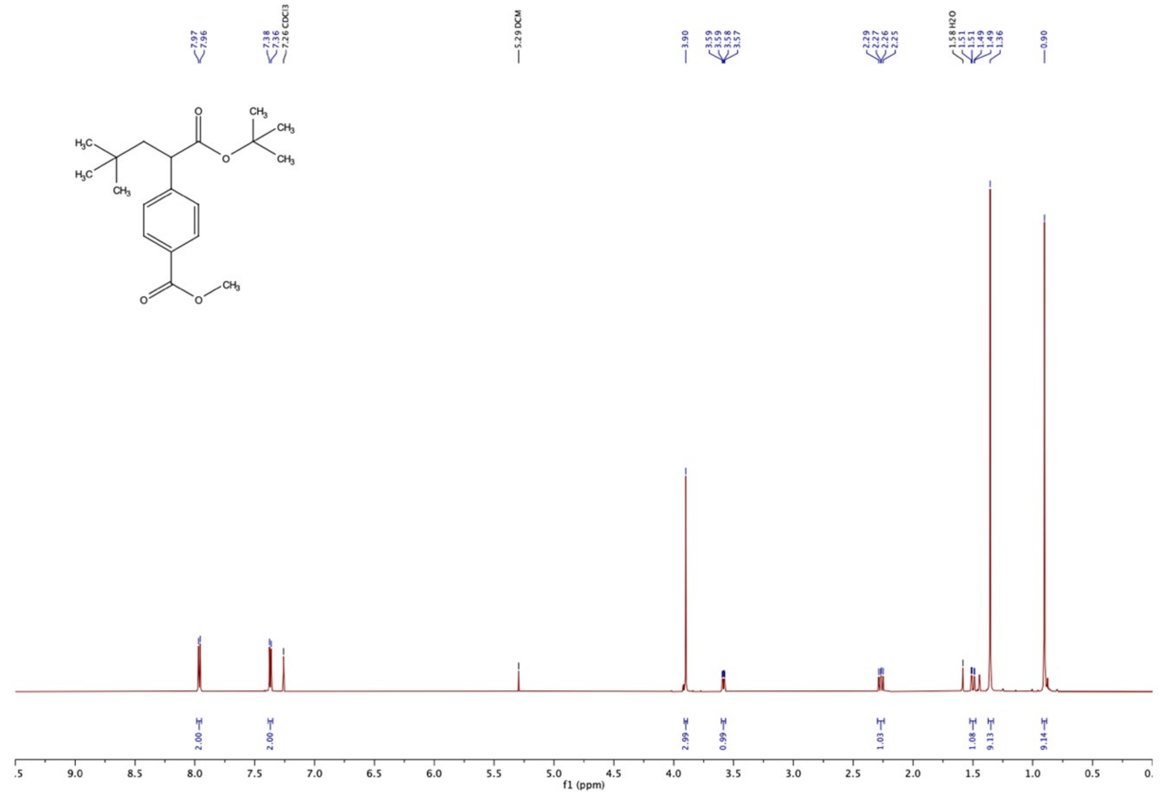

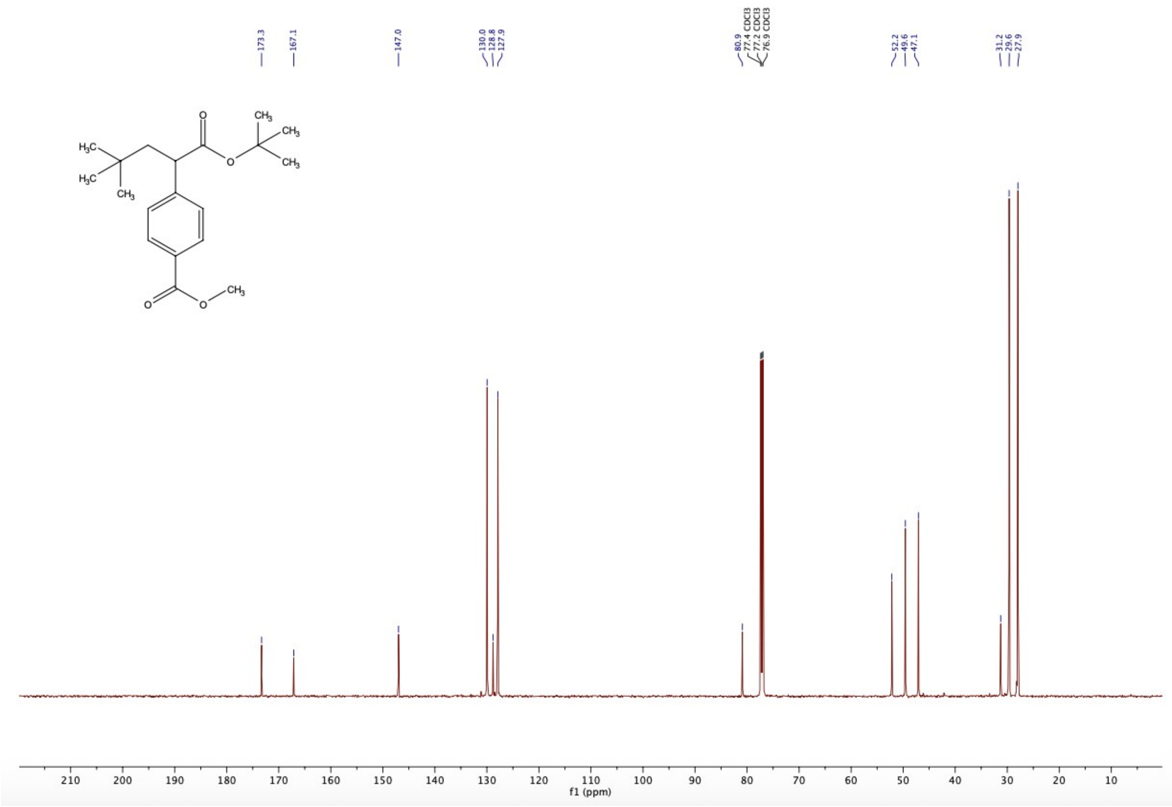

***tert*-Butyl 4,4-dimethyl-2-(4-(morpholinosulfonyl)phenyl)pentanoate (10)**

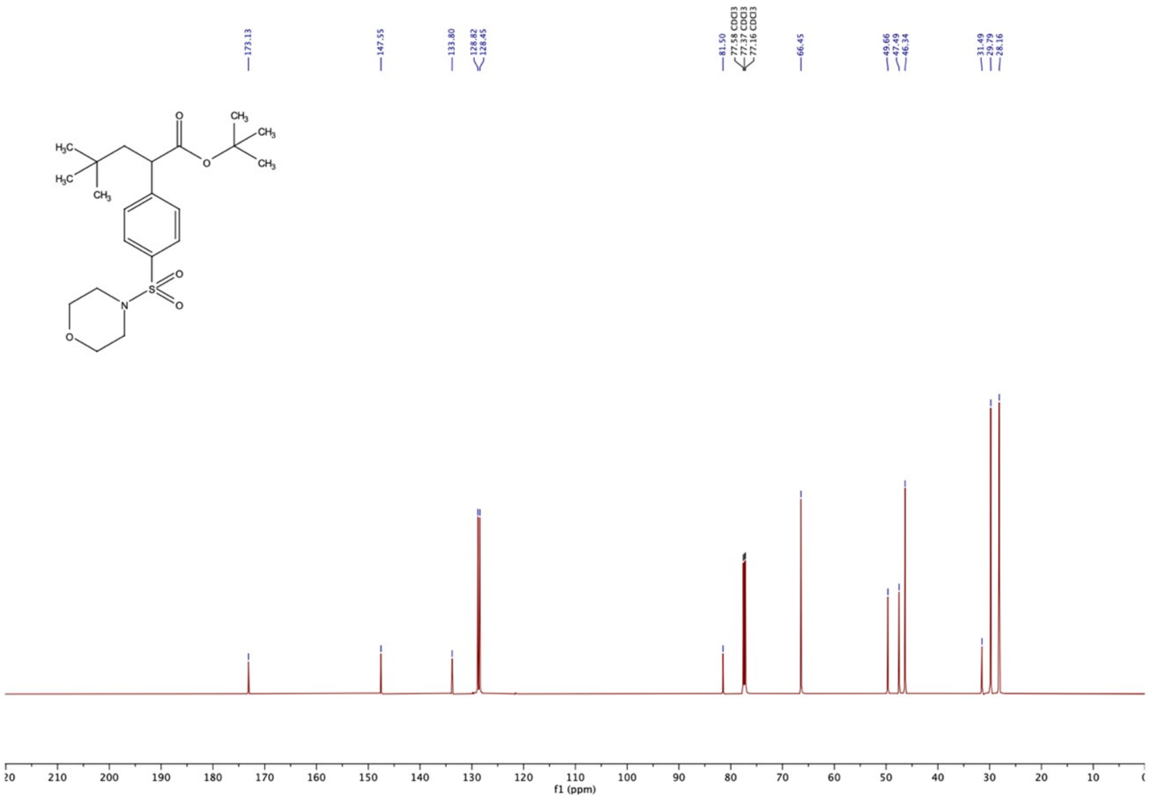

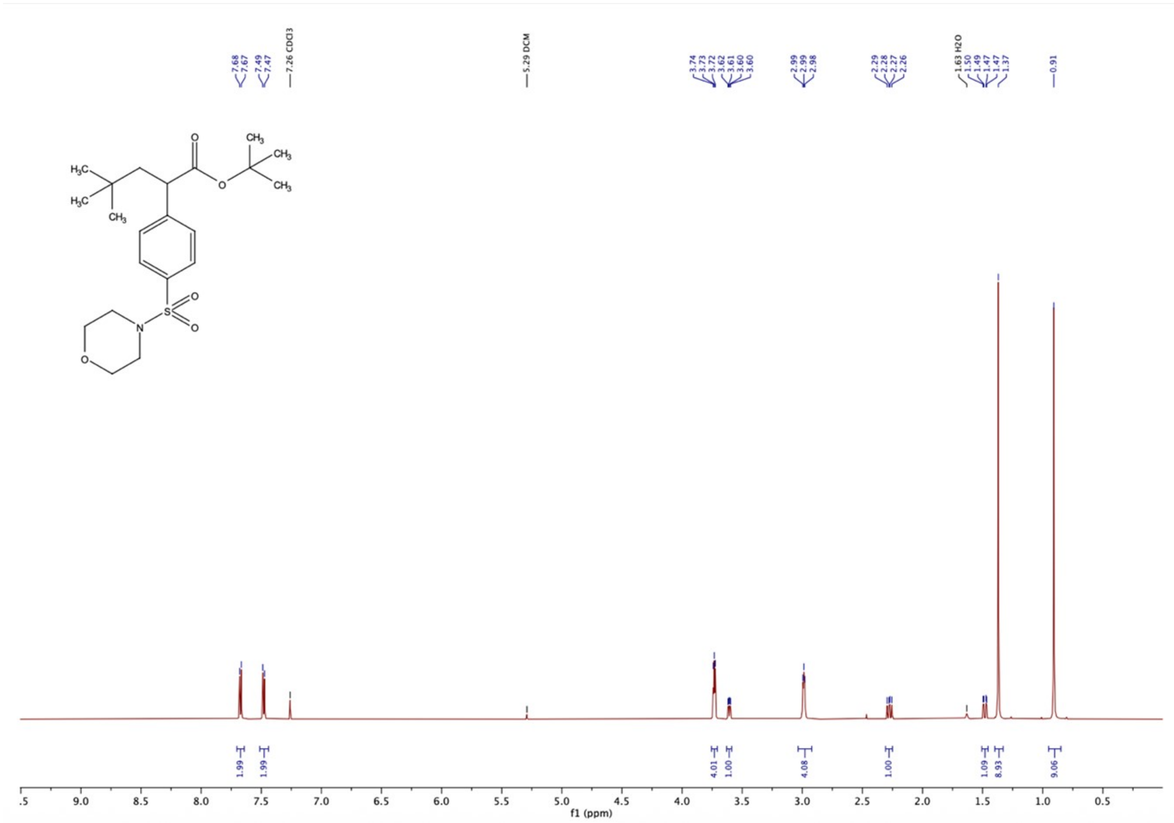

***tert*-Butyl 2-([1,1'-biphenyl]-4-yl)-4,4-dimethylpentanoate (19)**

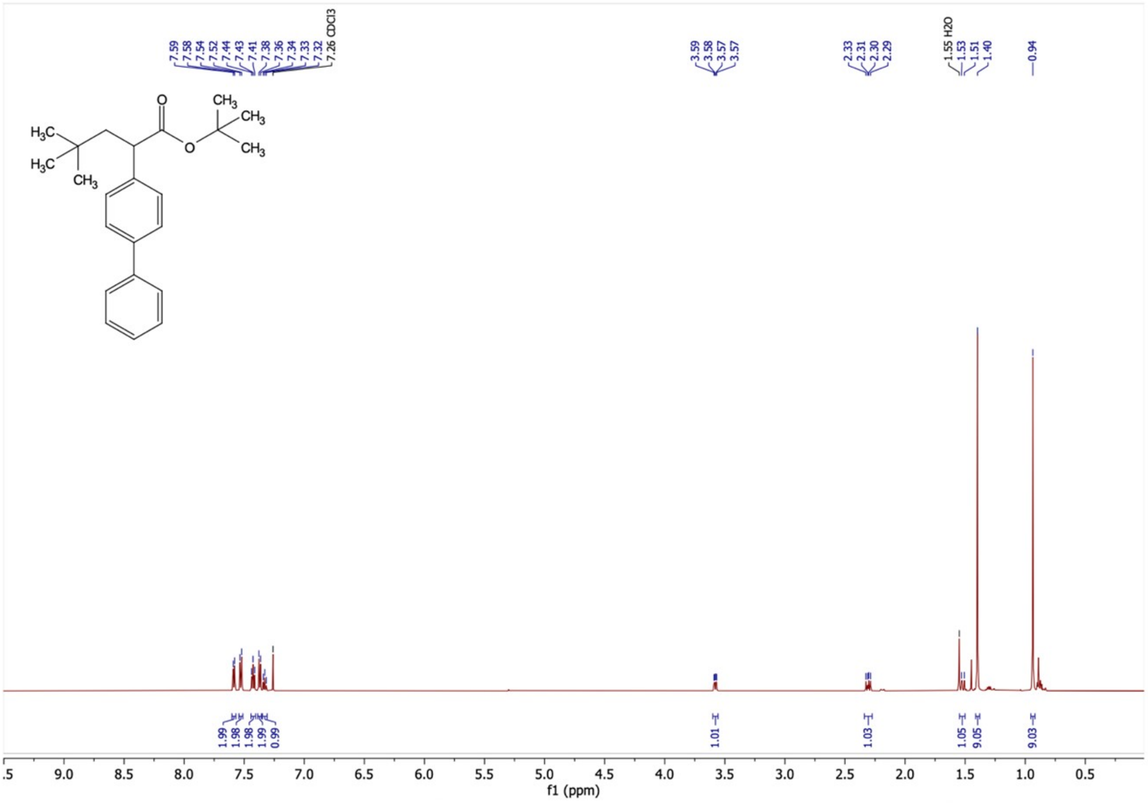

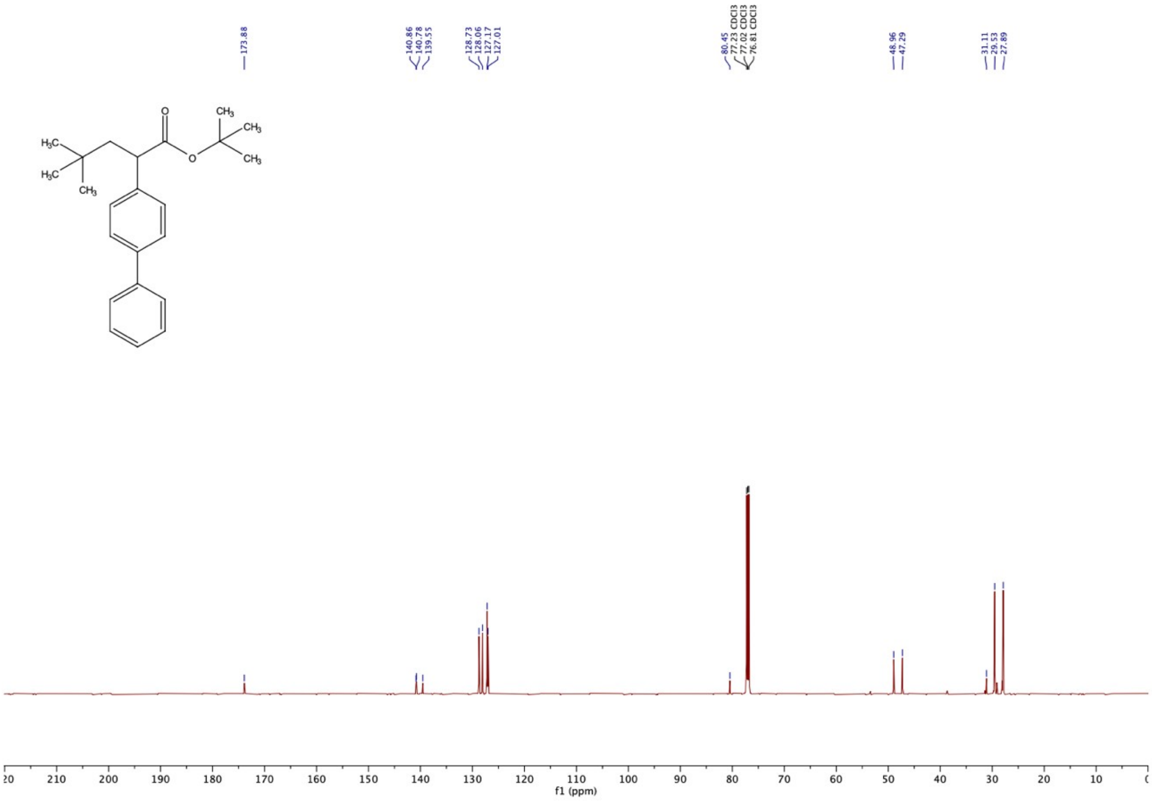

***tert*-Butyl 4,4-dimethyl-2-(naphthalen-2-yl)pentanoate (20)**

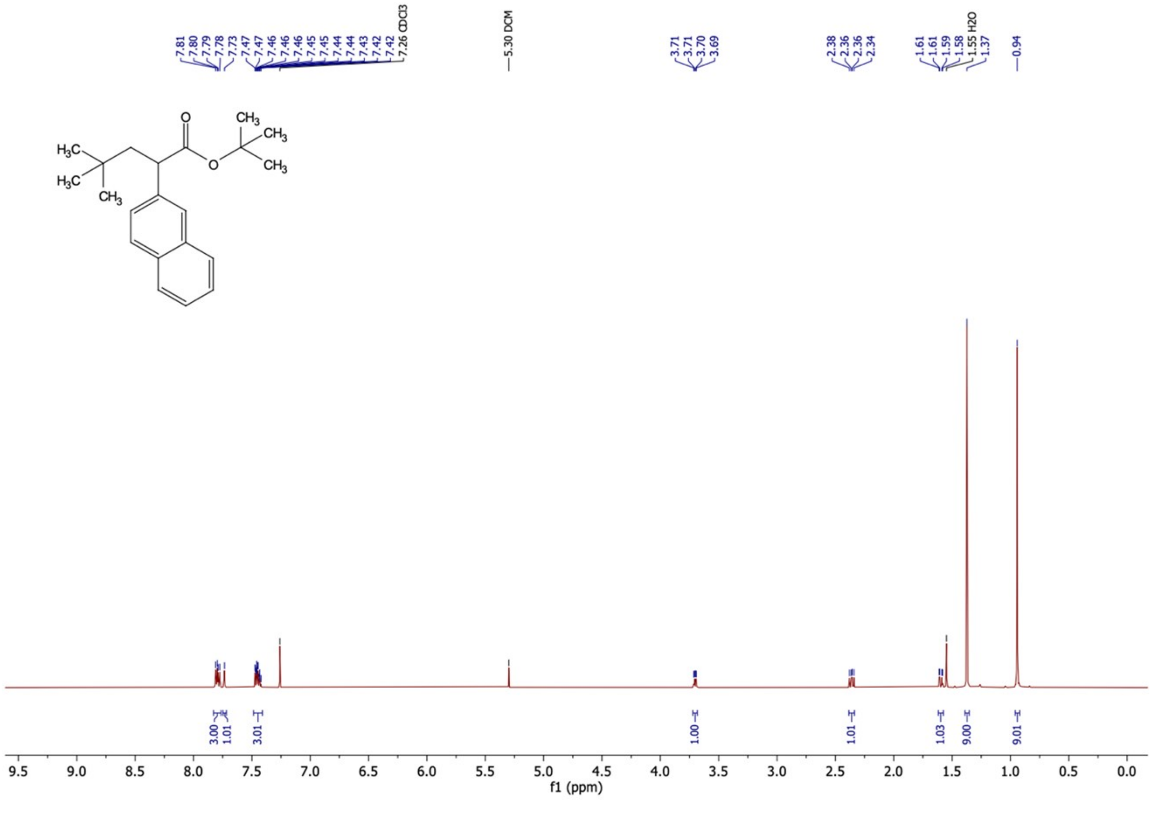

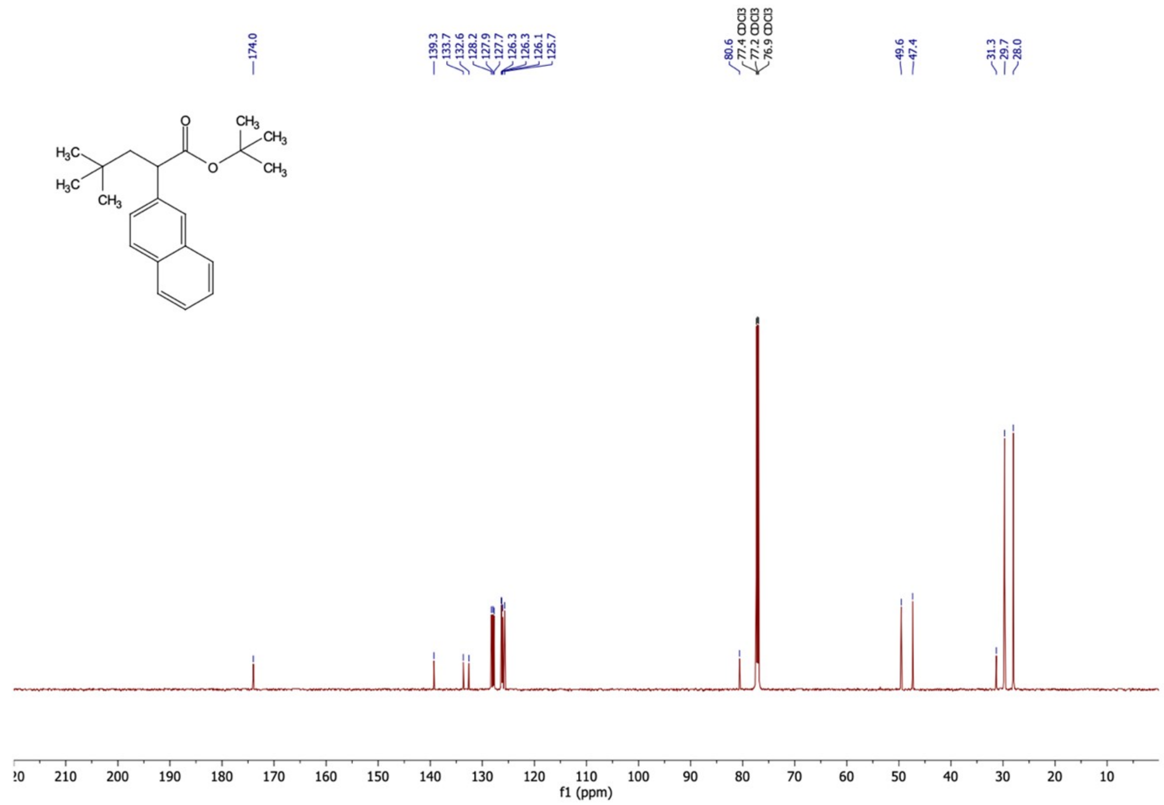

tert-Butyl 2-(2-fluoropyridin-4-yl)-4,4-dimethylpentanoate (22)

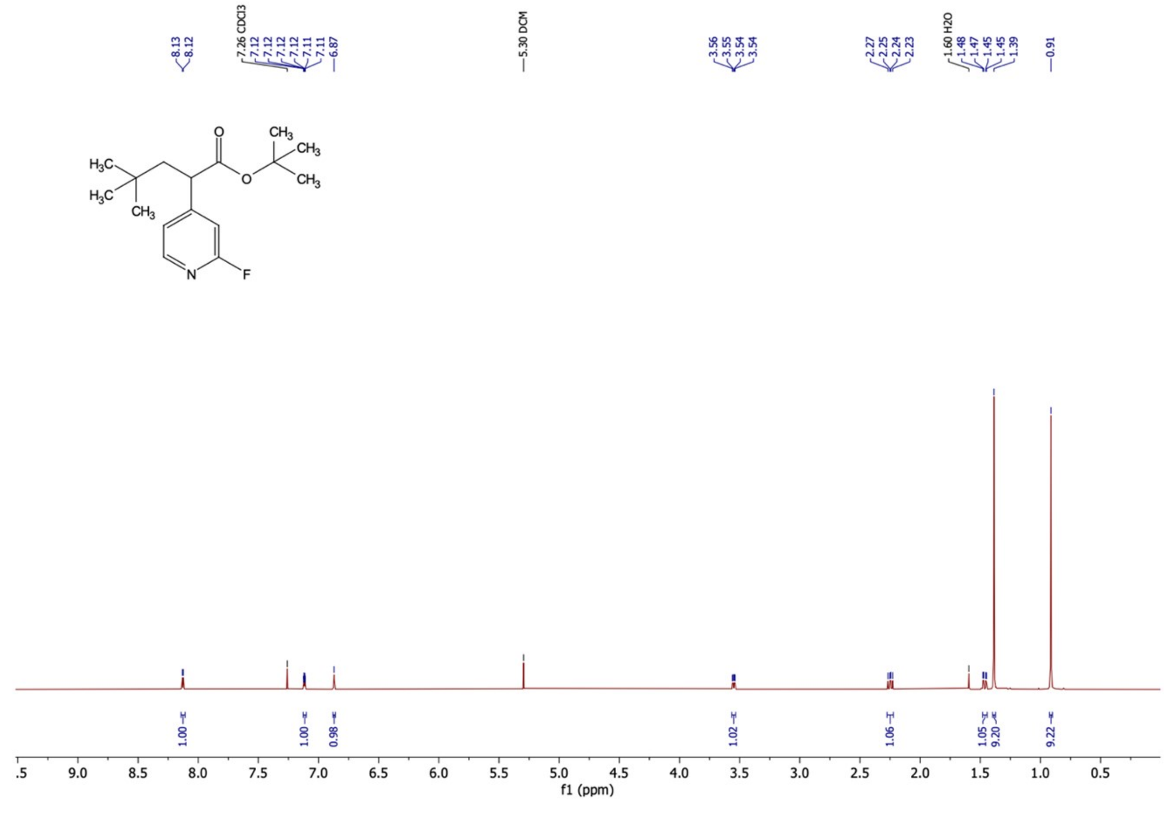

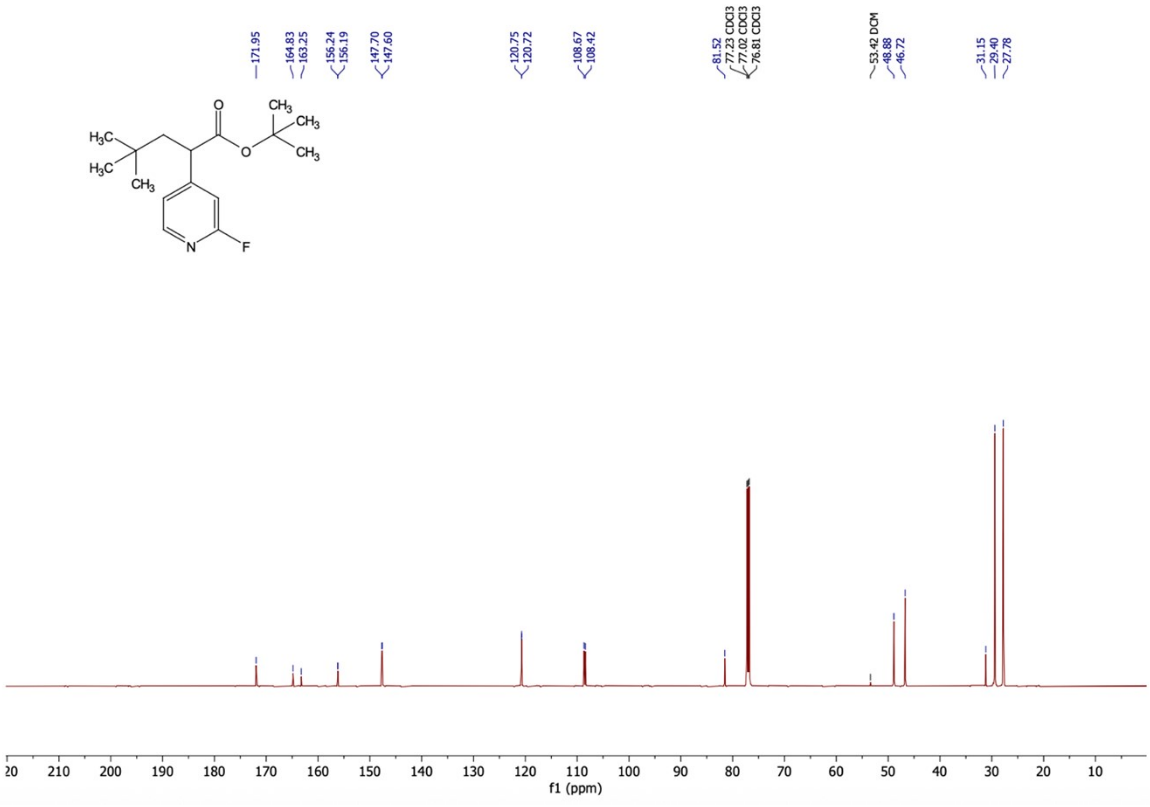
171.95
164.83
163.25
156.24
156.19
147.70
147.60
120.75
120.72
108.67
108.42
81.52
77.23 CDCl3
77.02 CDCl3
76.81 CDCl3
53.42 DCM
48.88
46.72
31.15
29.40
27.78
H3C
H3C
CH3
CH3
CH3
CH3
O
O
N
F
f1 (ppm)

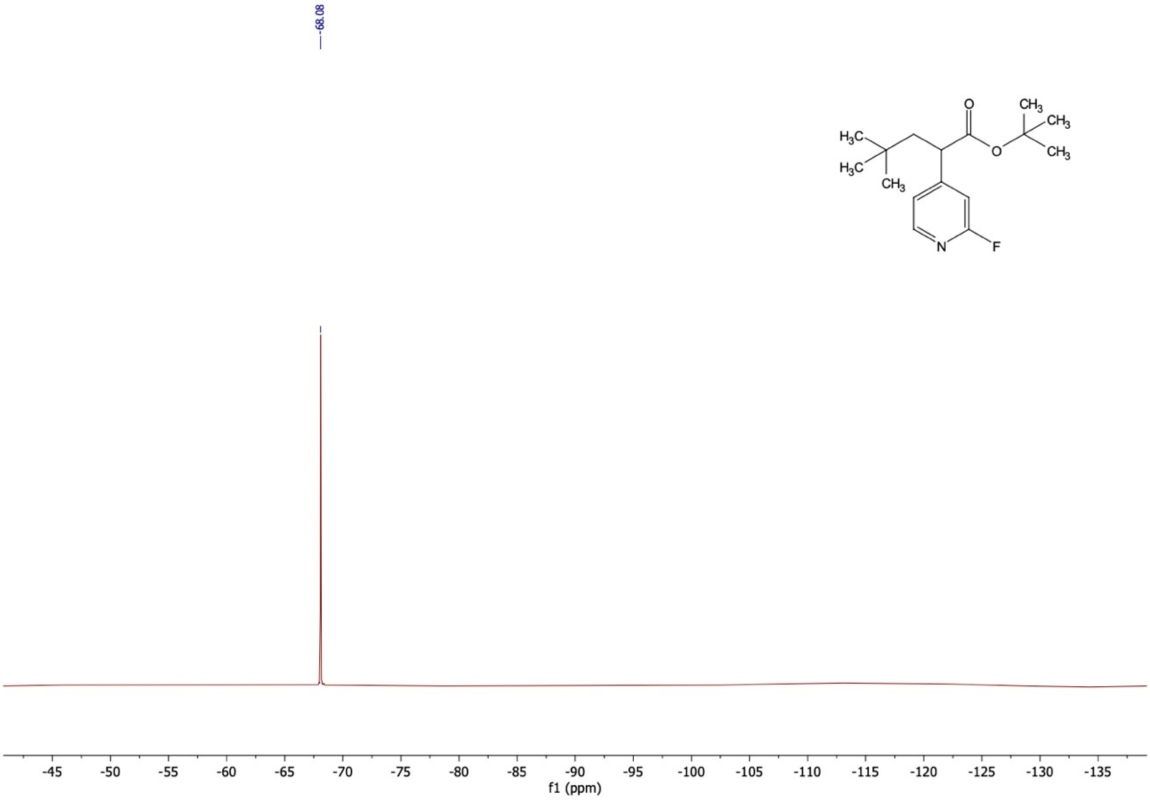
-68.08
H3C
H3C
CH3
CH3
CH3
CH3
O
O
N
F
f1 (ppm)

tert-Butyl 4,4-dimethyl-2-(2-methylquinolin-6-yl)pentanoate (23)

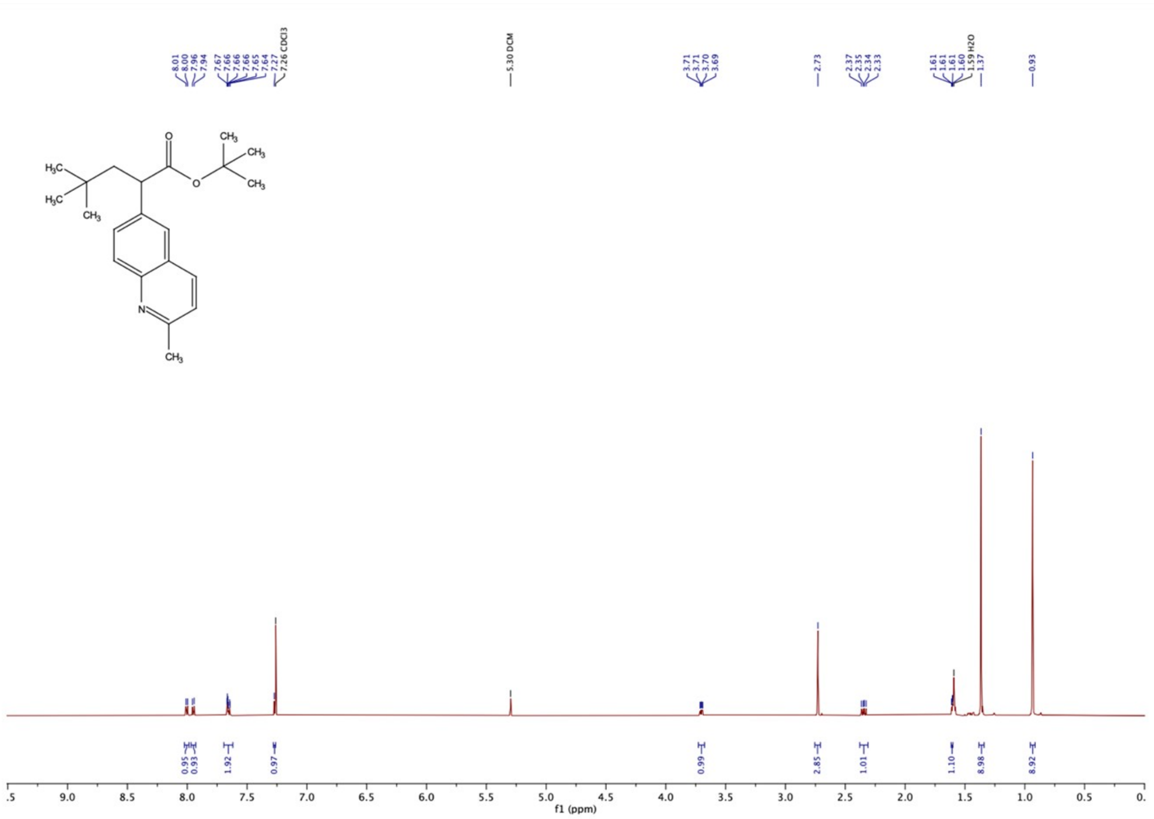

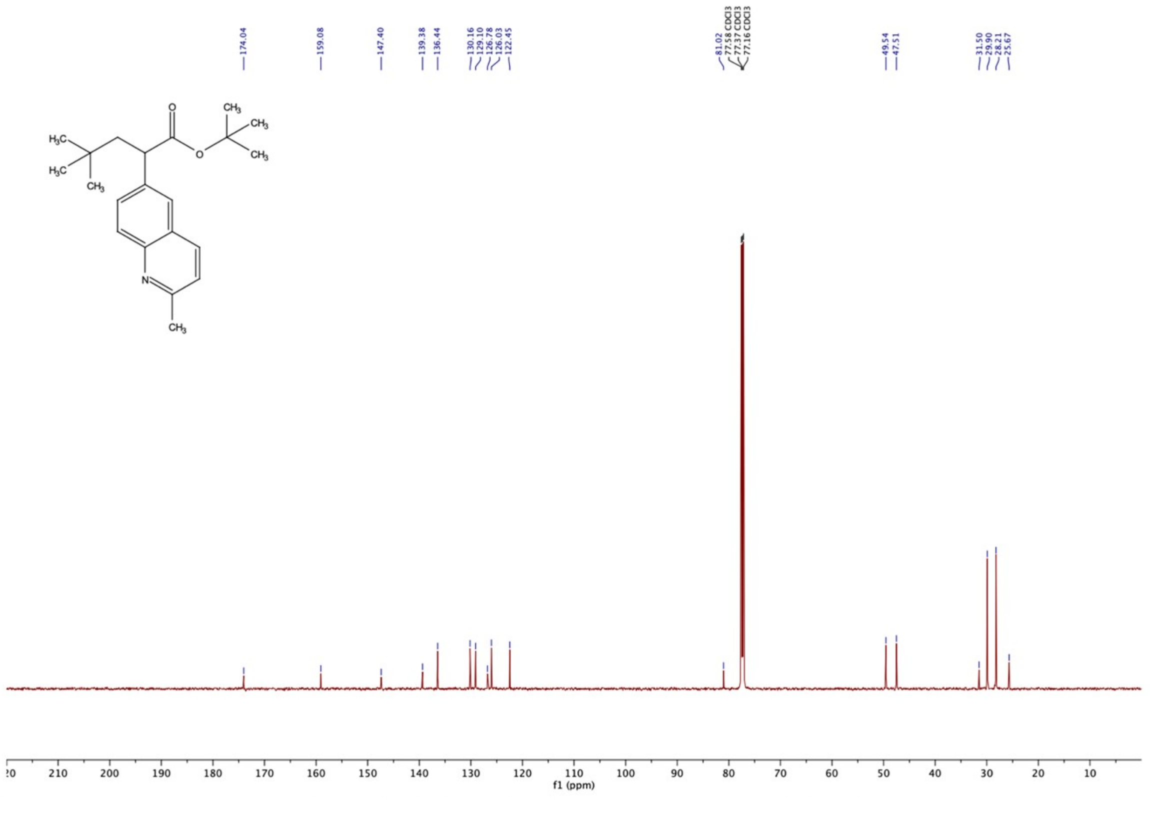

tert-Butyl 3-cyclohexyl-2-(2-fluoropyridin-4-yl)propanoate (33)

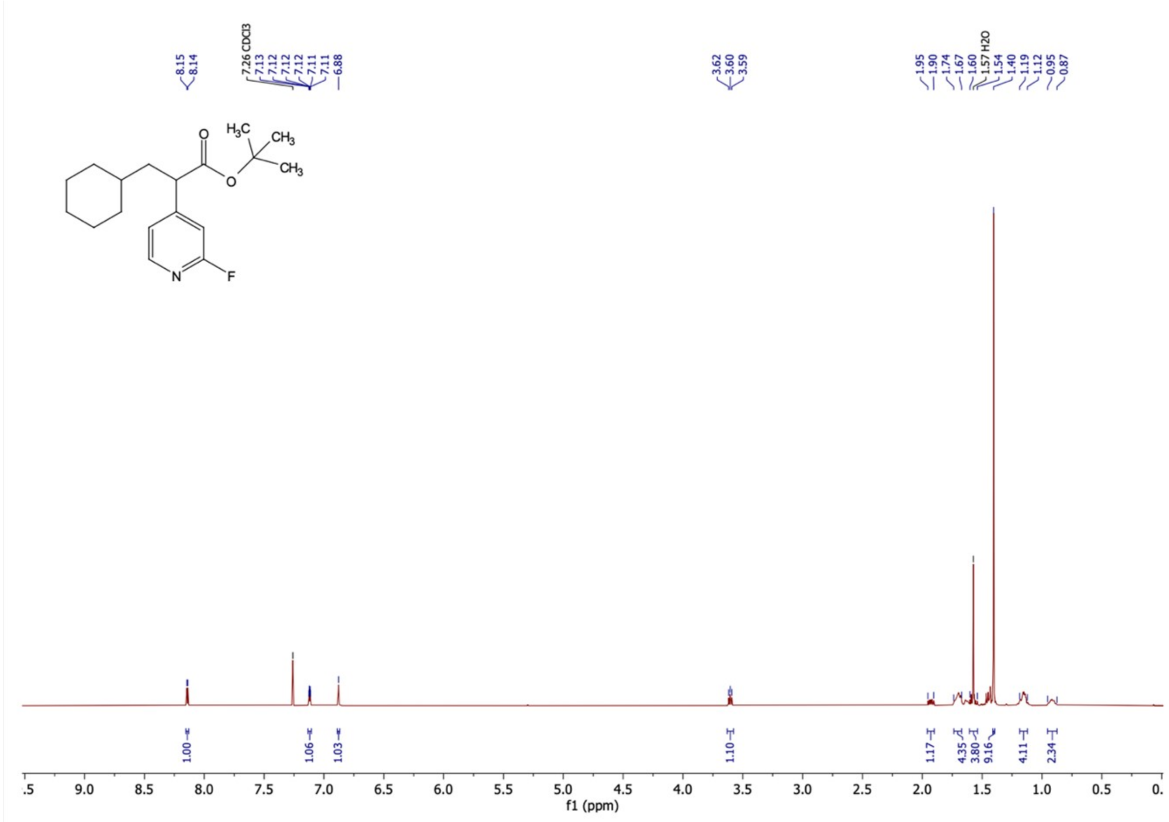

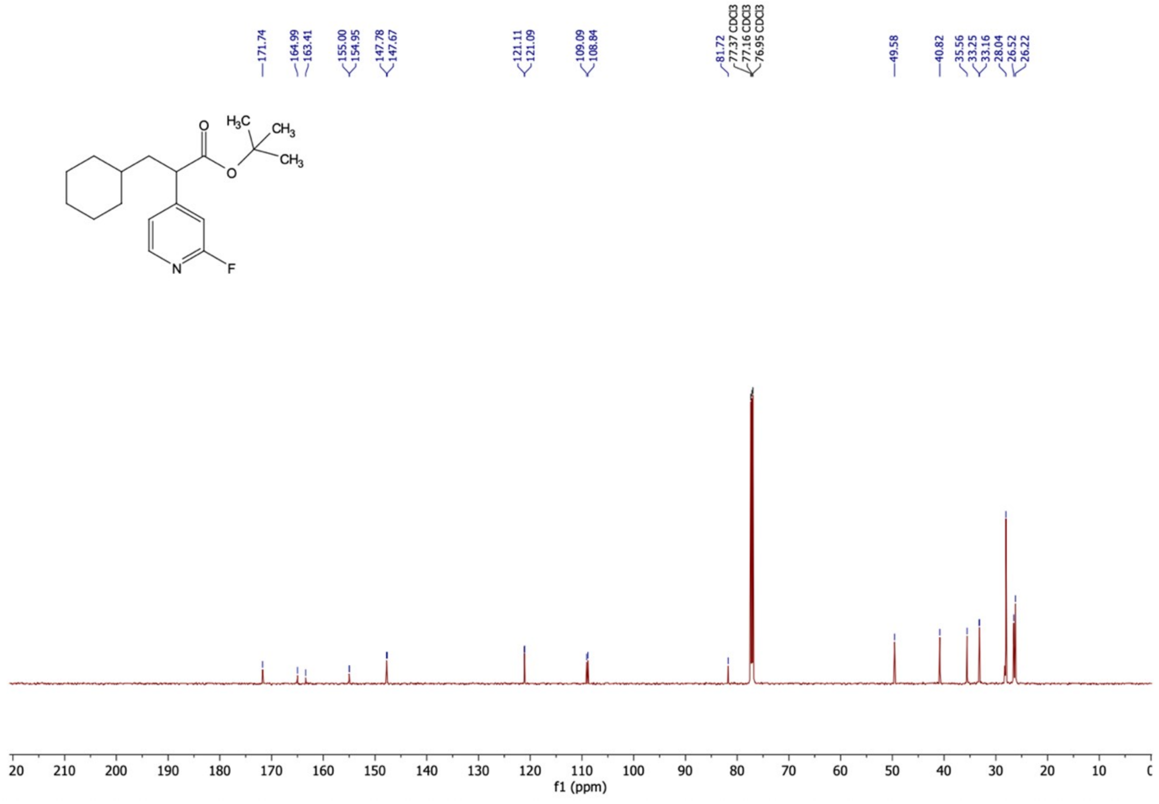

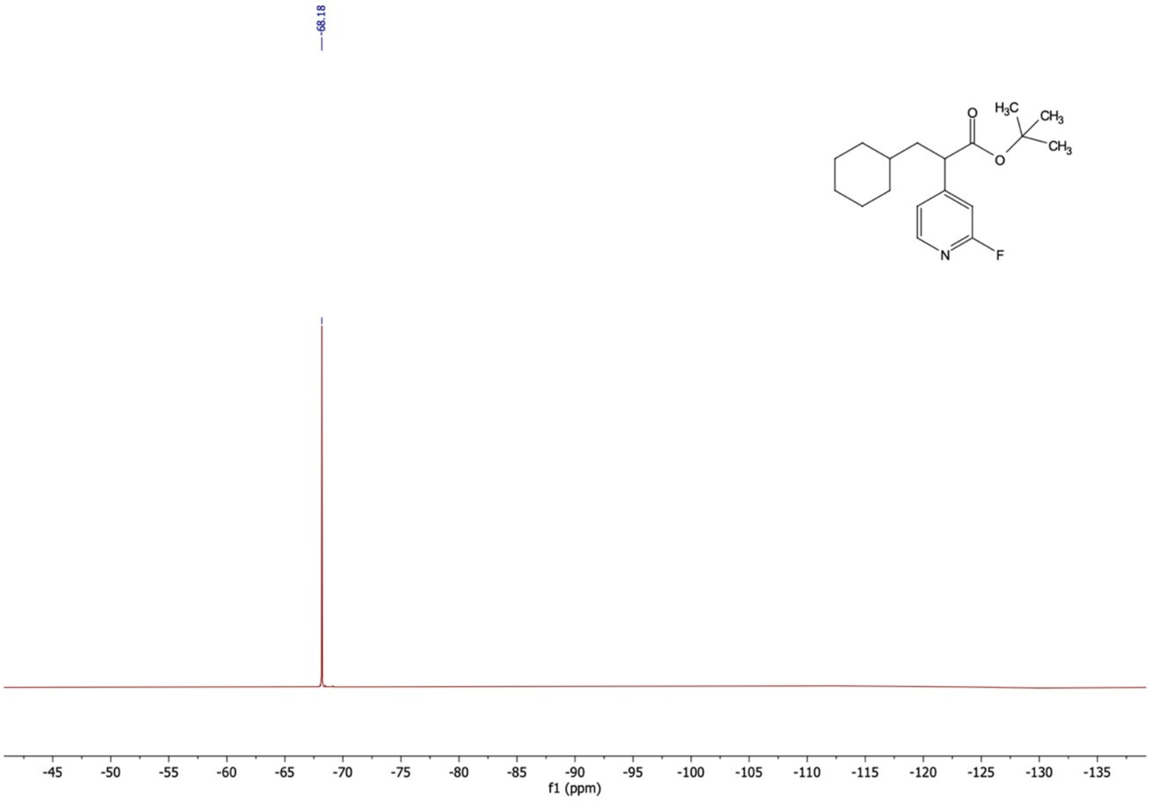

***tert*-Butyl 3-cyclopentyl-2-(2-fluoropyridin-4-yl)propanoate (34)**

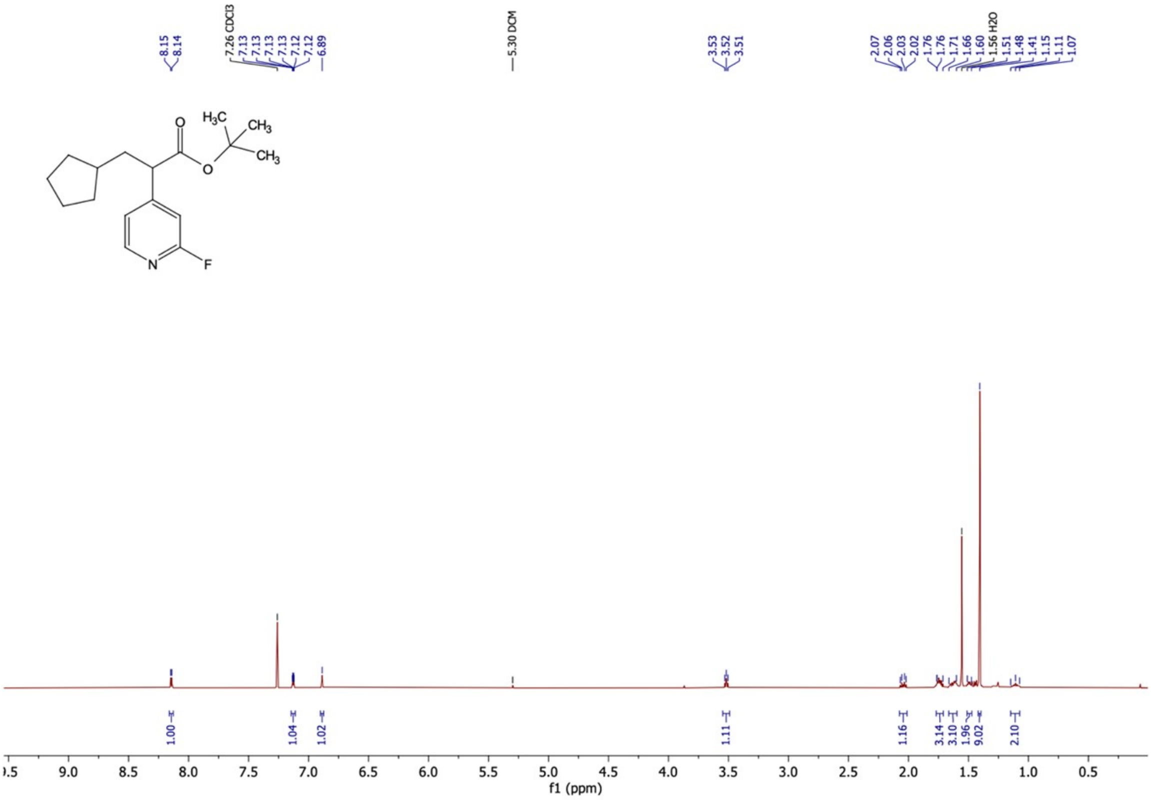

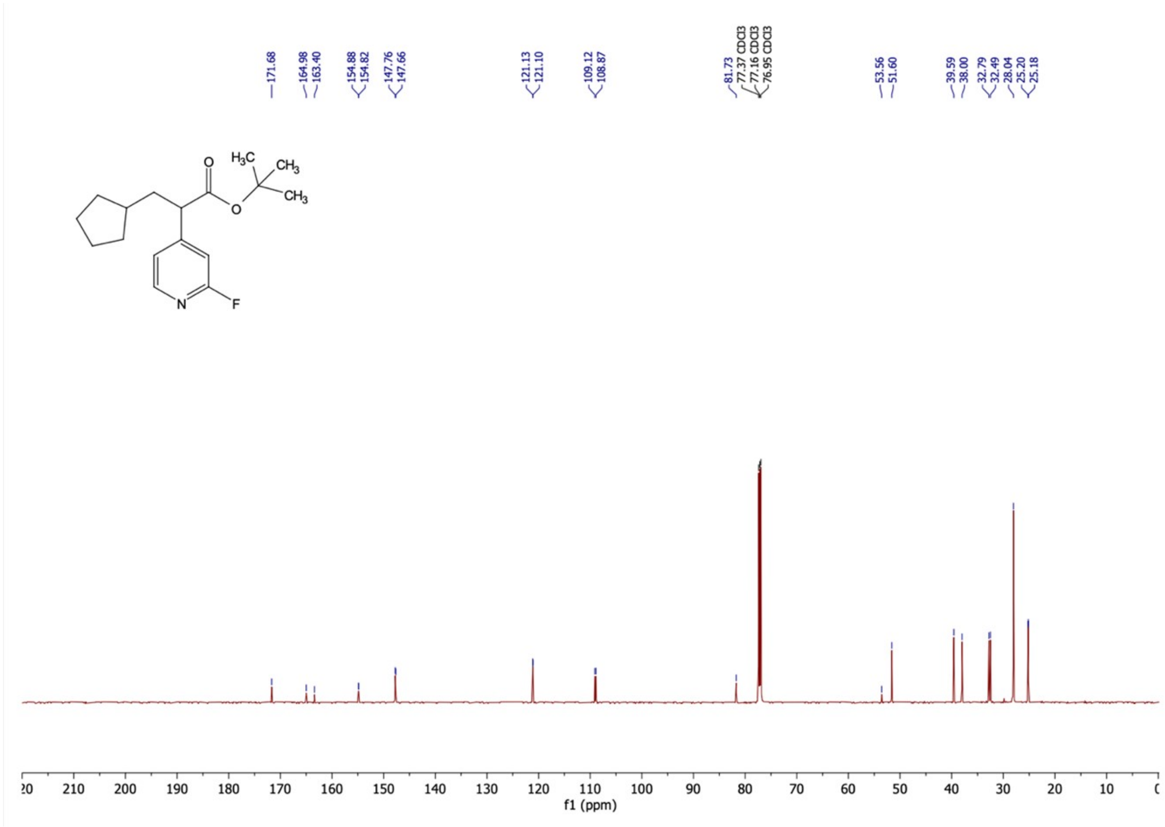
171.68
164.98
163.40
154.88
154.82
147.76
147.66
121.13
121.10
109.12
108.87
81.73
77.37 CDCl3
77.16 CDCl3
76.95 CDCl3
53.56
51.60
39.59
38.00
32.79
32.49
28.04
25.20
25.18
O
H3C
CH3
CH3
O
N
F
f1 (ppm)

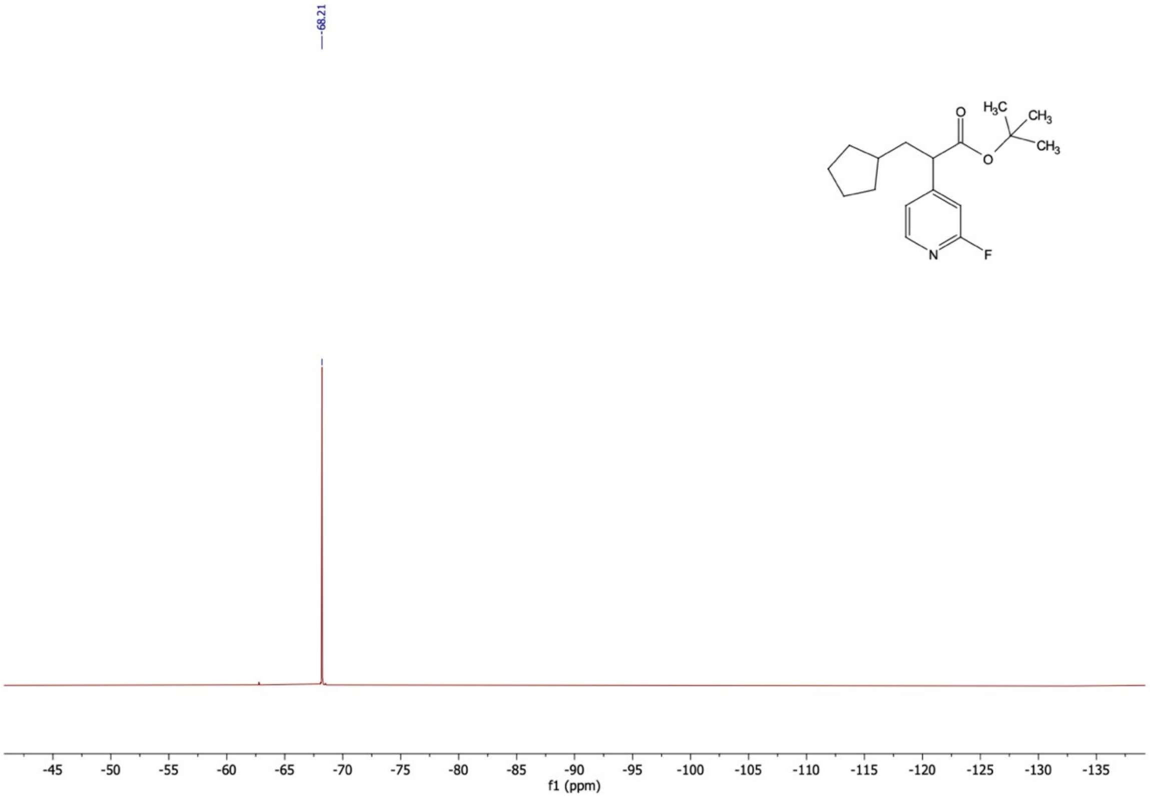
-68.21
O
H3C
CH3
CH3
O
N
F
f1 (ppm)

***tert*-Butyl 2-(2-fluoropyridin-4-yl)-4-methylpentanoate (37)**

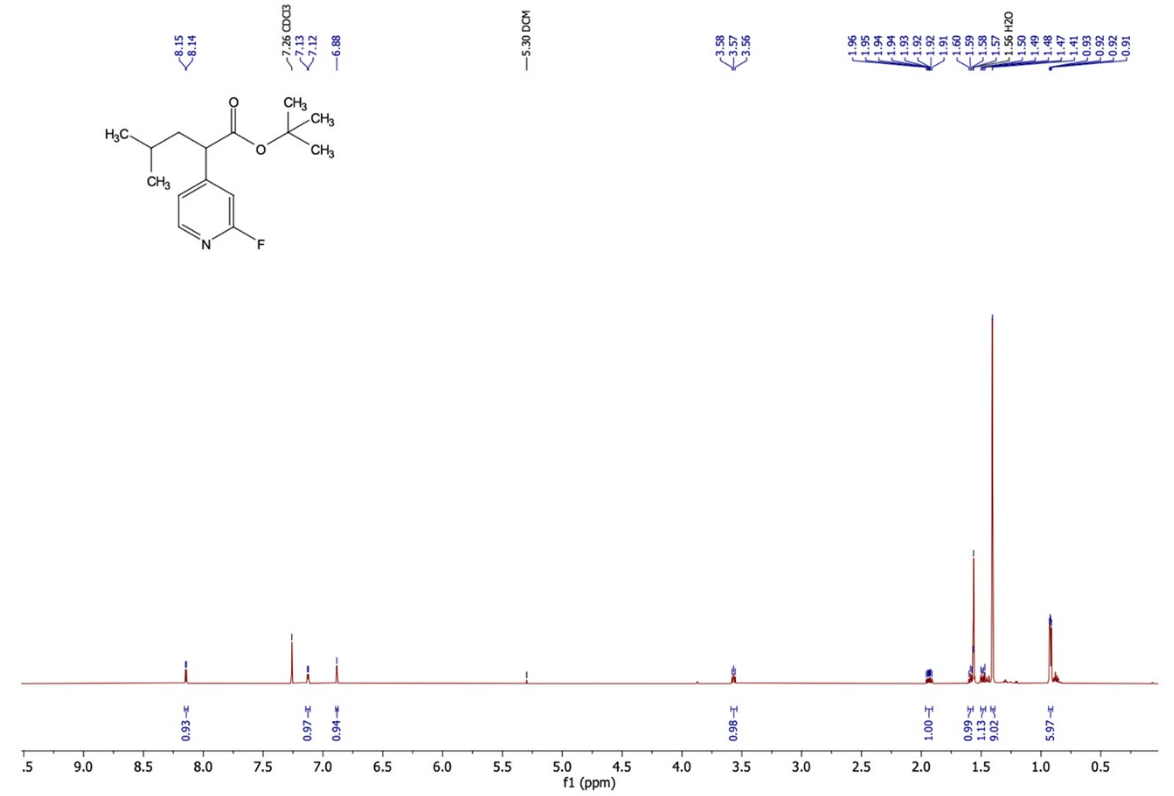

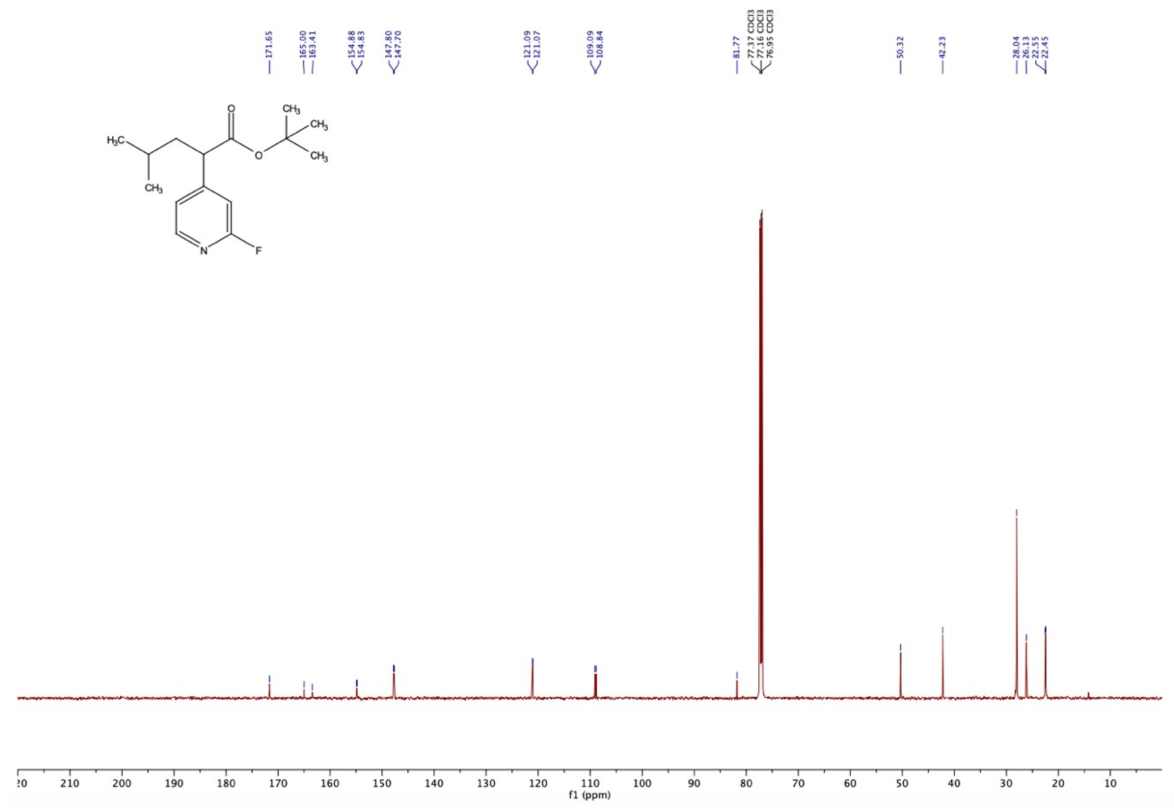

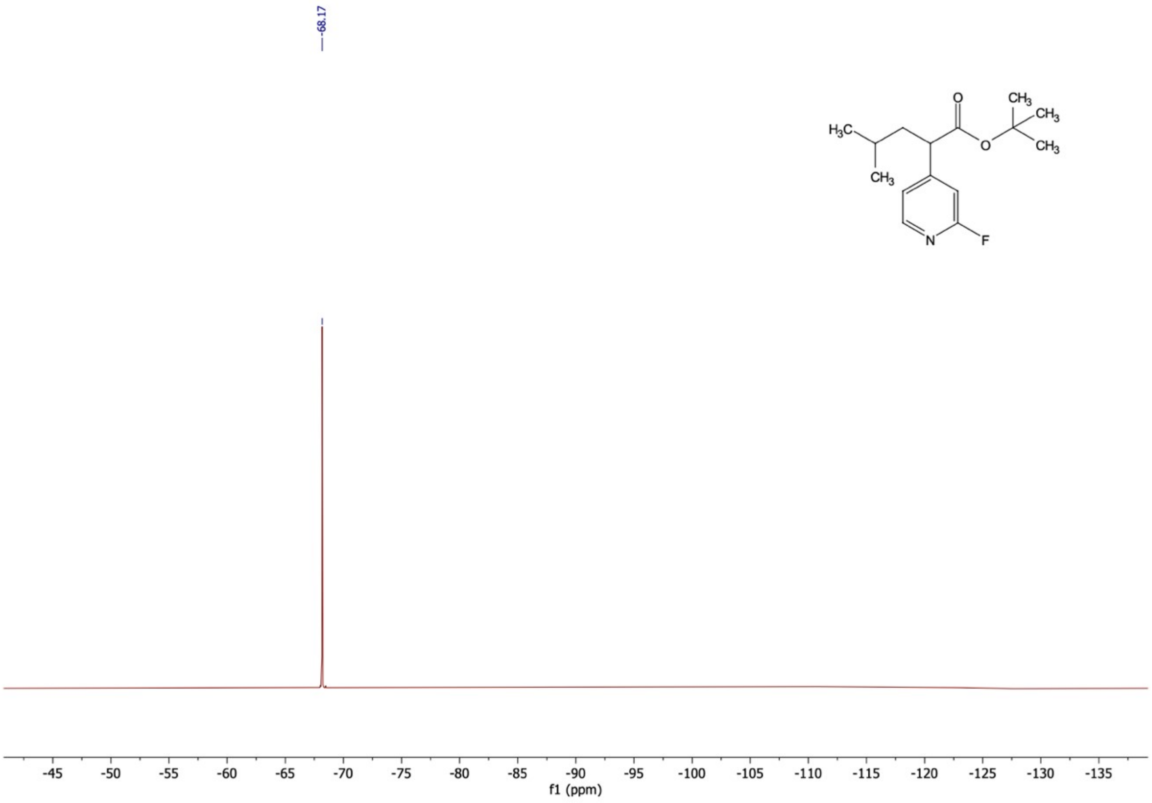

tert-Butyl 4-ethyl-2-(2-fluoropyridin-4-yl)heptanoate (38)

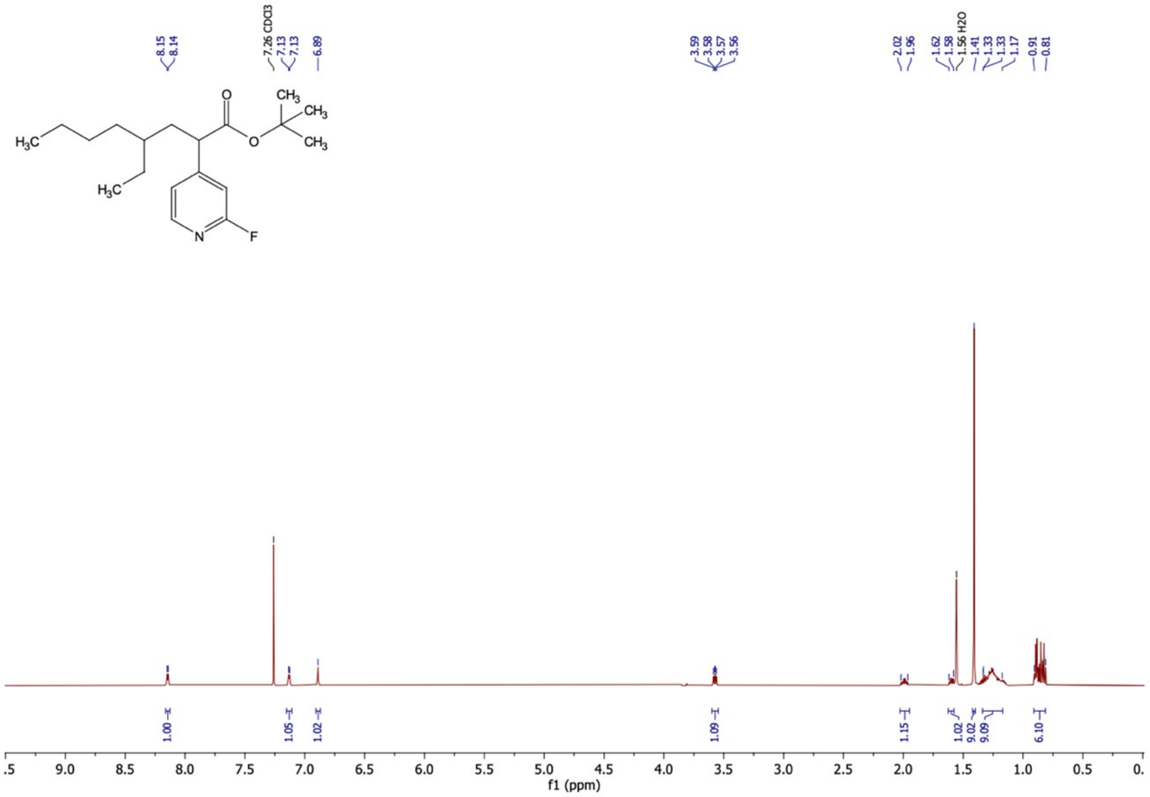

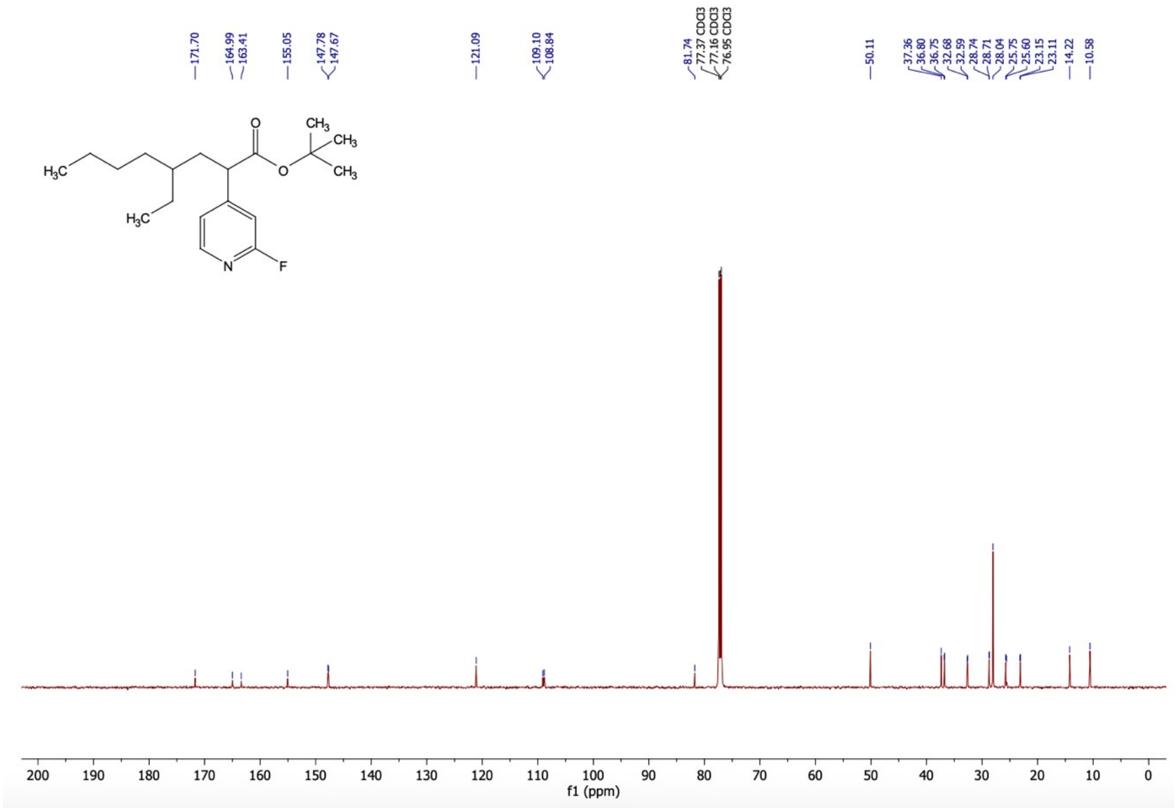
171.70
164.99
163.41
155.05
147.78
147.67
121.09
109.10
108.84
81.74
77.37 CDCl3
77.16 CDCl3
76.95 CDCl3
50.11
37.36
36.80
36.75
32.68
32.59
28.74
28.71
28.04
25.75
25.60
23.15
23.11
14.22
10.58
f1 (ppm)

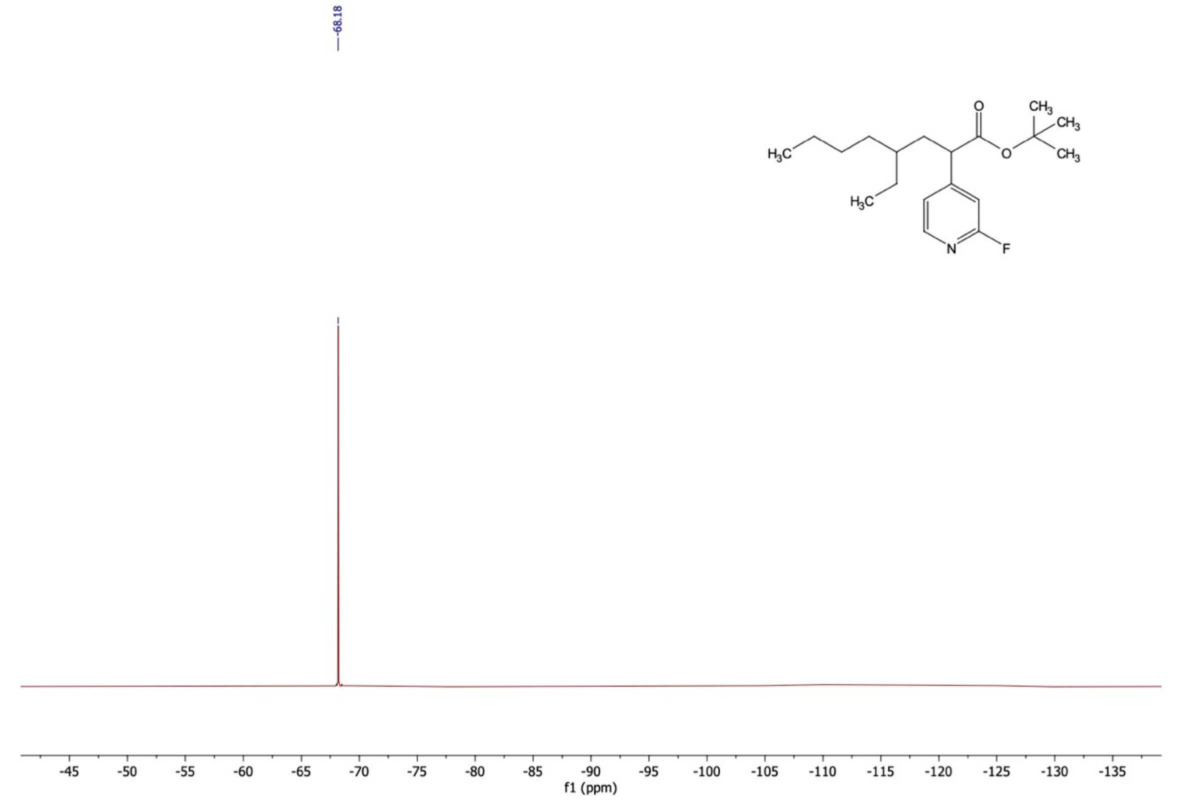
-68.18
f1 (ppm)

NMR spectra for yield determination of non-isolated products

Phenyl 2-(4-acetylphenyl)-4,4-dimethylpentanoate (4)

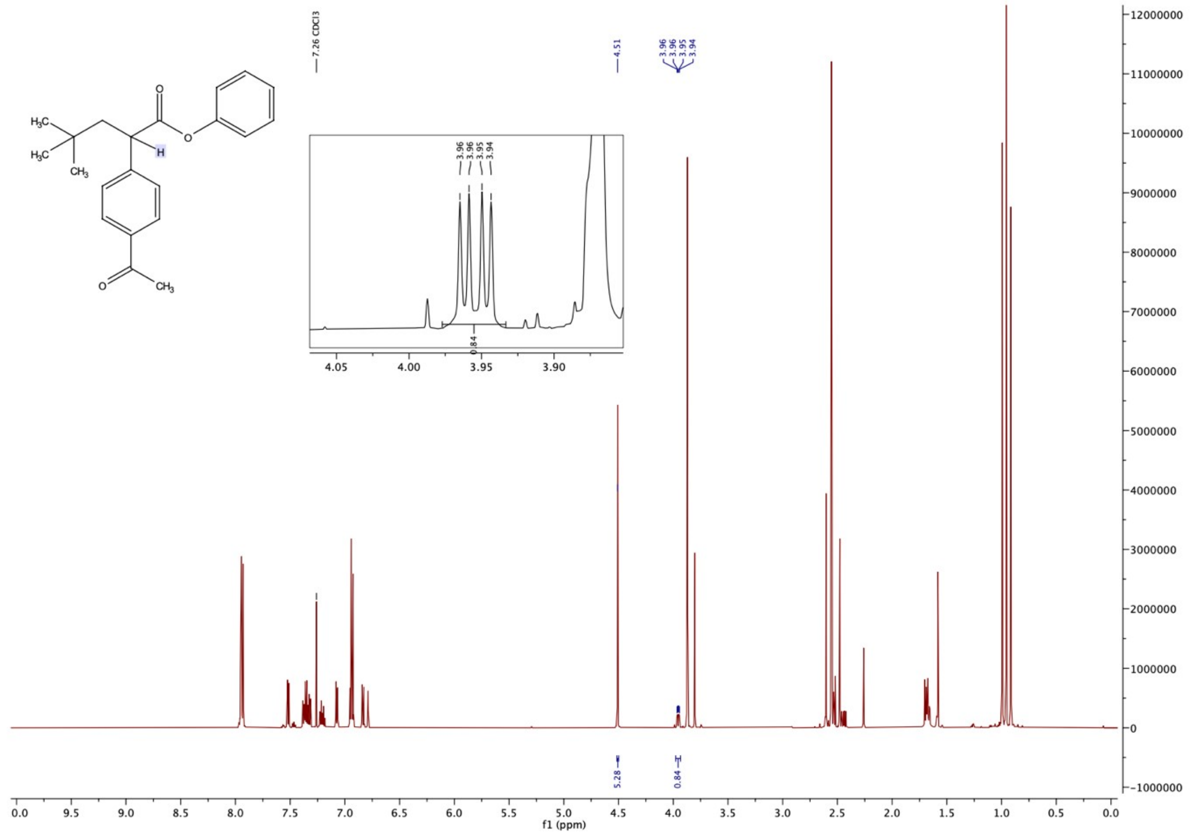

tert-butyl 2-(4-formylphenyl)-4,4-dimethylpentanoate (11)

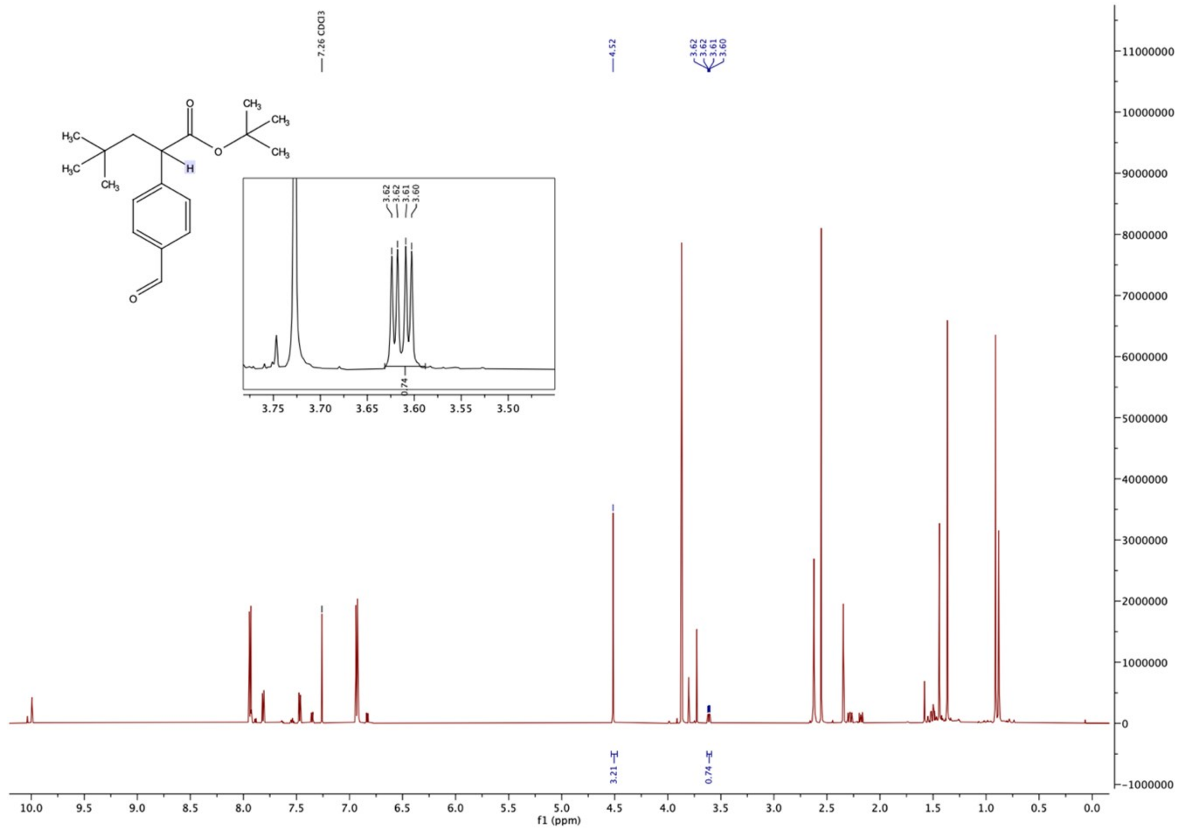

tert-butyl 4,4-dimethyl-2-(4-(4,4,5,5-tetramethyl-1,3,2-dioxaborolan-2-yl)phenyl)pentanoate (12)

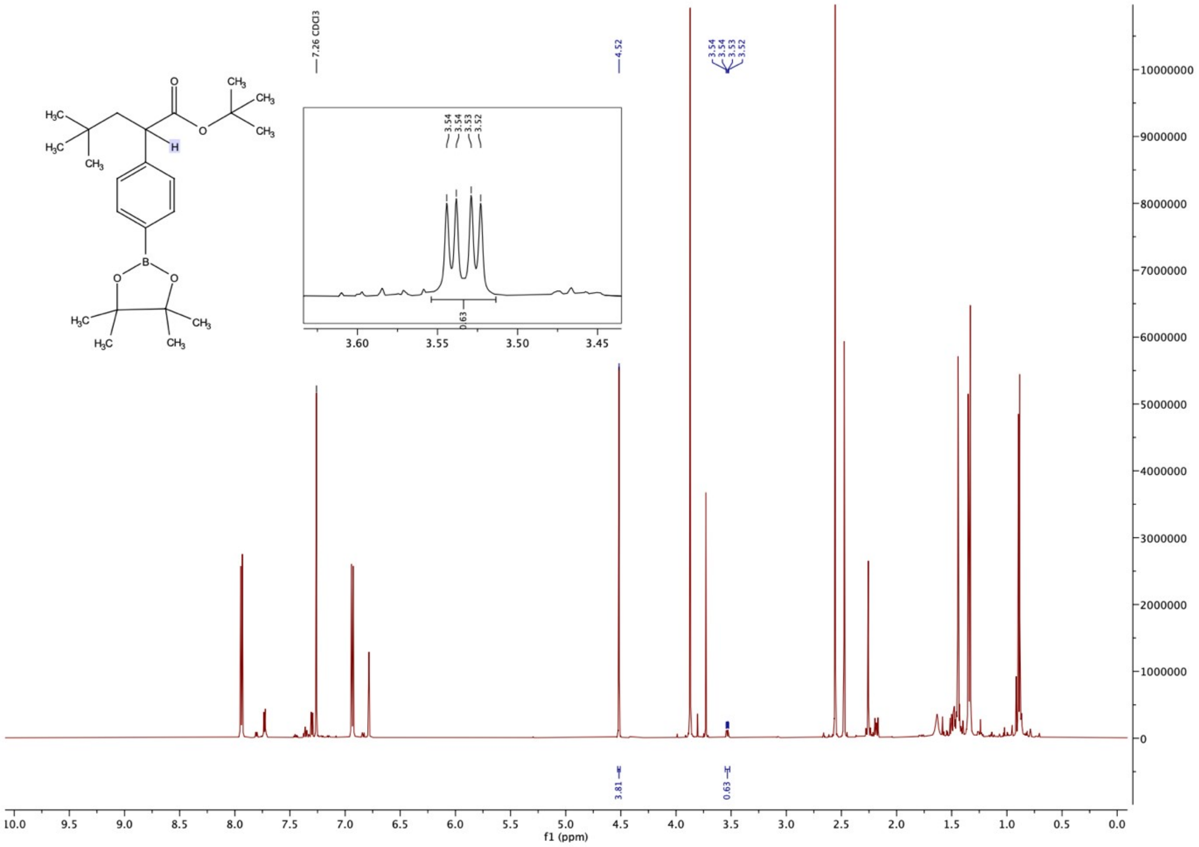

Phenyl 2-(4-formylphenyl)-4,4-dimethylpentanoate (13)

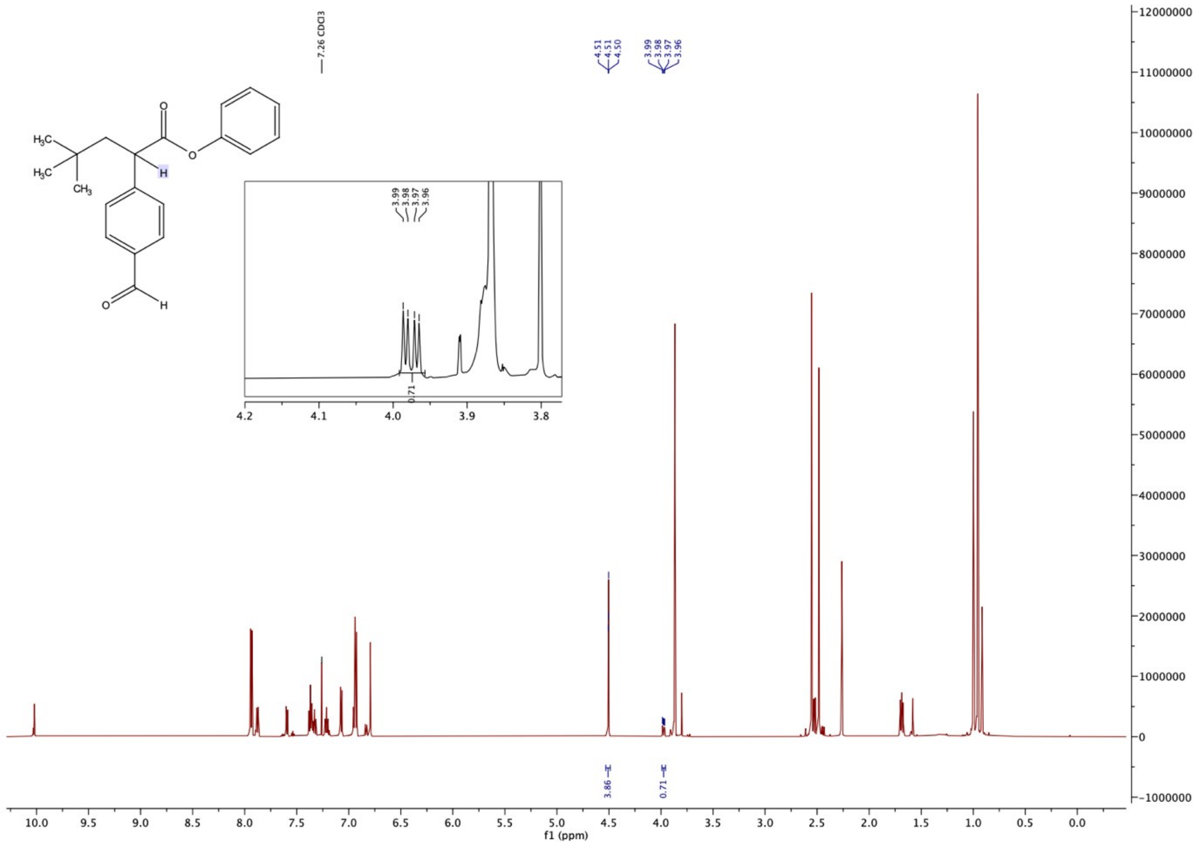

Phenyl 2-(4-cyanophenyl)-4,4-dimethylpentanoate (14)

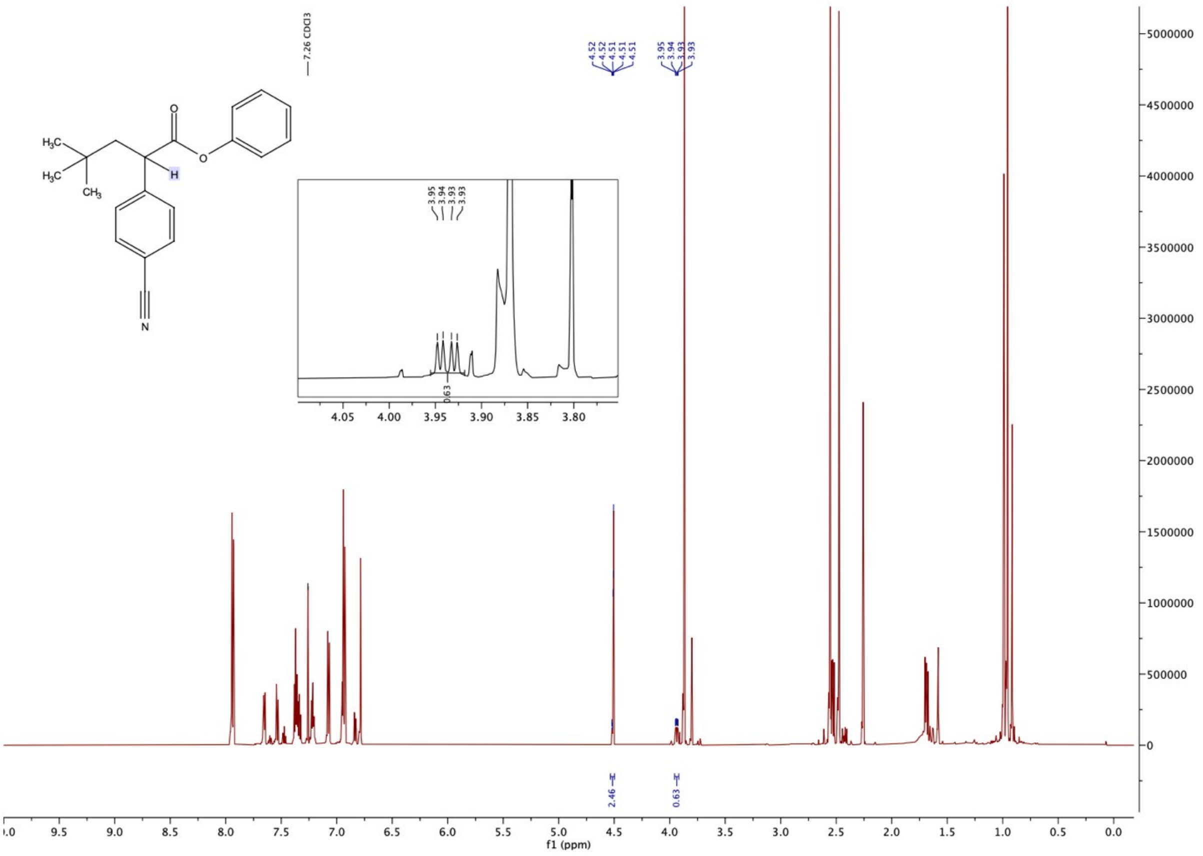

Phenyl 4,4-dimethyl-2-(4-(trifluoromethyl)phenyl)pentanoate (15)

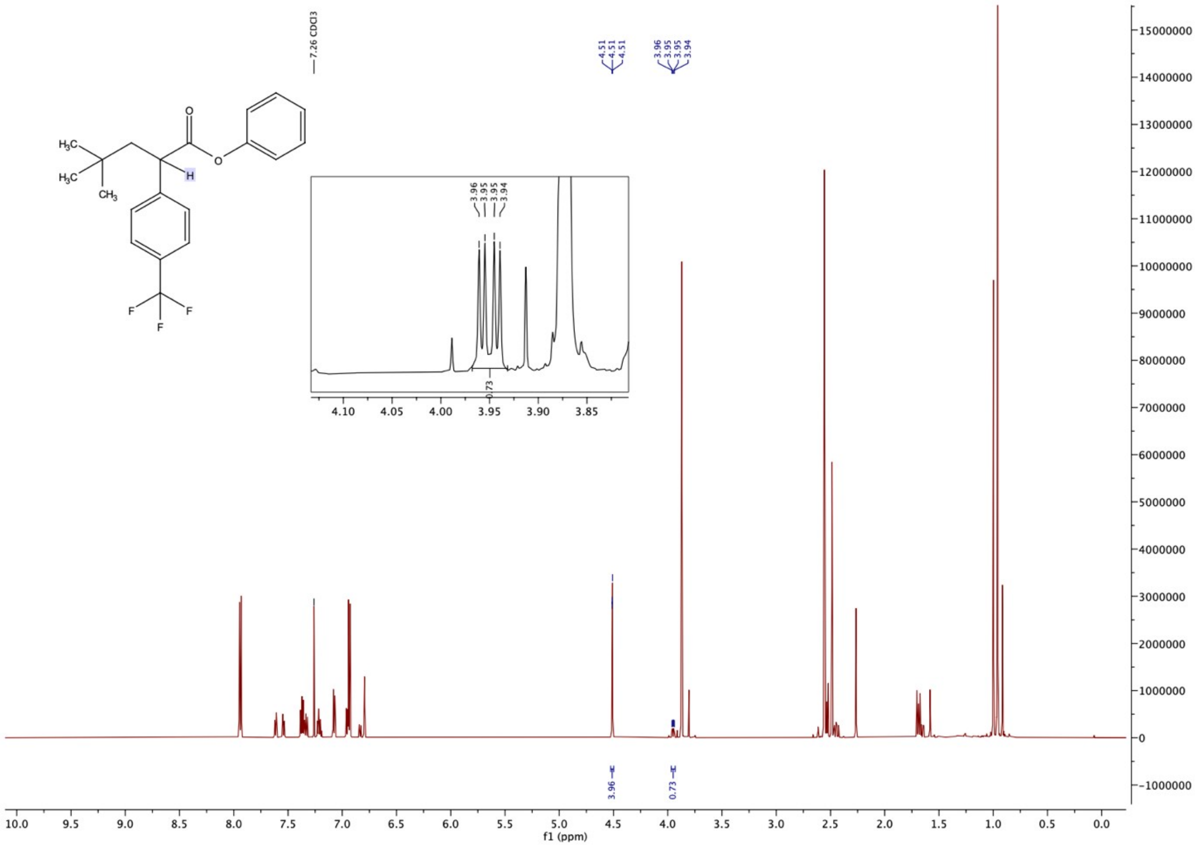

Phenyl 4,4-dimethyl-2-phenylpentanoate (16)

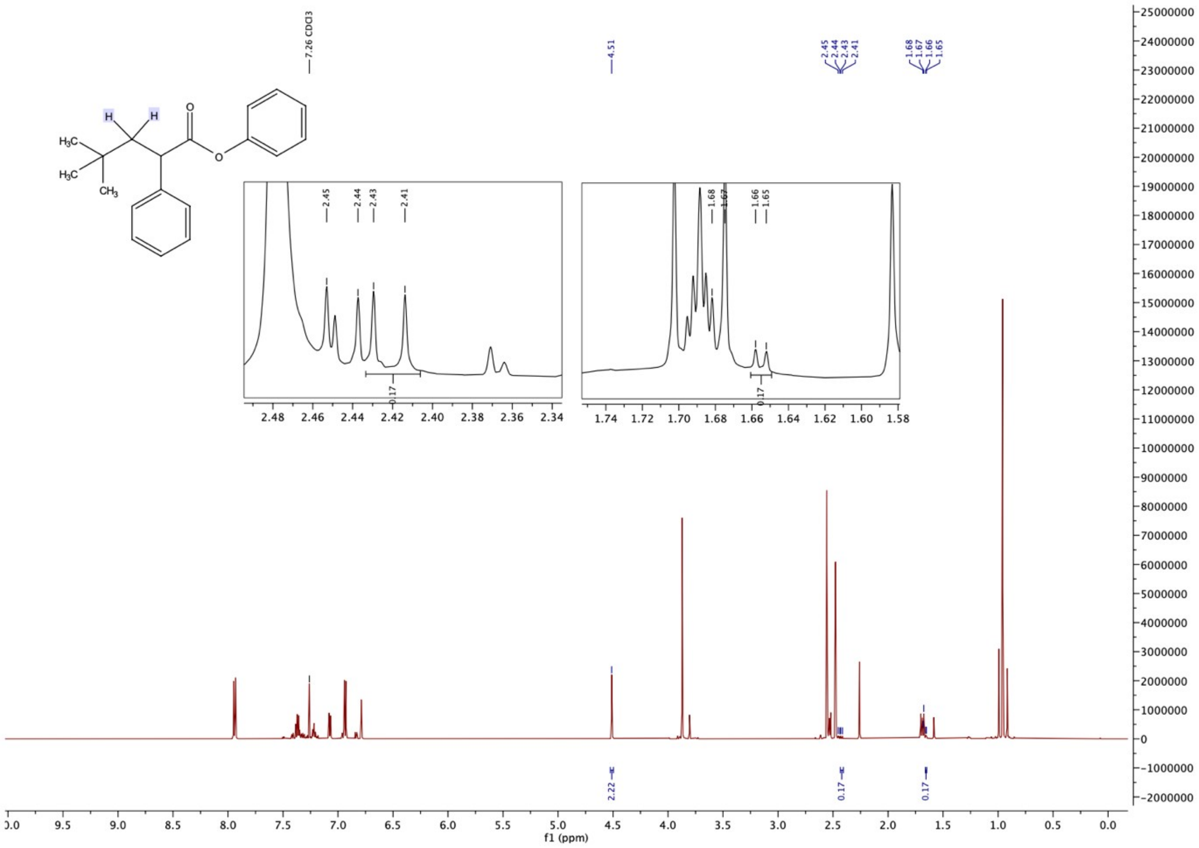

Phenyl 2-(4-methoxyphenyl)-4,4-dimethylpentanoate (17)

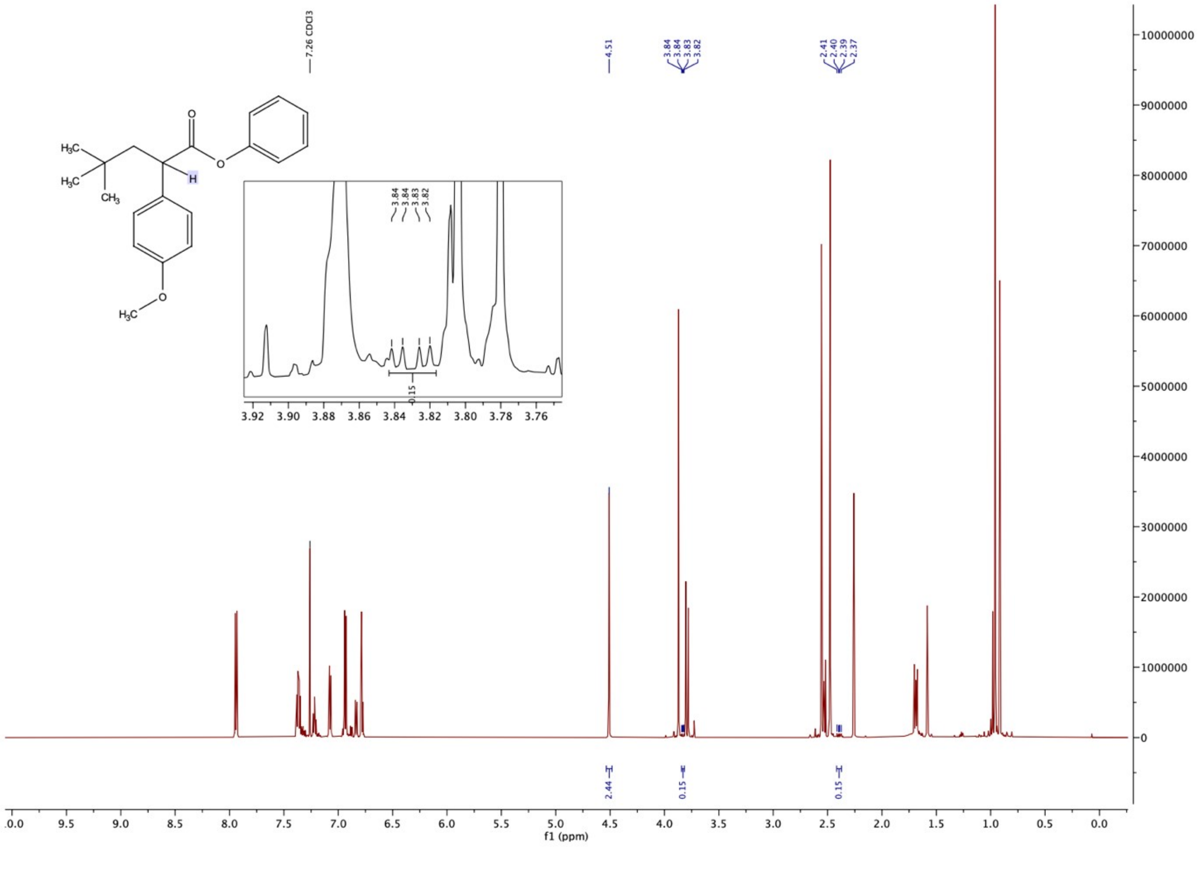

Phenyl 4,4-dimethyl-2-(*p*-tolyl)pentanoate (18)

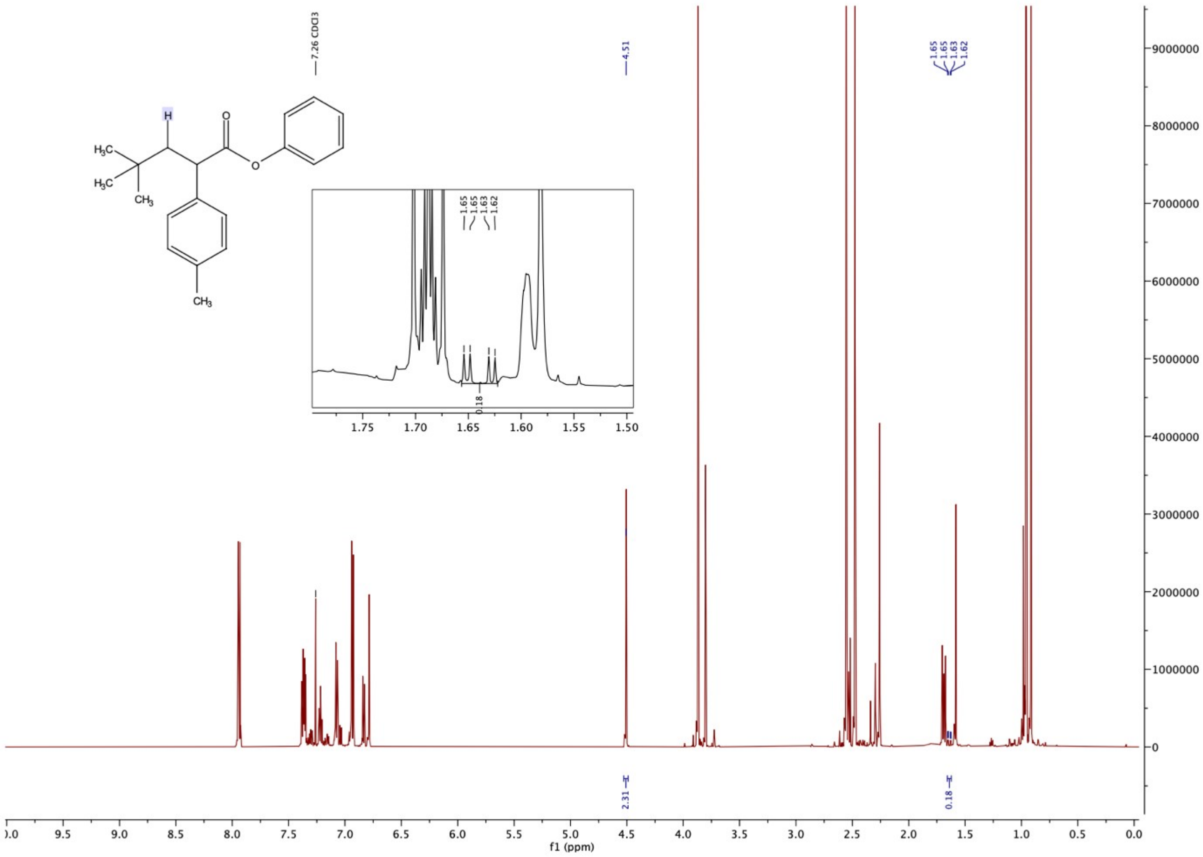

***tert*-butyl 4,4-dimethyl-2-(1-oxo-1,3-dihydroisobenzofuran-5-yl)pentanoate (21)**

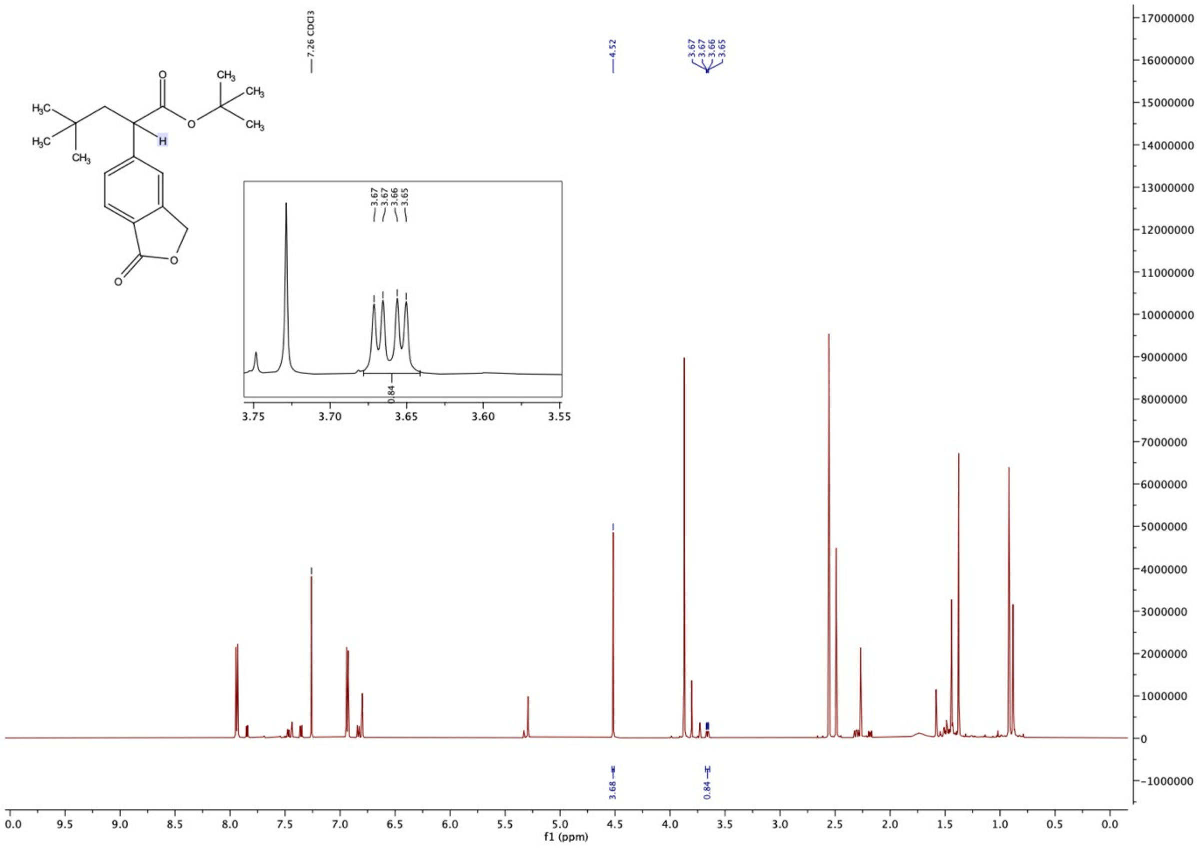

tert-butyl 4,4-dimethyl-2-(2-methylpyrimidin-5-yl)pentanoate (24)

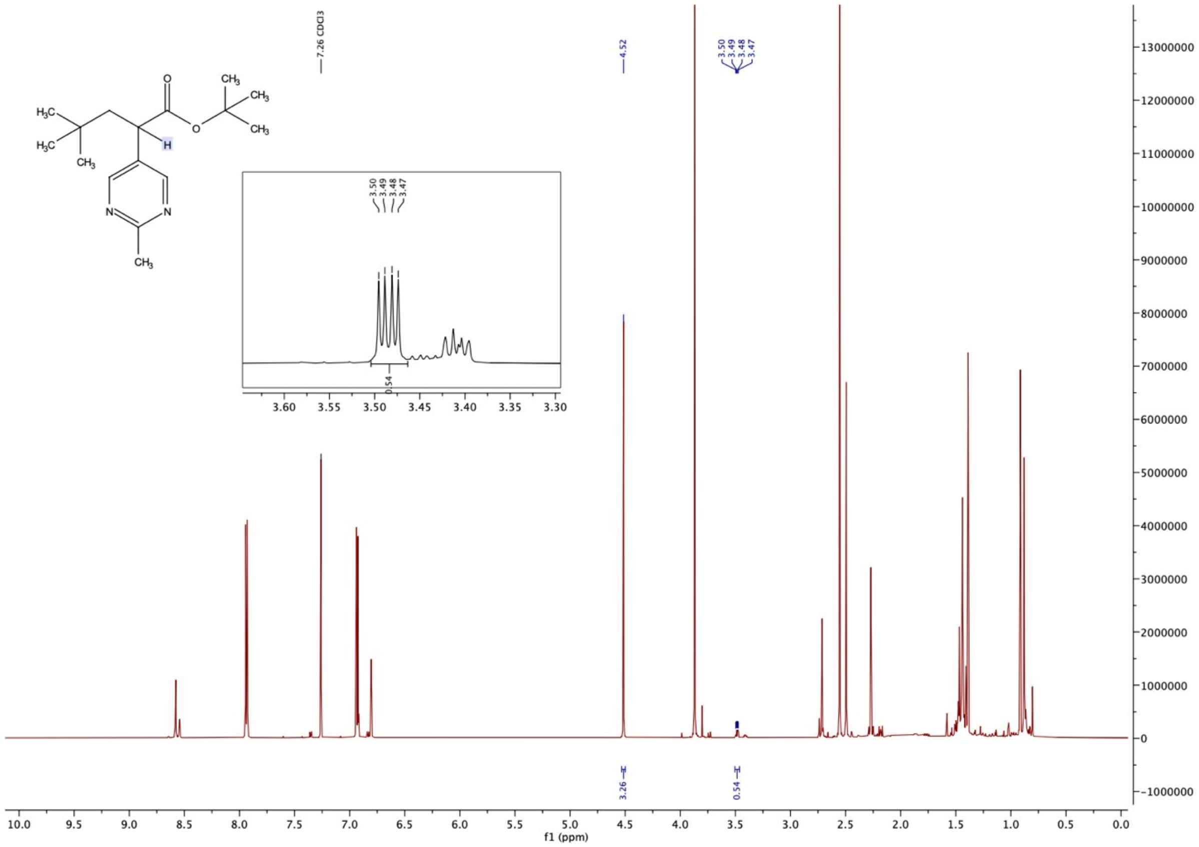

tert-butyl 4,4-dimethyl-2-(quinolin-6-yl)pentanoate (25)

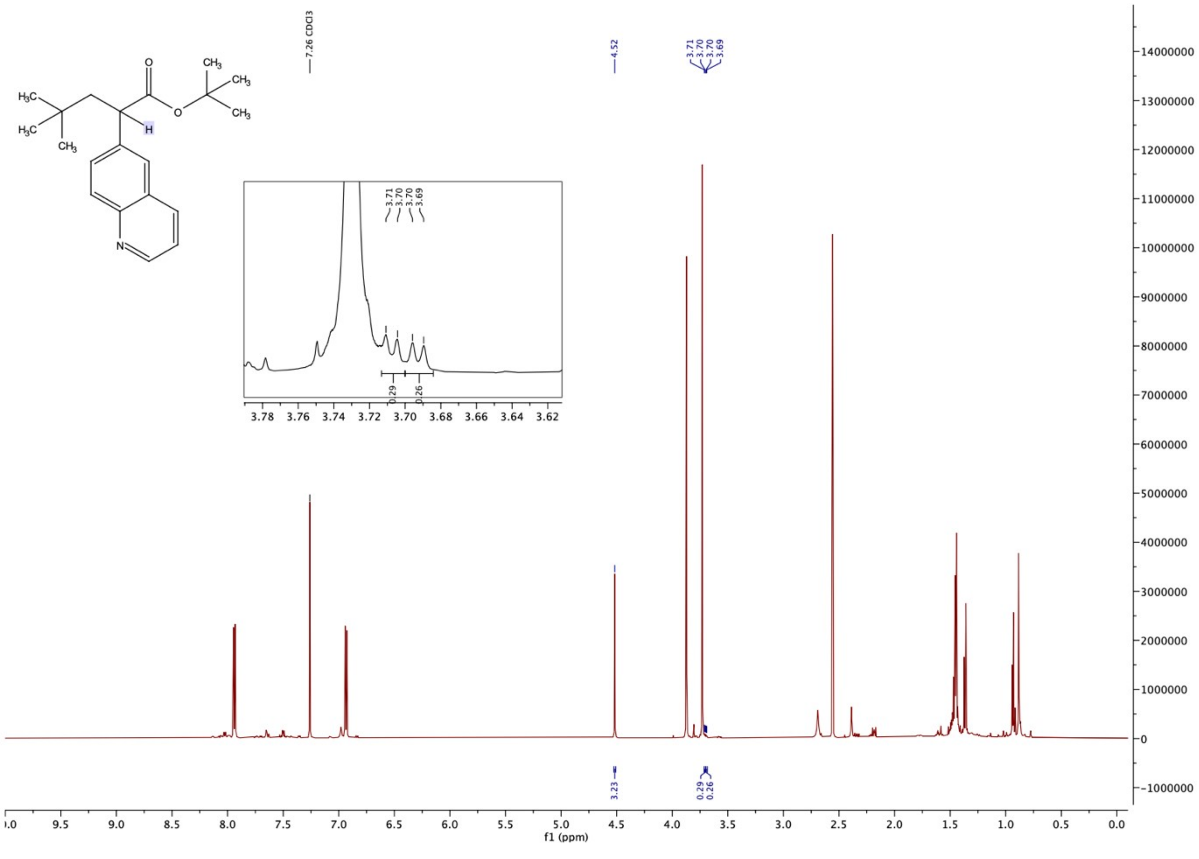

1-(4-(3,3-dimethyl-1-(4,4,5,5-tetramethyl-1,3,2-dioxaborolan-2-yl)butyl)phenyl)ethan-1-one (28)

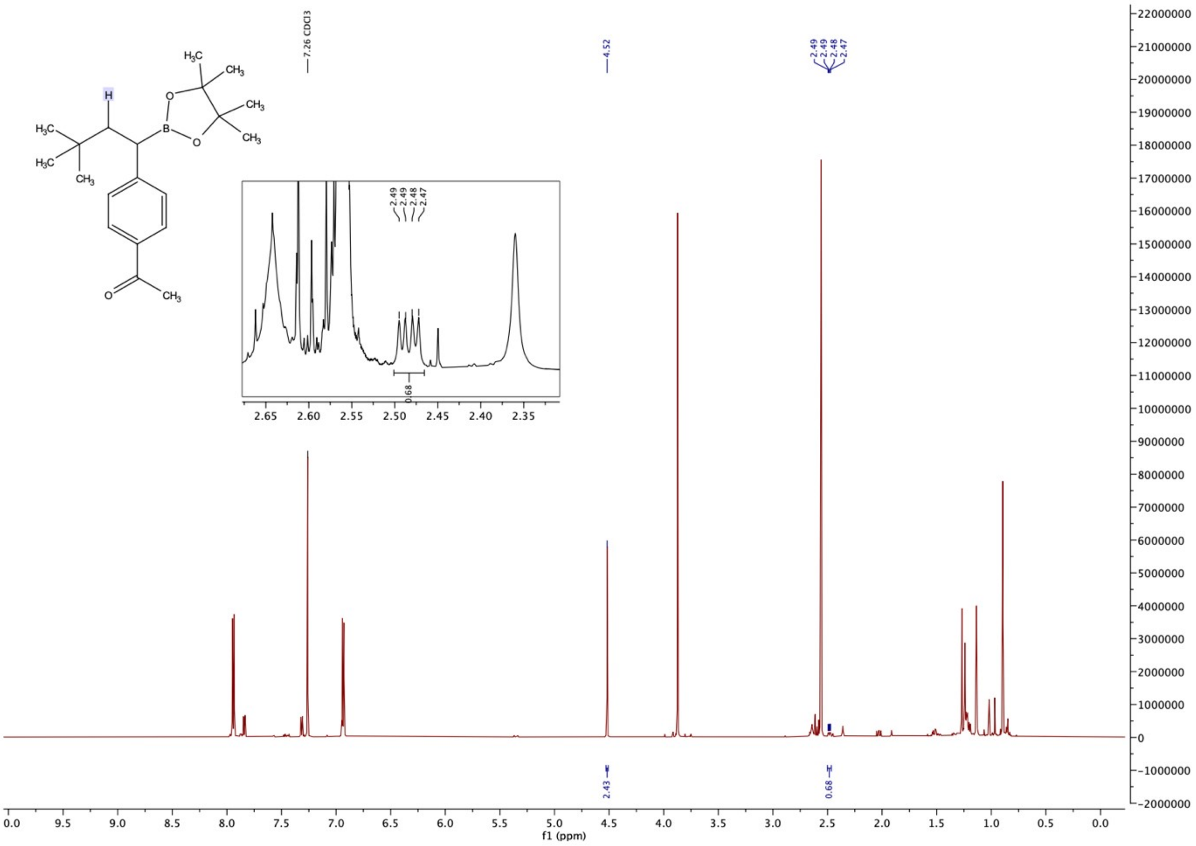

2-(4-acetylphenyl)-*N*,*N*,4,4-tetramethylpentanamide (29)

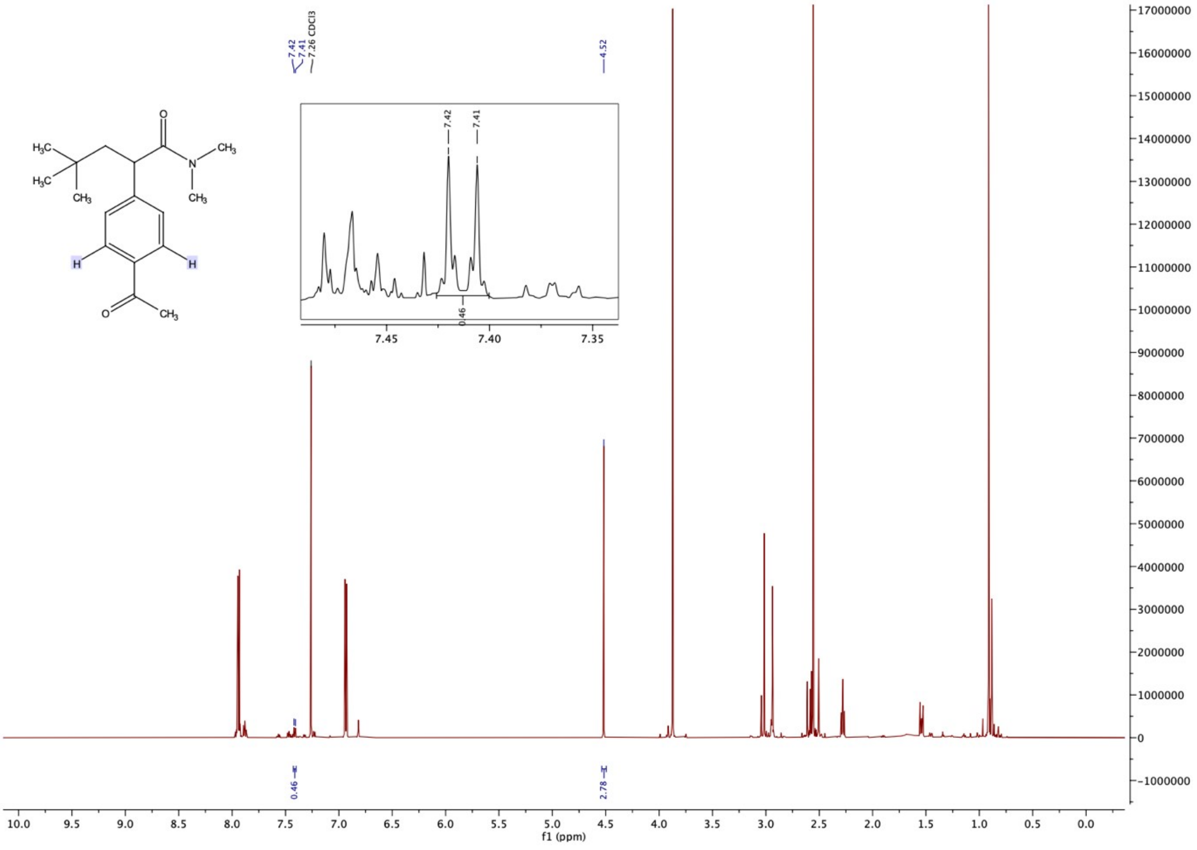

1-(4-(3,3-dimethyl-1-phenylbut-1-en-1-yl)phenyl)ethan-1-one (30)

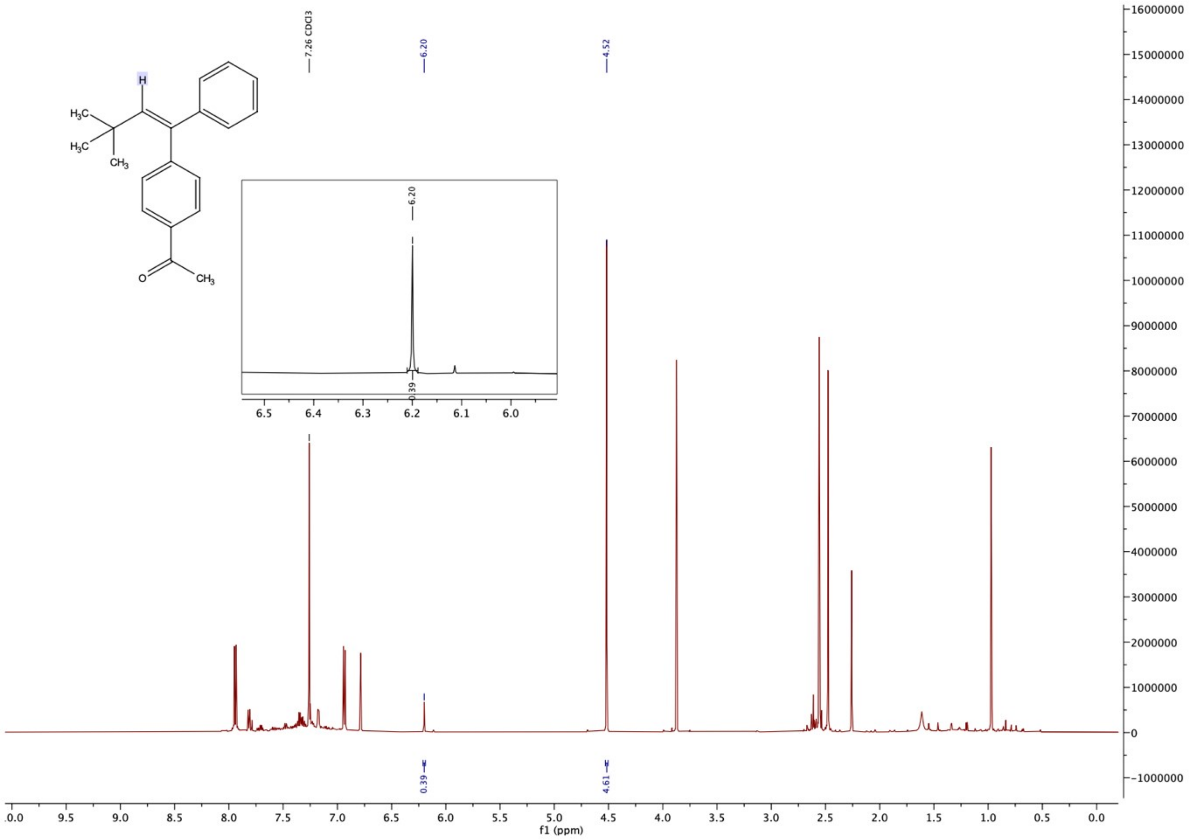

1-(4-(3,3-dimethyl-1-(*p*-tolyl)but-1-en-1-yl)phenyl)ethan-1-one (31)

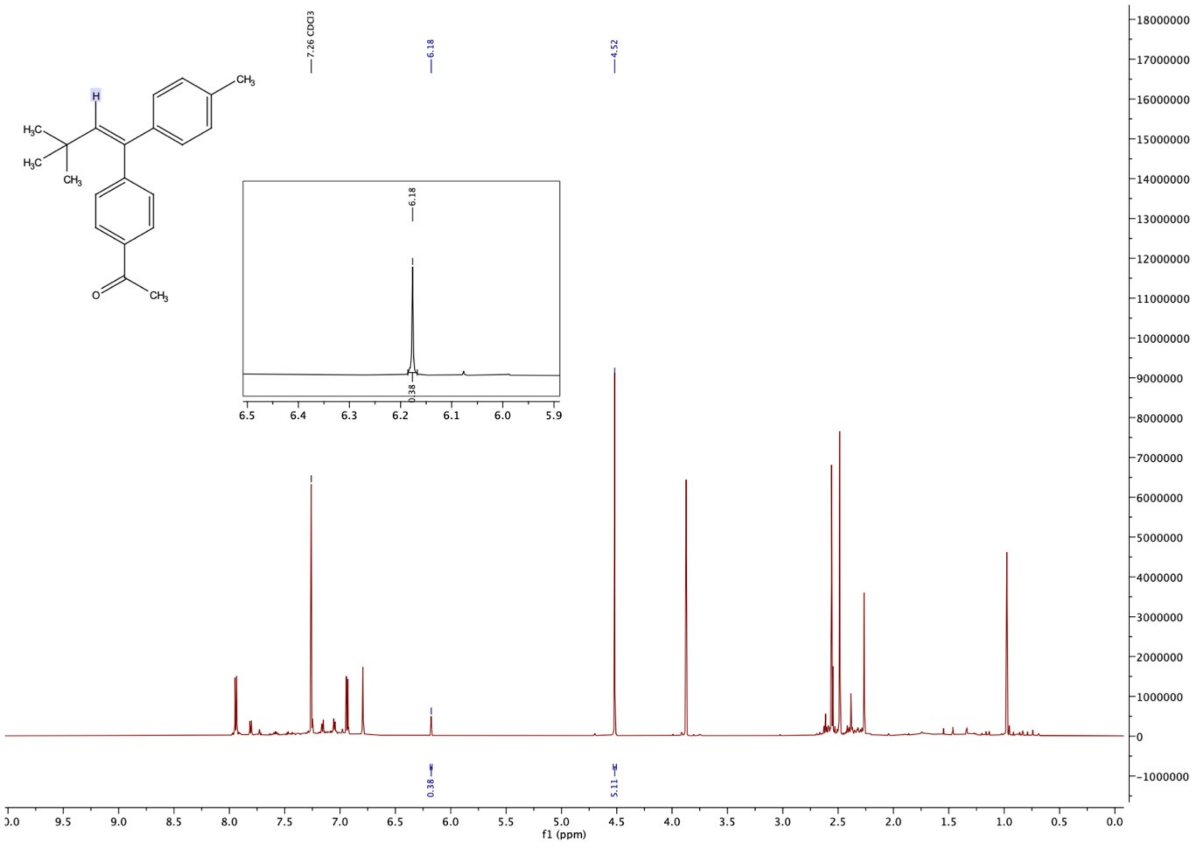

1-(4-(1-(4-chlorophenyl)-3,3-dimethylbut-1-en-1-yl)phenyl)ethan-1-one (32)

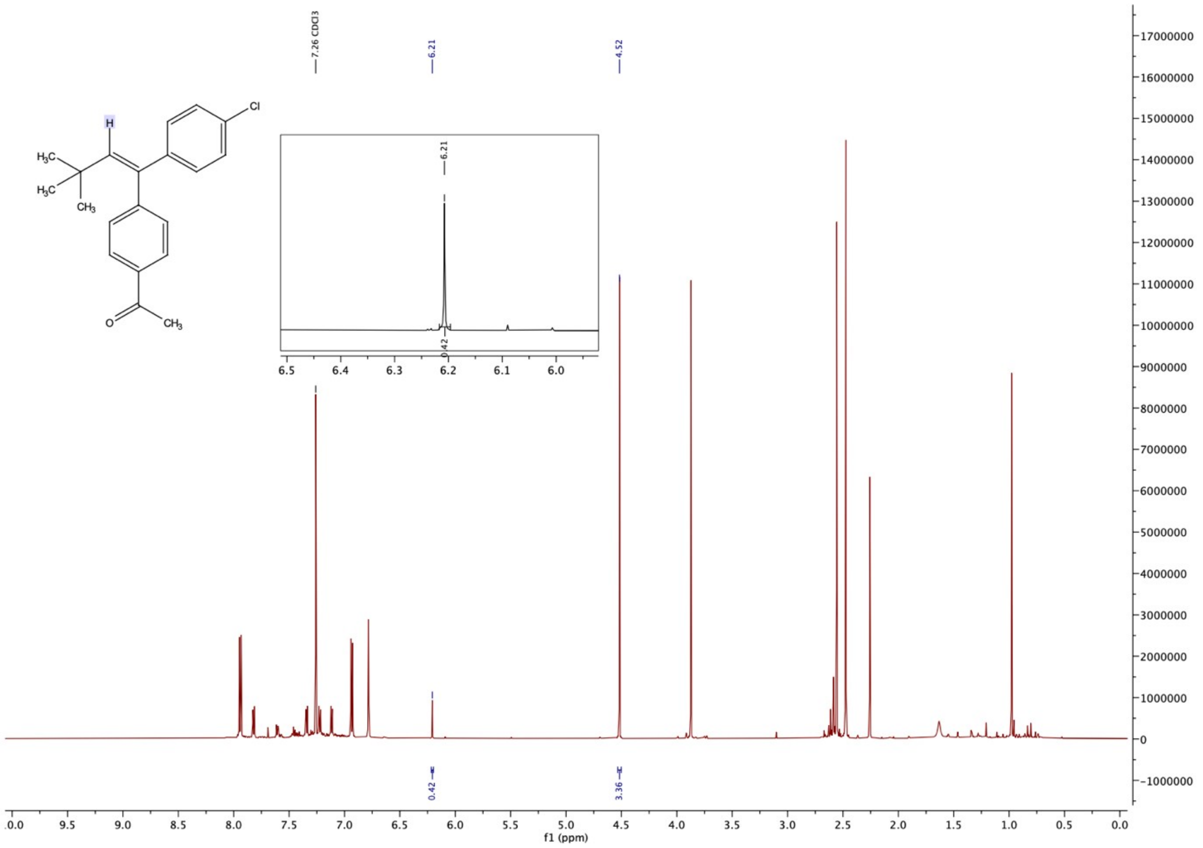

***tert*-butyl 4-(3-(*tert*-butoxy)-2-(2-fluoropyridin-4-yl)-3-oxopropyl)piperidine-1-carboxylate (35)**

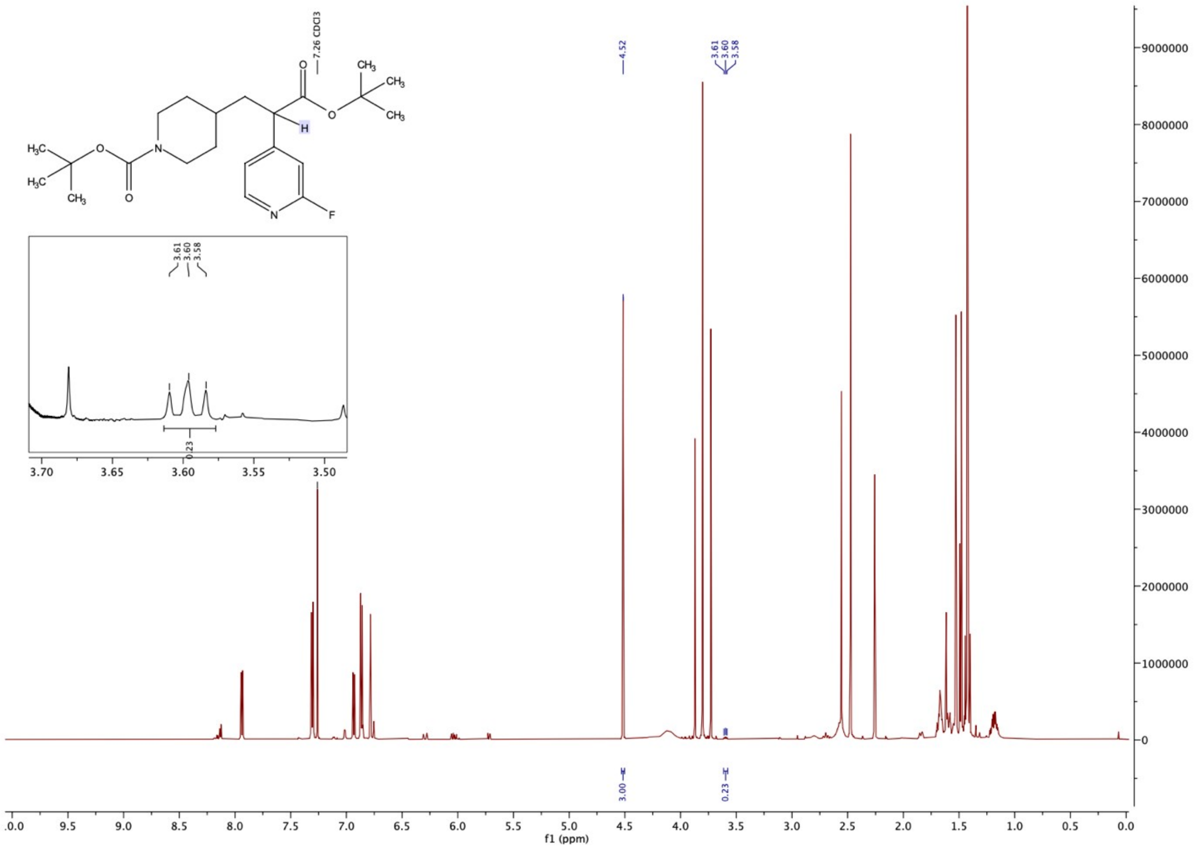

***tert*-butyl 2-(2-fluoropyridin-4-yl)-3-(tetrahydro-2*H*-pyran-4-yl)propanoate (36)**

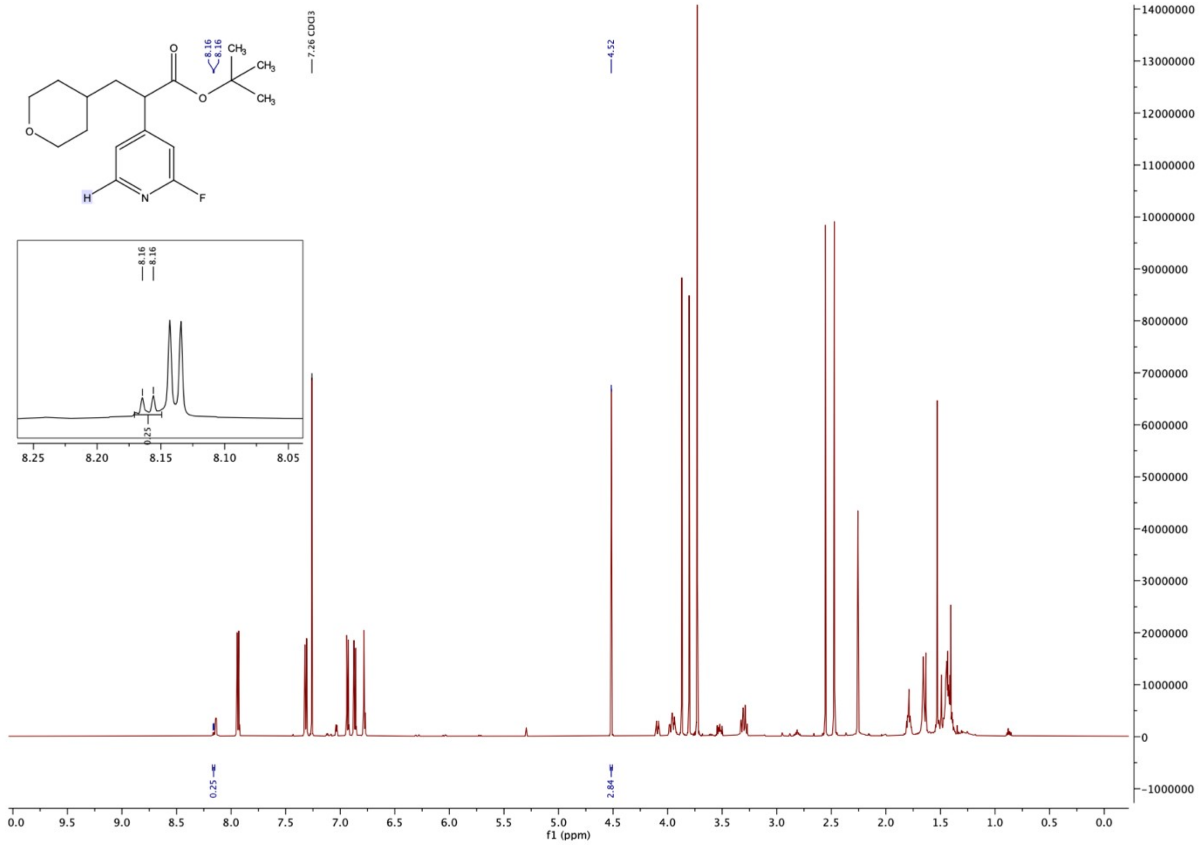

***tert*-butyl 2-(2-fluoropyridin-4-yl)-4-phenylpentanoate (39)**

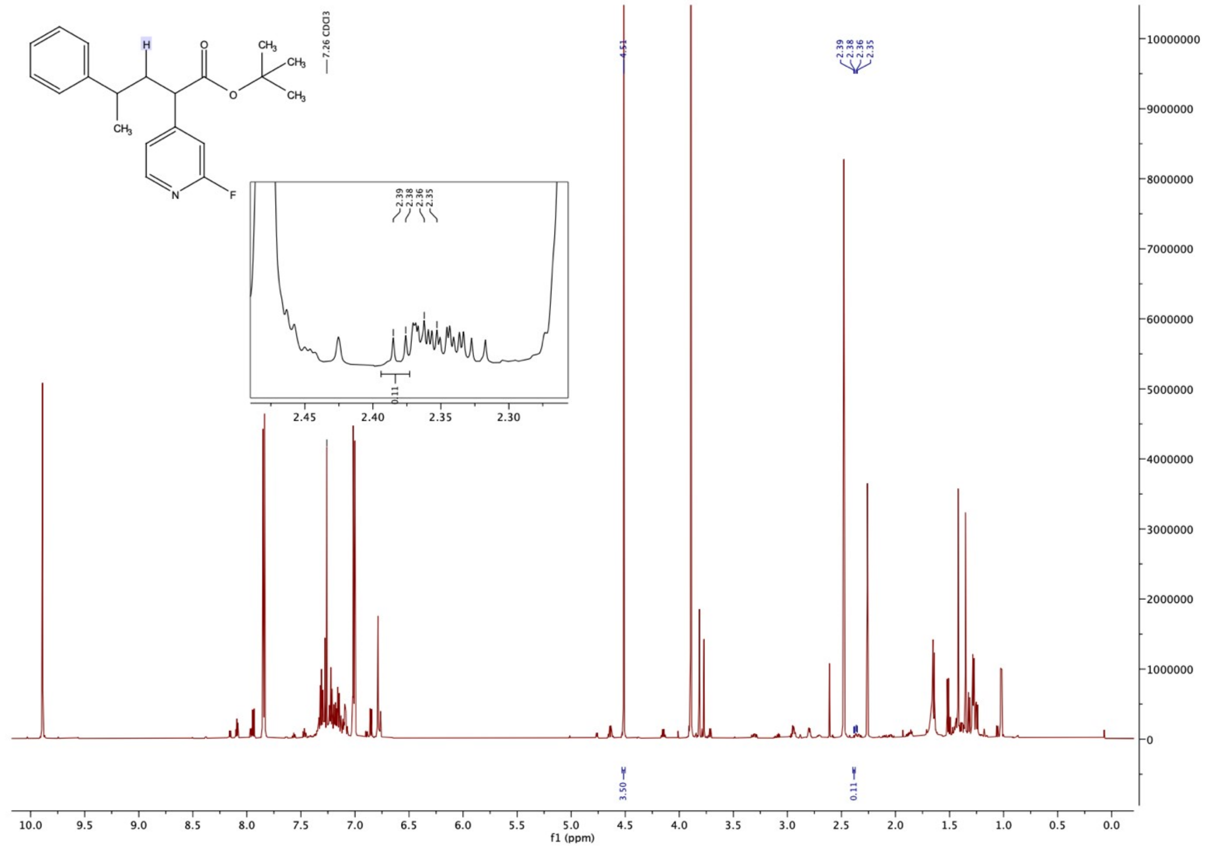

NMR spectra of crude reaction mixtures illustrating mass balance of compounds 4, 33, and 40

Phenyl 2-(4-acetylphenyl)-4,4-dimethylpentanoate (4)

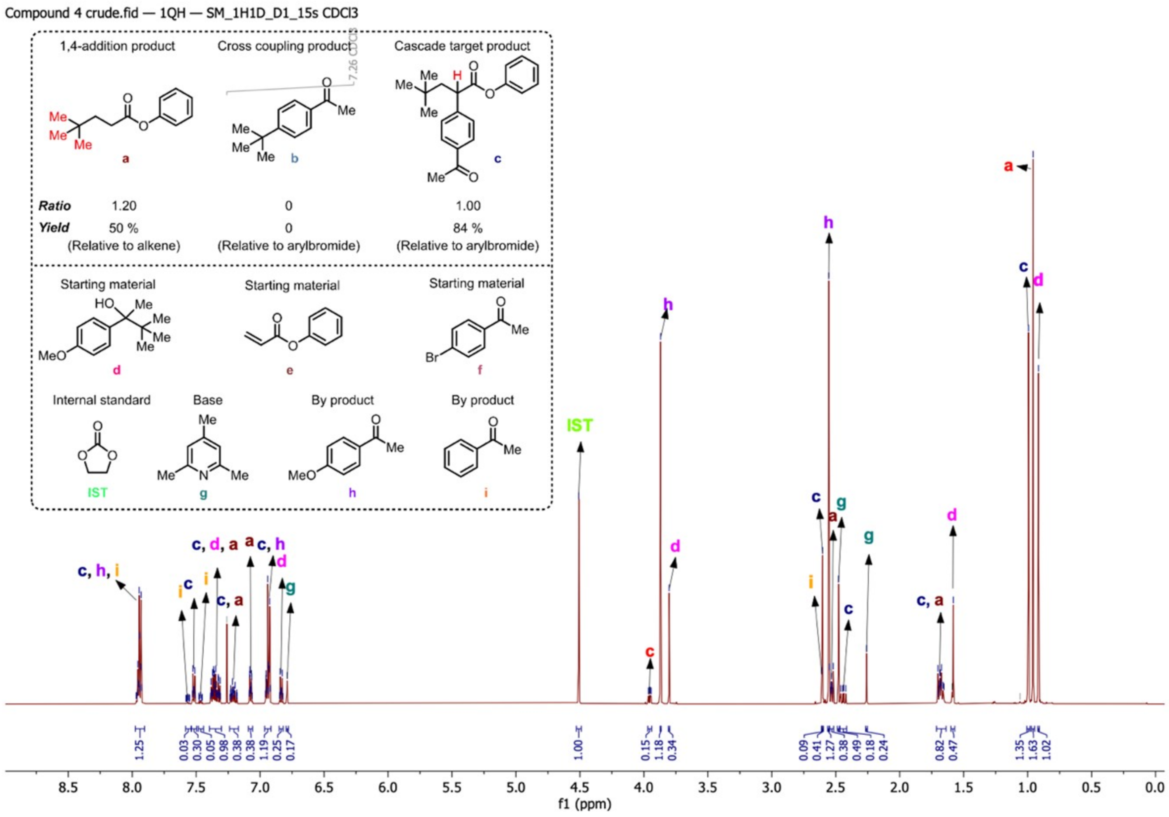

Phenyl 2-(4-acetylphenyl)-4,4-dimethylpentanoate (4)

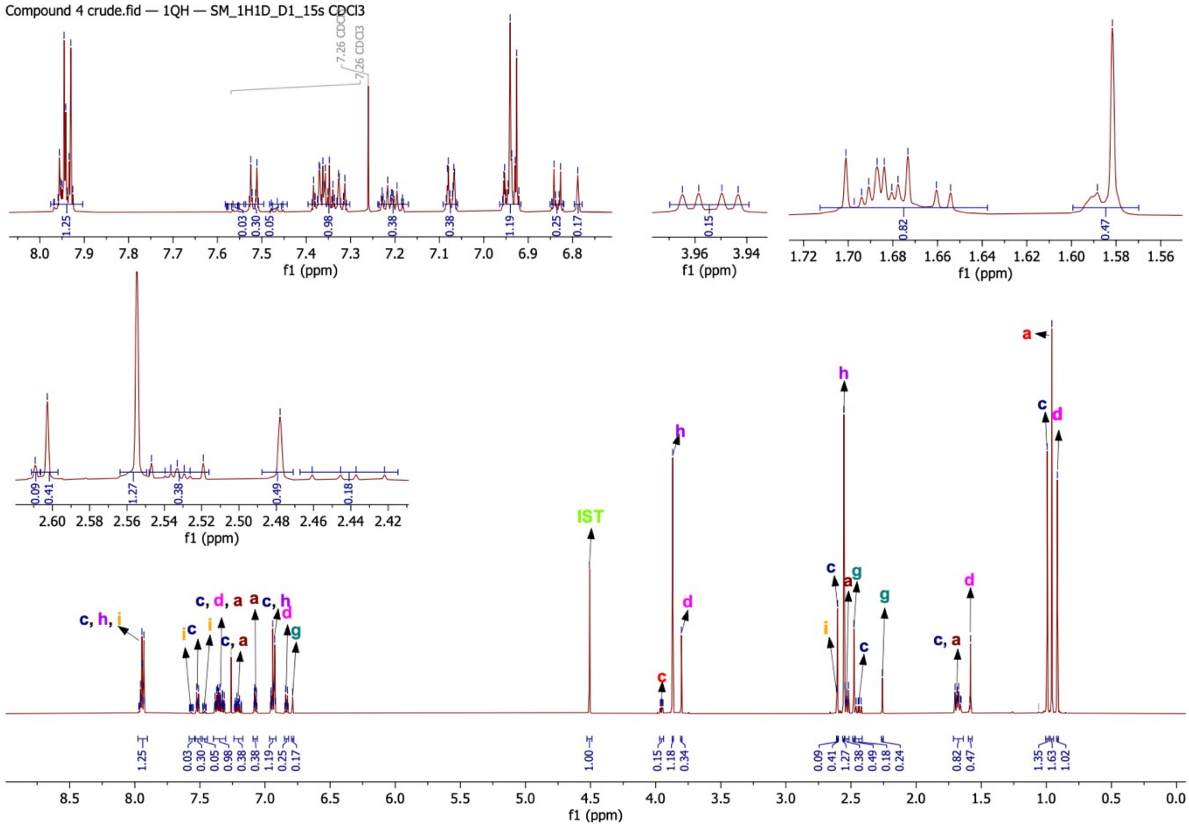

tert-Butyl 3-cyclohexyl-2-(2-fluoropyridin-4-yl)propanoate (33)

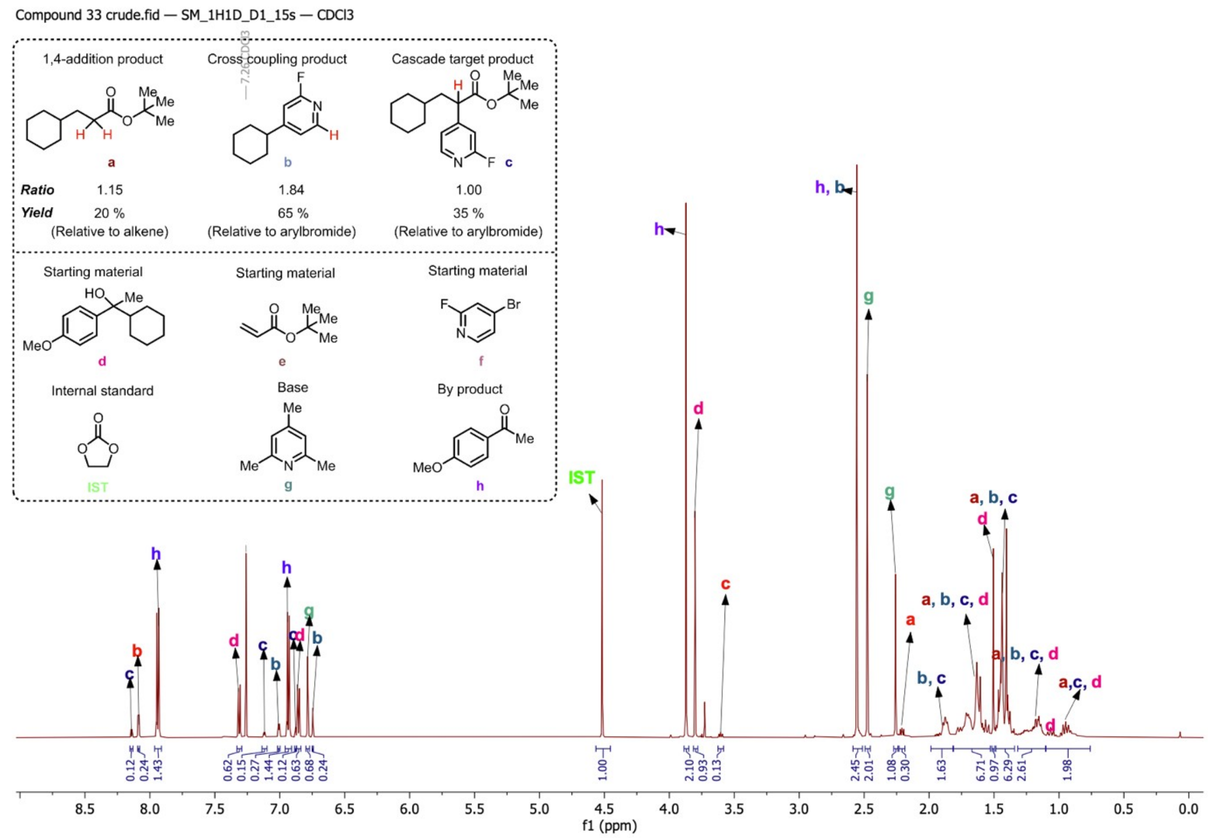

***tert*-Butyl 3-cyclohexyl-2-(2-fluoropyridin-4-yl)propanoate (33)**

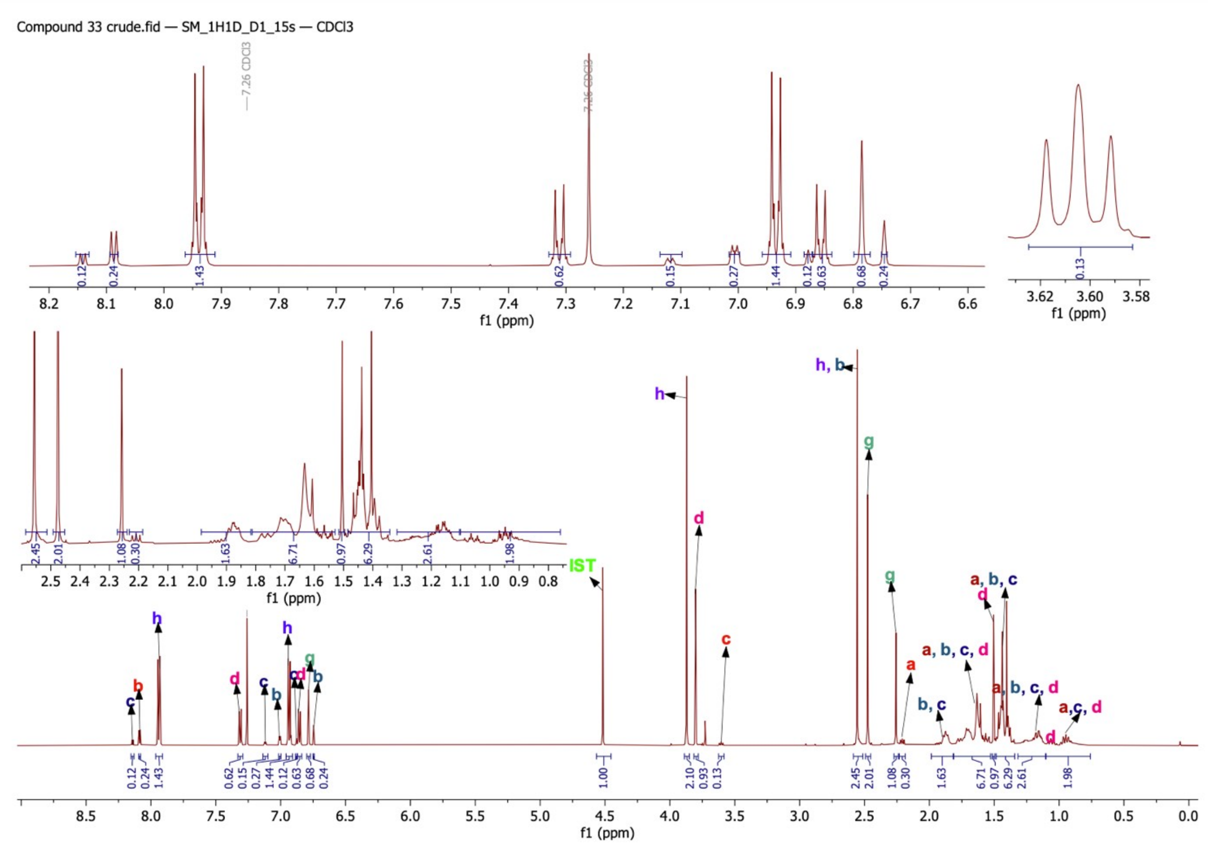

***tert*-butyl 2-(2-fluoropyridin-4-yl)heptanoate (40)**

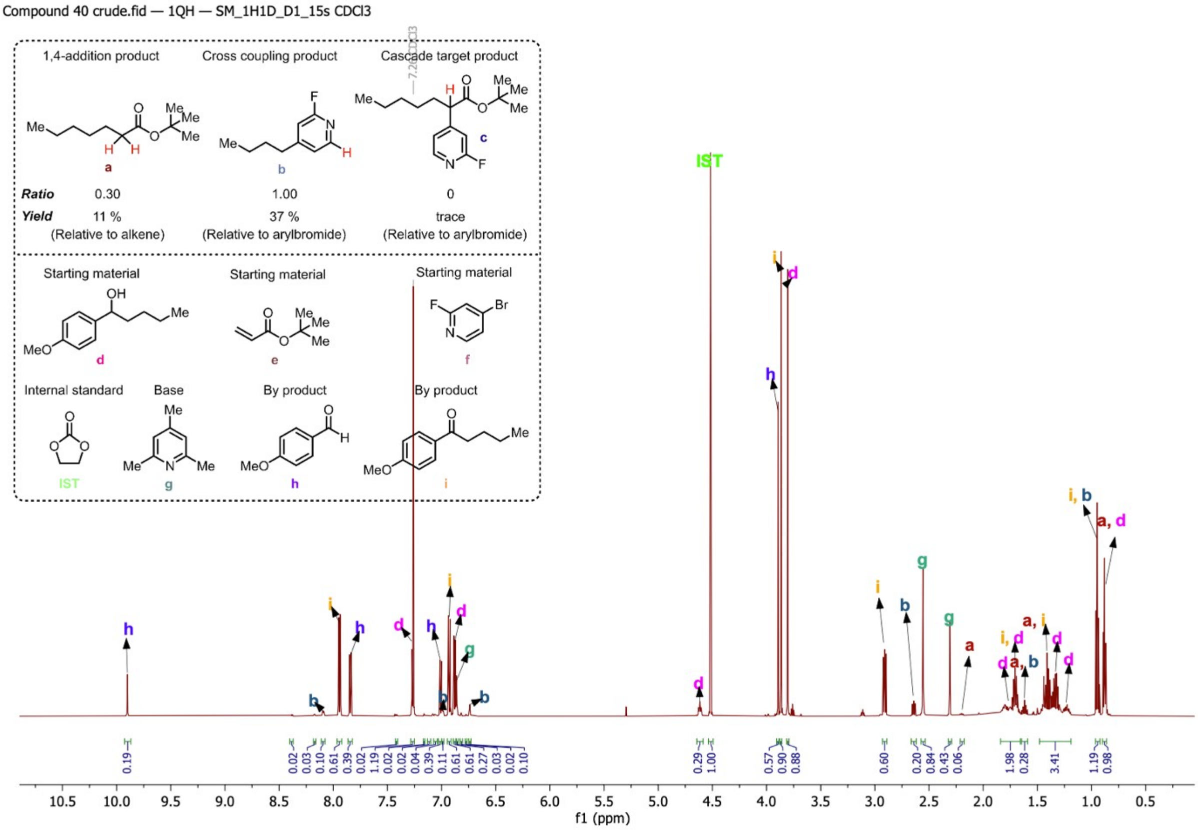

***tert*-butyl 2-(2-fluoropyridin-4-yl)heptanoate (40)**

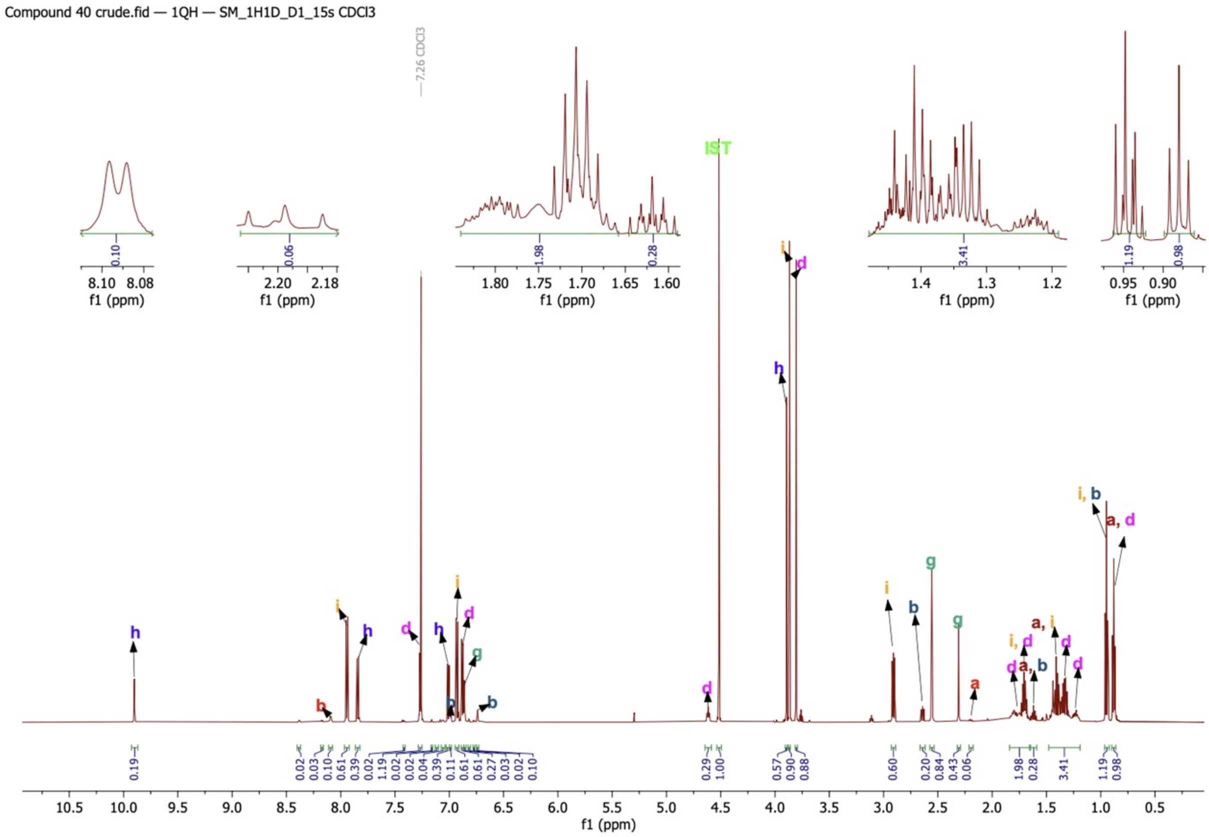

Supplement: SC-017-D6SC00382F-s001 [file SC-017-D6SC00382F-s001.pdf]
